# Exploration of effective pharmacological inhibitors for NS5 protein through computational approach: A strategy to combat the neglected Kyasanur forest disease virus

**DOI:** 10.1371/journal.pone.0325613

**Published:** 2025-07-10

**Authors:** Sharanappa Achappa, Nayef Abdulaziz Aldabaan, Mohammed Alasmary, Ibrahim Ahmed Shaikh, Mater H. Mahnashi, Shivalingsarj V. Desai, Uday M. Muddapur, Aejaz Abdullatif Khan, Basheerahmed Abdulaziz Mannasaheb

**Affiliations:** 1 Department of Biotechnology, KLE Technological University, Hubballi, Karnataka, India; 2 Department of Pharmacology, College of Pharmacy, Najran University, Najran, Saudi Arabia; 3 Department of Medicine, College of Medicine, Najran University, Najran, Saudi Arabia; 4 Department of Pharmaceutical Chemistry, College of Pharmacy, King Khalid University, Abha, Saudi Arabia; 5 Department of General Science, Ibn Sina National College for Medical Studies, Jeddah, Saudi Arabia; 6 Department of Pharmacy Practice, College of Pharmacy, AlMaarefa University, Riyadh, Saudi Arabia; National Institute of Biologicals (NIB), Ministry of Health & Family Welfare, Government of India, INDIA

## Abstract

Kyasanur Forest Disease Virus (KFDV) poses a significant public health threat due to the limited efficacy of existing vaccines, necessitating the development of effective antiviral therapeutics. The nonstructural protein 5 (NS5), essential for viral RNA synthesis and methylation, serves as a promising drug target. This study employs computational approaches to identify and evaluate potential NS5 inhibitors that may contribute to the development of antiviral compounds against KFDV. The 3D structure of NS5 was predicted using Robetta, SwissModel, and I-TASSER, with the Robetta model (ERRAT score: 96.40) selected for energy minimization. The globally minimized structure, obtained at 49.58 ns, had a potential energy of −416966.82 kcal/mol and was used for further studies. Active site residues were identified using template-based and structure-based methods (COACH-D, CASTp, PrankWeb) and were located within polymerase motif (A-G) of NS5 protein (residues 273−903 aa), which are essential for polymerase function, RNA synthesis, and viral replication. A total of 1523 compounds were identified using de novo, template-based design, pharmacophore modeling, and ligand screening. Virtual screening with PyRx 0.8 yielded 34 promising compounds, of which 11 were selected based on molecular docking (AutoDock 4.0) with binding energies of −8.86 kcal/mol (FDA-approved dasabuvir -L1), −8.28 kcal/mol (CNPO331352.1-L2), −7.94 kcal/mol (ZINC00103114410- L3), and −7.61 kcal/mol (CNPO202263.1-L4). MD simulations in triplicates under physiological conditions confirmed stability. with MM-GBSA binding free energy values of −52.28 ± 2.91 kcal/mol (NS5-Dasabuvur L1complex), −46.82 ± 4.31 kcal/mol (NS5-L2 complex), −50.72 ± 6.36 kcal/mol (NS5-L3 complex), and −57.03 ± 4.31 kcal/mol (NS5-L4 complex). The computational analysis suggests that compounds L2 and L4 have strong binding affinities comparable to dasabuvir (L1), indicating their potential as inhibitors of the KFDV NS5 protein. Further validation through in vitro assays would complement these in silico findings. These results provide a foundation for future drug development against KFDV, emphasizing the need for continued exploration of antiviral therapeutics.

## 1. Introduction

The Kyasanur Forest Disease Virus (KFDV), belonging to the Flaviviridae family, was discovered in 1957 as the etiological agent of Kyasanur Forest Disease (KFD) in Shivamogga, Karnataka, India [1–3]. KFDV mainly target black-faced langurs (*Presbytis entellus*) and red-faced bonnet monkeys (*Macaca radiata*), transmitted by infected tick bites, particularly during their nymphal stage, with the ability to cause lifelong infection [3,4]. KFDV, a zoonotic disease also called “monkey fever,” is transmitted through ticks to a range of species, such as rats, shrews, and birds [5]. Adult ticks exhibit a preference for bigger animals when it comes to reproduction. They serve as primary hosts and reservoirs without showing any symptoms. Monkeys, on the other hand, act as secondary hosts, while humans are considered dead-end hosts. Every year, there are 400−500 newly documented KFD cases in humans, and there is a fatality rate ranging from three to five percent [3,6]. The US National Institute of Allergy and Infectious Diseases (NIAID) categorizes KFDV as a priority pathogen of category C because of its high level of pathogenicity and the absence of approved vaccines and treatments [5,7]. KFDV is a category IV pathogen based on risk assessment because of its unexpected epidemiological and ecological traits. According to the 2017 International Classification of Diseases-10 (ICD-10), KFD is categorized as A98.2, among other haemorrhagic fever viruses that are not classified elsewhere [8].

Standard epidemiological investigations systematically monitor and manage epidemics in Shivamogga, Karnataka, usually occurring between November and June [9]. In the last 15 years, there have been outbreaks of a disease that spread to places outside its typical occurrence. Notably, there were many instances in Chamarajanagar, Karnataka, and the nearby Wayanad and Malappuram, Kerala, from 2012 to 2014 [5,6,10]. Similarly, instances were documented in the Goa (North Goa) and Maharashtra (Sindhudurg areas) in 2015 and 2016. Notably, a virus closely related to KFDV, known as Alkhurma hemorrhagic fever virus (AHFV), has been identified in Saudi Arabia and Egypt. AHFV shares significant genetic similarities with KFDV and causes similar hemorrhagic fever symptoms [2,11,12]. A case of KFD was also recently identified in China, being the first instance of KFDV occurring outside of India [13]. The increasing areas where the disease is often found highlight the necessity for novel preventative measures and therapies, which in turn require a more thorough understanding of its causes, environmental factors, and patterns of occurrence [14].

KFDV is a virus that has a spherical shape and is surrounded by a lipid bilayer. It has a diameter of around 45 nm, and its genetic material is made up of an RNA molecule, positive-sense and single-stranded, that consists of 10,774 nucleotides [15]. The polyprotein consists of 3,146 amino acids and consists of three proteins (structural): Precursor Membrane (prM), Capsid (C), and envelope protein (E), as well as seven proteins (non-structural) - NS3, NS2A, NS1, NS2B, NS4B, NS4A, and NS5 [16]. NS5, the largest and highly conserved among non-structural proteins in the Flaviviridae family [17], is composed of two main domains: the methyltransferase (MTase) domain at N-terminal and the RNA-dependent RNA polymerase (RdRp) domain at C-terminal [18].

The RdRp domain catalyzes viral RNA replication by binding to the viral RNA template, synthesizing complementary RNA strands, and producing sufficient genomic RNA for packaging into new virus particles [19–21]. The ~ 260-residue MTase domain of NS5 methylates the 5’ cap structure of viral RNA post-RdRp domain RNA synthesis, incorporating guanylyl transferase (GTase) and guanine N7 MTase and ribose 2′-O MTase activities. Cap methylation stabilizes viral mRNA, enhancing translation efficiency and enabling immune evasion by mimicking host mRNA [22–26]. NS5 plays a crucial role in assembling the viral replication complex, interacting with NS3 [27,28], NS4A, NS4B [29], and promoter stem-loop [30,31] to form a functional complex on intracellular membranes like the endoplasmic reticulum (ER). This complex facilitates efficient viral RNA replication, protecting viral RNA from cellular nucleases [19], and modulates host factors to support viral replication, inhibit interferon (IFN) production [17,32,33], and inhibit the innate immune response [34,35]. NS5 also manipulates host cell signaling to favor viral replication, contributing to flavivirus fitness and pathogenicity by evading host immune surveillance. Understanding NS5’s molecular mechanisms informs antiviral strategies against tick-borne flavivirus infections [27,36–38].

The initial formalin-inactivated whole virus vaccine approach to KFDV prevention showed limited efficacy [15,39], raising uncertainties about its future and leaving public health responses vulnerable. The rising KFDV incidence and growing endemic areas in the country have raised worries about the public region healthcare infrastructure [40]. Given the ineffective current vaccination strategy and lack of antiviral treatments, exploring novel therapeutic approaches for KFDV is crucial. To achieve a comprehensive understanding of the virus, its vectors, pathophysiology, and epidemiology, a collaborative effort across many disciplines is necessary. Given the urgent need for effective antiviral therapeutics against Kyasanur Forest Disease Virus (KFDV), this study focuses on the NS5 protein, a crucial enzyme in viral replication and transcription. By employing computational drug discovery strategies, we aim to identify novel inhibitors that can potentially disrupt NS5 function. This work not only contributes to the growing repository of in silico-driven antiviral research but also serves as a foundation for future experimental validation and structure-based drug design efforts.

## 2. Methodology

**Fig 1:**
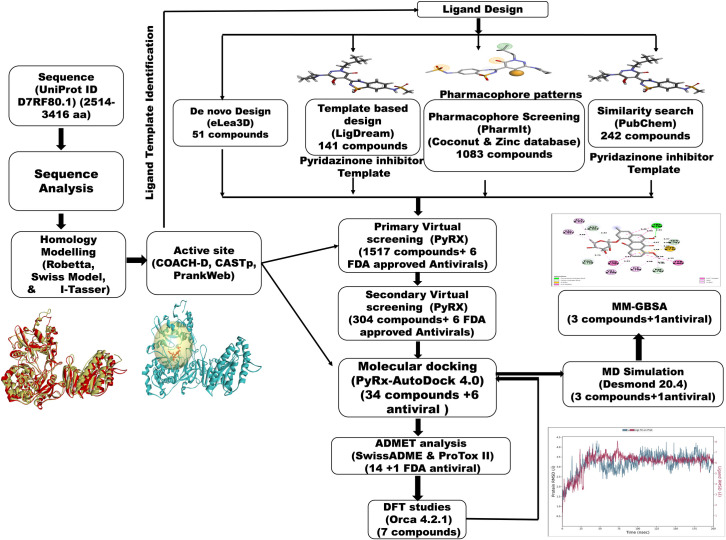
Methodology schema employed in the study for predicting inhibitors for the NS5 protein of KFDV.

A comprehensive computational workflow represented in Fig 1 was followed to identify and characterize potential inhibitors of the NS5 protein of KFDV. The study involved 3D structure modeling and active site prediction, followed by ligand design using de novo, template-based, pharmacophore, and ligand-based approaches. Virtual screening and molecular docking were conducted to identify top candidates, which were then evaluated through ADMET profiling, DFT-based optimization, redocking, molecular dynamics (MD) simulations, and MM-GBSA binding free energy calculations.

### 2.1. Sequence analysis

The amino acid sequence of the non-structural protein 5 (NS5) of Kyasanur Forest Disease Virus (KFDV) was retrieved in FASTA format from the UniProt database (https://www.uniprot.org/) [41]. Physicochemical properties of the NS5 protein, including molecular weight, theoretical isoelectric point (pI), instability index, aliphatic index, and grand average of hydropathicity (GRAVY), were calculated using the ProtParam tool available on the ExPASy server (https://web.expasy.org/protparam/) [42]. To identify homologous proteins with known 3D structures, a BLASTp (https://blast.ncbi.nlm.nih.gov/Blast.cgi) search was performed against the Protein Data Bank (PDB) using the NS5 sequence as a query [43]. The top five sequences with the lowest e-values and highest percentage similarities were selected. Multiple sequence alignment (MSA) was performed using Clustal Omega (https://www.ebi.ac.uk/Tools/msa/clustalo/) [44] to analyze conserved regions, which are often critical for functional or structural integrity. The secondary structure elements were predicted using SOPMA (Self-Optimized Prediction Method with Alignment) (https://npsa-prabi.ibcp.fr/cgi-bin/npsa_automat.pl?page=/NPSA/npsa_sopma_f.html) [45]. Additionally, the transmembrane topology of the NS5 protein was evaluated using TMHMM v2.0 (https://services.healthtech.dtu.dk/services/TMHMM-2.0/) [46], which employs a hidden Markov model to predict the presence and location of transmembrane helices.

### 2.2. 3D structure determination

Since the crystal structure of KFDV NS5 was not available, three widely accepted web-based homology modeling platform like I-TASSER [47], Swiss-Model [49], and Robetta [48] were used for comparative modeling. I-Tasser employs an Iterative Threading Assembly simulation to detect threading templates, simulate the assembly of structures, choose and enhance models, and annotate functions depending on the structure [47]. The Robetta server employs domain based prediction method to generate 3D structural models [48]. The Swiss Model is a web-based application that integrates protein structure homology modeling. It consists of four steps: identifying structural templates, aligning the target sequence with the template structures, creating the model, and evaluating the model’s quality [49]. All three generated models were compared for structural quality and accuracy, and the best model based on evaluation metrics was chosen for further study.

### 2.3. Validation of 3D structure

To validate the stereochemical quality and reliability of the predicted 3D model of the NS5 protein, multiple validation tools were employed: PROCHECK [50] was used to assess the dihedral angles and to generate the Ramachandran plot for visualizing backbone conformations. ERRAT [51] evaluated the non-bonded atomic interactions in the model. QMEAN [52] and ProSA-web [53] were employed for energy profiling and comparison against known PDB structures to assess global and local model quality. Verify3D [54] was used to check the compatibility of the 3D structure with its amino acid sequence (1D profile). These tools ensured that the selected model was suitable for docking and virtual screening studies.

### 2.4. Least potential energy confirmation by MD simulation of NS5 protein

To obtain the lowest potential energy (PE) in kcal/mol conformation of the modelled structure of NS5 protein, MD simulation was performed for 200 ns using the OPLS force field by Desmond 2022−4 software. The protein structure was kept at the center of the cubic box of 12Å and solvated using the SPC model. The system was neutralized by 4Cl^-^ counter ions at physiological conditions (pH 7.4 and temperature 310K).

### 2.5. Binding site prediction

To identify potential ligand-binding pockets in the three-dimensional structure of the energy-minimized NS5 protein model, we employed three distinct and complementary binding site prediction servers: COACH-D, CASTp, and PrankWeb. Each tool was selected for its specific algorithmic strengths and prediction approaches. The COACH D (https://yanglab.nankai.edu.cn/COACH-D/) server predicts binding sites based on an algorithm that forecasts the locations where ligands bind sites by consulting the Bio Lip database. It achieves this by analyzing and comparing particular substructures involved in binding and sequence profiles. The binding sites identified by COACH-D are subsequently prioritized according to their confidence level, referred to as the C-level [55]. The topological and geometric properties of the NS1 protein were analyzed using the Computed Atlas of Surface Topography of Proteins (CASTp) server (https://sts.bioe.uic.edu/castp/). This tool identifies, delineates, and measures the pockets and cavities within protein structures. The CASTp operates on principles from computational geometry, specifically using alpha shape and pocket algorithms. It employs the molecular surface model (Connolly’s surface) and the solvent-accessible surface model (Richards’ surface) to quantify the area and volume of each pocket and void in the protein [56]. The active site prediction was also performed using the PrankWeb server (https://prankweb.cz/), which utilizes a machine learning approach based on structural and evolutionary features. The target protein structure was uploaded in PDB format for analysis. The server identified potential ligand-binding sites by analyzing spatial and conservation patterns. Predicted binding pockets were ranked based on their probability scores, with the highest-ranked site selected for further analysis [57].

### 2.6. Ligand design

In computational drug design, ligand design is essential to finding small compounds that inhibit a protein. The structure-based and ligand-based techniques were used for ligand design in this investigation. Ligand design was performed using a hybrid approach combining de novo drug design, template-based design, pharmacophore modeling, and ligand-based virtual screening strategies to generate a diverse ligand library targeting the predicted NS5 binding site.

#### 2.6.1. De-novo design.

A drug aimed at the NS5 protein’s active site was designed utilizing the LEA3D server (http://www.lea3d.org/) and PLANTS docking tool. Drug compounds were created using Lipinski’s Rule of Five. A genetic algorithm with an initial population of FDA-approved pharmaceuticals and a maximum population size of 51 molecules was used. Drug designing by de novo method involved in creating new ligands by computational approach targeting the active site of the protein. Despite challenges related to synthetic accessibility, de novo drug design offers a cost-effective means of developing drug candidates, potentially yielding novel compounds with enhanced biological activity [58].

#### 2.6.2. Template-based design.

The LigDream tool [59] (https://www.playmolecule.org/LigDream/) of the playmolecules server was used to design new ligands by artificial neural network based on the template pyridazinone inhibitor (PubChem ID: 135566439). LigDream leverages neural network models trained on vast chemical space to design new derivatives by either replacing atoms/groups (replace mode) or by growing new fragments (grow mode) on the scaffold. Both modes were explored to maximize the diversity of generated compounds [60].

#### 2.6.3. Pharmacophore screening.

To explore ligand databases using the 3D structure of the ligand-protein complex, the Pharmit server (https://pharmit.csb.pitt.edu/) [61] was utilized for ligand-based and structure-based pharmacophore modeling. By docking known active drugs into the vacant binding pocket of computationally created ligand-target complexes, structure-based pharmacophore models may be constructed. The Pharmit server utilized pharmacophore patterns obtained from protein-ligand interactions in Discovery Studio version 2021(BIOVIA, San Diego, CA, USA) [62] to filter ligands from COCONUT [63] and zinc [64] databases. The pharmacophore model was constructed using the Pharmit server and used characteristics derived from the NS5 protein and its interaction with the pyridazinone inhibitor (PubChem ID: 135566439). The model was used to search the COCONUT database, which contains conformers of 87, 39,148, and the zinc database, which has 12, 22, 76,899 conformers. Pharmit improved the positions of effective compounds, which were further tested with the target protein to find leading compounds with the highest docking scores [61].

#### 2.6.4. Ligand-based screening.

The PubChem database stores information about chemicals and their biological activities. It was established in 2004 as a component of the Molecular Libraries Roadmap Initiatives at the National Institutes of Health (NIH). The databases that make up this system include Substance, Compound, and Bioassay. The ligand discovered by the COACH-D server, pyridazinone inhibitor (PubChem ID: 135566439) was utilized as a template to search for comparable ligands in the PubChem database using Lipinski’s algorithm [65]. The PubChem database was curated using a number of criteria, including Molecular weight (MW) between 160 and 500; hydrogen bond (HB) donors and acceptors not exceeding 5 and 10, respectively; and a number of bonds (rotatable) ranging from 1 to 17. The atom number does not exceed 40; the hydrophobicity (logP) should be equal to or less than 5.

### 2.7. Virtual screening (VS)

The virtual screening pipeline integrated all designed ligands from sections 2.6.1 to 2.6.4 along with known FDA-approved dengue NS5 inhibitors. The workflow used PyRx 0.8, a graphical interface for AutoDock Vina for virtual screening and AutoDock 4.0 for molecular docking. All ligands designed by different approaches of ligand design were combined in one SDF file and were used for virtual screening. The ligands were converted into AutoDock PDBQT format from this SDF file within PyRx 0.8 [66] using Open Babel [67]. The PyRx AutoDock Vina [68] module was used for the primary and secondary virtual screening with an ‘exhaustiveness’ value of 100. To define the search space, a grid box was created at X 0.5437, Y 12.7608, Z −11.9581, with grid point of 30 on XYZ dimensions for globally minimized NS5 protein. Virtual screening was performed by considering all the ligands designed by different ligand design methods plus FDA-approved dengue virus NS5 protein antivirals as a control with against the globally minimized NS5 protein. PyRx 0.8, which utilizes AutoDock Vina, was chosen for its rapid docking performance including streamlined parameters and much faster docking performance and effectiveness with ligands containing multiple rotatable bonds [69]. Top 20% of the compounds with highest binding affinities were further taken to tertiary screening using molecular docking approach by AutoDock 4.0 to refine binding poses and assess binding energies with greater configurational control. This combination of Vina for high-throughput screening and AD4 for detailed validation aligns with established protocols in structure-based drug design [69].

Molecular docking plays a crucial role in the field of drug design (computational) by evaluating the interactions between the ligand and an active site of NS5 protein. This includes analyzing the affinity of binding, free energy, and the stability of the ligand-protein complex. Ligands after secondary screening were docked with NS5 using AutoDock 4.0 implemented in PyRx 0.8 [70,71]. Receptors were added with hydrogen atoms (polar) and charges (Kollman) within a docking a grid box was created at X 0.5437, Y 12.7608, Z −11.9581, with grid point of 30 on XYZ dimensions. The Auto Grid 4.0 uses default parameters for the calculation of desolvation, affinity, and electrostatic maps. The Lamarckian Genetic Algorithm (LGA) [72] examined the arrangements of ligands in 50 runs, with a maximum of 25,000,000 assessments of energy, a population size of 250, and 27,000 generations in each iteration. The parameters like crossover rate of 0.8 and mutation rate of 0.02 were set, utilizing a two-mode crossover. Docking results assessed ligand binding’s effect on NS5 affinity, from BIOVIA Discovery Studio 2021 [73].

### 2.8. ADMET studies

In order to have therapeutic benefits, a drug candidate must reach the target location in the body at the most effective concentrations. Anticipating pharmacological and physicochemical factors is essential as it offers valuable information about whether a molecule can effectively reach and sustain enough concentrations at the active site to initiate the intended biological reaction. Before proceeding to clinical trials and in the pursuit of developing improved lead compounds, it is essential to do in-silico toxicity studies. The use of in silico toxicity evaluations has become more common because of their high level of accuracy, fast processing speed, and easy accessibility. These evaluations allow for a thorough examination of both synthetic and natural substances. Drug development requires an assessment of drug-likeness and ADMET characteristics. The Swiss ADME server (http://www.swissadme.ch/) predicted ADME using each molecule’s canonical SMILES [74]. The ProTox II web server (https://tox-new.charite.de/protox_II/) [75] predicted oral toxicity, carcinogenicity, hepatotoxicity, cytotoxicity, and mutagenicity. Drugs are classified into toxicity classes of 1–6 by ProTox II, with class 1 indicating significant danger (LD50 < 5) and class 6 indicating non-toxicity (LD50 > 5000). The top 14 PyRx compounds with the best binding energies were tested in ADMET with FDA-approved NS5 protein antivirals.

### 2.9. DFT calculations

Geometry optimization is a quantum chemical method used to improve initial geometric approximations in order to attain the utmost accuracy. Geometric optimization of the selected ligands was carried out using density functional theory (DFT), involving comprehensive quantum mechanical wave function analysis. Becke’s three-parameter hybrid density functional theory (B3LYP) with ORCA version 4.2.1 facilitated optimization and vibration frequency calculations [76], integrating various functional types. AVOGADRO version 1.2.0 generated ORCA input files to support these computations [77]. Frequency calculations established thermodynamic properties and verified each optimization step for energy minimization, ensuring structural stability.

The Frontier Molecular Orbital (FMO) hypothesis is critical in the field of organic chemistry as it provides valuable insights into the structure and reactivity of molecules. The molecule’s characteristics can be determined by analyzing the energy difference between the highest occupied molecular orbital (HOMO) and the lowest unoccupied molecular orbital (LUMO). The electron transfer from the HOMO to the LUMO called the HOMO-LUMO energy gap, plays a critical role in organic chemistry. The difference between the HOMO and the LUMO serves as an indicator of reactivity and stability. Electrons in the HOMO participate in nucleophilic reactions, whereas electrons in the LUMO are participated in electrophilic processes. Molecules possessing a limited HOMO-LUMO gap exhibit heightened reactivity and reduced stability, rendering them “soft.” Conversely, molecules with a substantial gap display diminished reactivity, enhanced stability, and decreased bioactivity. It is essential for understanding the photochemistry, potency, and robustness of transition metal compounds. The energy values of HOMO and LUMO were calculated and displayed by Chemcraft 1.8 to evaluate the vulnerability of atoms to nucleophilic and electrophilic interactions [78]. The HOMO-LUMO gap, which represents the difference in energy between orbitals, was calculated using a particular equation, yielding valuable information on the chemical properties and reactivity of the molecule.

### 2.10. Redocking

The redocking methodology involved preparing the optimized ligand structures obtained from the geometric optimization process for accurate binding evaluation. The optimized ligands were imported into the docking software. A similar methodology was followed as described in the molecular docking section. The binding energy of the ligands with the NS5 protein was compared for the ligands before and after geometric optimization.

### 2.11. MD simulation studies

MD simulations using Desmond 2022.4 (Desmond Molecular Dynamics System, version 2022.4, D. E. Shaw Research, 2016, New York) were conducted on the NS5 protein and its complexes with L2, L3, L4, and dasabuvir (L1) in triplicates. Simulations ran at pH 7.4 and temperature of 37°C reflecting the physiological conditions with the force field (OPLS-2005) [79] and an explicit solvent model (SPC water molecules) in a 12 Å x 12 Å x 12 Å periodic boundary box. The randomized velocity approach in Desmond was employed to initialize the atomic velocities at the start of the molecular dynamics (MD) simulation. Cl^−^ ions were added for 0.15 M charge neutralization, with NaCl solutions simulating at physiological conditions (pH:7.4, Temperature:310K). Initial equilibration lasted 10 ns under an NVT ensemble to stabilize protein-ligand complexes, followed by an NPT ensemble of 12 ns of equilibration and minimization run. The ensemble (NPT) employed the Nose-Hoover chain coupling scheme [80] at 1 bar pressure, variable temperature, and a relaxation time of 1.0 ps. Pressure regulation used the Martyna-Tuckerman-Klein chain coupling scheme with a 2 ps relaxation time and a 2 fs time step. The Ewald method with a 9 Å Coulomb interaction radius managed long-range electrostatic interactions using the particle mesh. The RESPA integrator calculated bonded forces with a 2 fs time step. A 200 ns production run assessed MD simulation stability, monitoring RMSD and RMSF parameters [81].

### 2.12. Binding free energy analysis of protein-ligand complex

The MM-GBSA method calculated free energies (binding) for L2, L3, L4, and dasabuvir (L1) ligand-protein complexes. Python script (thermal mmgbsa.py) was used under Prime MM-GBSA to analyze the trajectory of the last 50 frames with a sampling of 5 steps, yielding free energies (binding) in kcal/mol. This approach assessed covalent bonds, Coulombic interactions, van der Waals forces, hydrogen bonds, lipophilic interactions, self-contact energy, and protein solvation to evaluate overall ligand-protein interaction stability [82].

## 3. Results

### 3.1. Sequence analysis

The protein sequence of NS5 protein (2514–3416 of 903aa) with ID D7RF80.1 was retrieved from UniProt. BLASTp analysis against the PDB database using default parameters identified homologous sequences, detailed in Table 1. Multiple sequence alignment (MSA) with Tick-borne encephalitis virus, Yellow fever virus, Dengue virus-2, Zika virus, Ntaya virus, Japanese Encephalitis virus, and Kunjin virus (PDB ID – 7D6N, 6QSN, 5ZQK, 5M2X, 7ZIU, 4K6M, and 2HFZ, respectively) was performed using Clustal Omega, revealing significant conservation among flaviviruses across most regions are presented in supporting information as S1 Fig.

**Table 1:** BLASTp results of the NS5 protein showing homologous sequence.

Sr.No.	PDB ID	Organism	Max Score	e-Value	Identity %	Query cover%
01	7D6N	Tick-borne encephalitis virus (strain HYPR)	1148	0	86.12	69
02	6QSN	Yellow Fever Virus	1123	0	59.98	99
03	5ZQK	Dengue virus 2	1065	0	58.65	98
04	5M2X	Zika virus	1033	0	58.98	97
05	7ZIU	Ntaya virus	832	0	63.88	67
06	4K6M	Japanese encephalitis virus	1076	0	63.58	67
05	2HFZ	Kunjin Virus	835	0	63.81	68

The physicochemical characteristics of the NS5 protein were evaluated utilizing the ProtParam tool, summarized in Table 2. The protein’s theoretical isoelectric point (pI), calculated at 8.03, indicates its basic nature, where it achieves electrical neutrality and stability in an electro-focusing system [83]. Additionally, the protein’s aliphatic index (AI) of 78.39 suggests stability across various temperatures due to the presence of a large number of amino acids (aliphatic side chain) such as isoleucine, alanine, leucine, and valine [84].

**Table 2:** NS5 protein physicochemical characteristics.

Features	Remark
Protein	NS5 protein
Accession Number	D7RF80.1|POLG_KFDV:2514–3416
Length of sequence	903 aa
pI (Theoretical)	8.03
Molecular mass	102.86 kDa
Index (Aliphatic)	78.39
GRAVY	−0.484
Index (Instability)(II)	33.13 Stable

The NS5 protein’s instability index (II) was determined to be 33.13, indicating stability in solutions containing specific dipeptides [85], as it falls below the 40 threshold. The Grand Average of Hydropathicity (GRAVY) value calculated was −0.484, indicating the protein’s hydrophilic nature and favorable affinity towards water molecules. This is particularly relevant given NS5’s role in viral protein synthesis within the host cell’s endoplasmic reticulum and its hydrophilic active sites [86–88]. The NS5 protein’s secondary structure was evaluated using SOPMA (Structure and Function of Biomolecules: Parallelized In-Silico Molecular Analysis), and the results are summarized in Table 3. The analysis revealed that the protein consists primarily of alpha helices, comprising 42.52% of its amino acid composition. Random coils make up 36.66% of the 2D structure, while extended strands and beta turns constitute 14.84% and 5.98%, respectively. This distribution suggests NS5 can be classified as a mixed-class protein. Furthermore, TMHMM 2.0 predicted the transmembrane region of NS5, classifying it as an extracellular protein, with all amino acids located in this region. The detailed findings are summarized in the supporting information in S1 Table.

**Table 3:** 2D structure analysis of NS5 protein by SOPMA.

Alpha helix(Hh)	384 is 42.52%
Extended strand (Ee)	134 is 14.84%
Pi helix (Ii)	0 is 0.00%
Bend region (Ss)	0 is 0.00%
Random coil(Cc)	331 is 36.66%
Beta turn(Tt)	54 is 5.98%
Beta bridge(Bb)	0 is 0.00%
3_10_ helix(Gg)	0 is 0.00%

### 3.2. 3D structure determination and validation

Since the experimentally determined 3D structure of the NS5 protein of KFDV is not available in the Protein Data Bank (PDB), a homology modeling approach was employed to predict its tertiary structure. The NS5 protein’s 3D structure was determined using I-Tasser, Robetta, and Swiss Model servers. The model generated by Robetta has a 0.92 confidence score using the PD algorithm. I-Tasser produced five models, among which the one with the highest c-score of 2, 0.99 ± 0.04TM-score, and 3.9 ± 2.6Å RMSD were selected. Swiss Model used the template from Japanese encephalitis virus (PDB ID: 4K6M) with a GMQE score of 0.80. To validate these models, a range of assessment methodologies for structure quality were utilized and summarized in Table 4. The stereochemical analysis using PROCHECK revealed different regions within the models, including the most favoured, additional allowed, generously allowed, and disallowed regions. Approximately 93.3% of amino acids in the Robetta model were in the favoured region, indicating higher stereochemical quality compared to I-Tasser and Swiss Models. Such high Ramachandran plot statistics reflect accurate backbone conformations, an important criterion for model reliability. Verify3D analysis showed that over 90% of residues in all models had a 3D-1D score of ≥ 0.2 on average, passing the validation criteria. Robetta received a higher ERRAT score (96.629) compared to I-Tasser (91.592) and Swiss Model (92.89), indicating better tertiary structure quality. This ERRAT score evaluates non-bonded atom–atom interactions and is a strong indicator of overall model integrity. ProSA analysis confirmed Z-scores of the models lied within the range of PDB native structures, further validating the quality of the model. Collectively, these validation results confirmed the Robetta-predicted model as the most reliable candidate for downstream computational studies, including stability studies, binding site prediction, and ligand docking.

**Table 4:** Generated 3D model’s quality assessment.

Validation method	Robetta Model	I-Tasser Model	Swiss Model	Refined Robetta Model
ERRAT score	96.629	91.592	92.89	90.212
Procheck				
Most favoured region	93.3	82.6	92.3	83.3
Additionally allowed regions	6.6	15.2	7.2	15.3
Generously allowed region	0	1.1	0.1	1.3
Disallowed region	0.1	0.8	0.4	0.1
ProSA Web score	−12.28	−11.86	−12.27	−11.45
Verify 3D	92.69	91.36	90.19	93.36
QMean Disco Global	0.82 ± 0.05	0.78 ± 0.05	0.81 ± 0.05	0.68 ± 0.05
QmeanZscore	0.21	−4.21	−1.44	−3.94
Qmean all atom	−0.34	−0.30	−0.36	−1.30
Qmean torsion	0.33	−3.37	−1.18	−3.29
Qmean Solvation	0.14	−1.55	−0.16	−0.98
Qmean Cβ	−1.01	−1.04	−1.11	−1.30

**Fig 2:**
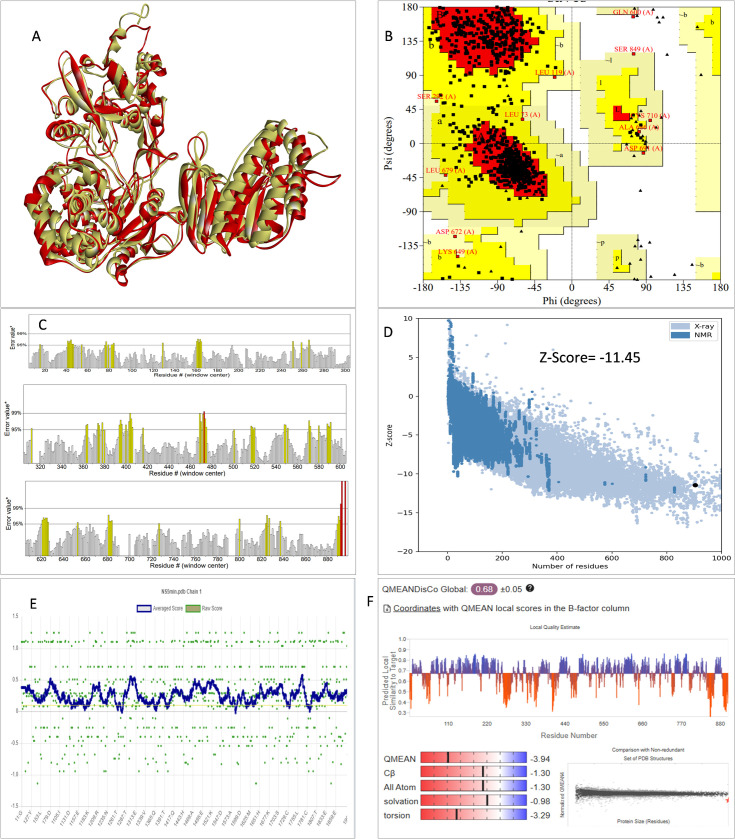
A: Superimposition of 3D structure of Robetta model (yellow colour) and minimized model (Red colour) and Validation-NS5 protein; B: Ramachandran plot; C: ERRAT quality factor; D: ProSA Web score; E: Verify3D score; and F: Qmean score.

The stability of the NS5 protein modelled structure from Robetta was validated by an MD run of 200 ns, and it was found to be highly stable. The lowest potential energy conformation for the NS5 protein was found to be −416,966.82 kcal/mol at 495852.0^th^ ps (49.58 ns). The lowest PE conformations were extracted and used further for active site determination and docking studies. This selection ensures that downstream analyses are based on a realistic, dynamically stable protein conformation that better reflects in vivo conditions. Fig 2 illustrates the diagrams of 3D structure and its validation of the selected Robetta model. To assess the structural deviation between the Robetta-predicted model (yellow colour) and the potential energy-minimized model (red colour) in Fig 2A, we performed structural superimposition using Discovery Studio. The alignment yielded an overlay score of 0.7123, indicating a moderate degree of structural similarity. Such comparative structural assessment is critical to ensure that the refined model remains functionally relevant. Furthermore, the C-alpha root mean square deviation (RMSD) was calculated as 2.882 Å, suggesting that while energy minimization induced some conformational adjustments, the overall fold and topology of the protein remained conserved. These findings confirm that the energy-minimized model retains the structural integrity of the initial Robetta model while potentially achieving a more stable conformation. This validation supports the model’s suitability for virtual screening, ligand docking, and simulation studies by confirming both structural stability and biological plausibility. This refined structure was subsequently utilized for molecular docking and molecular dynamics (MD) simulations to ensure biologically relevant interactions and stability assessments.

### 3.3. Binding site prediction

**Fig 3:**
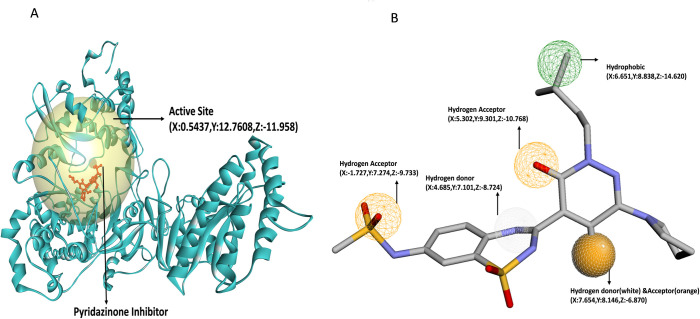
(A) Active site (transparent yellow sphere) of NS5 protein with Pyridazinone inhibitor (Red color, Ball & Stick model) binding; (B) characteristics of pharmacophore model: orange globe sphere (full & line pattern)-HB-A, white dotted line pattern on orange globe sphere and white line pattern globe sphere- HB-D, & green line pattern globe sphere- HP site.

Following the validation of the NS5 protein structure through molecular dynamics simulation and structural superimposition, the energy-minimized and structurally stable model was employed for active site prediction using multiple in silico tools. Binding site residues were identified using a consensus-based approach involving three well-established web servers: COACH-D, CASTp, and PrankWeb, each utilizing different algorithms to enhance prediction reliability. Table 5 shows the active site of the NS5 protein determined by the COACH-D, CASTp, and PrankWeb server, which is important for drug design. The active site determined from COACH-D and PrankWeb was selected based on the confidence score. The confidence score or c-score of 0.15 and a c-score of 8.74 were selected from COACH-D [89] and PrankWeb, respectively. Whereas pocket 1 was selected as an active site, as determined by the CASTp server. Table 5 shows the top five predicted sites’ binding residues, and grid box dimensions of COACH-D and PrankWeb, the pocket area and volume with amino acid residues for the CASTp server. The predicted binding pockets from each method were compared, and residues common to at least two rank-1 predictions were considered as the final active site residues. These residues were used to define the docking grid box for virtual screening. Grid box dimensions were calculated using Discovery Studio based on the spatial coordinates of the selected active site. The final grid setup and residue composition are presented in Table 5, and the graphical representation is shown in Fig 3. The COACH-D used pyridazinone inhibitor (PubChem ID: 135566439) as a preliminary ligand template for the first binding site, having a c-score of 0.15, enabling pharmacophore pattern generation and ligand screening as represented in Fig 3.

**Table 5:** Information of NS5 protein’s Active site by COACH-D, CASTp and PrankWeb.

Predicted binding site	Score/ c-value	Active site grid box in Å	Amino acid Residue
**COACH-D**			
1	0.15	X:4.163 Y:12.621 Z:-12.744	Leu513, Leu516, Asn612, Ser662, Gly663, Asp664, Asp665, Phe709, Cys710, ser711, His712, Met762, Thr794, Thr795, Trp796, Ser797, Ile798, Ala800, Trp804
2	0.08	X:-16.391 Y:14.626 Z:23.25	Phe768, His769, Arg770, Arg771, Asp772, Arg774, Pro838, Tyr839, Leu840, Pro841, Asp845, Arg857, Asp888, Ile903
3	0.04	X:9.50 Y:2.764 Z:-4.698	Lys359, Lys458, Arg473, Asp535, Thr536, Gly538, Trp539, Asp540, Ser603, Thr608, Asn612, Asp664, Lys690
4	0.03	X:6.747 Y:17.563 Z:-4.931	Ala402, Lys403, Arg405, Ser406, Asn407, Ala409, Asn418, Met456, Lys458, Ile475, Tyr477, Arg483, Asn494, Glu509, Gly510, Ser512, Ser603, Gly604, Gln605, Val606, Tyr609, Ser662, Gly663, Asp664, Asp665, Cys710, Ser711, Arg730, Leu755, Ala758, Tyr759, Met762, Leu765, Val789, Val813, Arg816
5	0.01	X:6.157 Y:10.888 Z:8.523	Thr315, Ala412, Asn454, Met456, Gly457, Lys458, Arg459, Ile475, Tyr477
**CASTp**			
**Pocket No.**	**Area** **(Å**^**2**^)	**Volume** **(Å**^**3**^)	**Amino acid Residue**
1	4117.96	6174.47	Glu76, Val77, Met98, Gly99, Val100, Lys101, Tyr103, Val112, Pro113, Arg114, Leu115, Trp121, Asn122, Leu123, Ile124, Lys125, Phe127, Thr128, Met130, Ser134, Leu135, Glu136, Ala137, His138, Arg174, Val266, Val267, Pro300, Tyr301, Arg302, Thr303, Trp304, Asn323, Asp338, Arg341, Met342, Met344, Phe350, Gln365, Glu399, Phe400, Ala402, Lys4.3, Val404, Arg405, Asn407, Ala408, Ala409, Gly411, Ala423, Met455, Met457, Lys458, Arg459, Glu460, Lys461, Lys462, Leu463, Glu465, Phe466, Val468, Ala469, Lys470, Gly471, Ser472, Arg473, Ala474, Ile475, Trp476, Tyr477, Leu480, Arg483, Asn494, Glu495, His497, Glu509, Gly510, Thr511, Leu516, Tyr532, Ala533, Asp534, Asp535, Thr536, Ala537, Gly538, Trp539, Asp540, Thr541, Arg542, Val579, Val581, Ile595, Arg597, Asp599, Gln600, Arg601, Gly602, Ser603, Gly604, Gln605, Val606, Thr608, Tyr609, Asn612, Asn616, Val619, Gln620, Arg623, Ser662, Gly663, Asp664, Asn665, Cys666, Val667, Val668, Phe675, Leu682, Asn683, Ala686, Lys687, Val688, Arg689, Lys690, Asp691.Ile692, Gly693, Glu694, Trp695, Glu696, Tyr701, Pro708, Phe709, Cys710, Ser711, Arg730, Asp731, Asp733, Glu734, Gly737, Arg738, Ala739, Val741, Ser742, Pro743, Trp747, Glu751, Thr752, Cys754, Leu755, Ala758, Tyr759, Val789, Pro790, Gln791, Gly792, Arg793, Thr794, Thr795, Trp796, Ser797, Ile798, His799, Ala800, Ser801, Gly802, Met805, Thr806
**PrankWeb**			
**Predicted binding site**	**Score/** **c-value**	**Active site** **grid box in Å**	**Amino acid Residue**
Pocket1	8.74	X:-2.51, Y:14.46, Z:-13.52	Leu513, Cys710, Ser711, His712, Arg730, Glu734, Leu735, Arg738, Tyr759, Met762, Ser766, Tyr767, Thr794, Thr795, Ala800, Trp804
Pocket2	7.58	X:10.61, Y:0.93, Z:5.48	Trp304, Val355, Phe356, Val360, Met455, Arg473, Ala474, Trp476, Asp540, Thr541, Val579, Ile595, Arg597, Gln600
Pocket3	4.7	X:2.65, Y:-7.80, Z:10.61	Leu115, Trp121, Asn122, Ile124, Lys125, Phe126, Val266, Pro300, Arg302, Val353, Phe356, Lys357, Asp361
Pocket4	4.57	X:-12.25, Y:-16.48, Z:14.43	Ser56, Asp79, Gly81, Gly83, Arg84, Gly85, Gly86, Trp87, Ser88, Phe126, Asp146,
Pocket5	4.21	X:12.02, Y:-0.89, Z:-14.17	Tyr532, Ala533, Asp534, Asp535, Thr536, Trp539, Asn616, Val619, Gln620, Arg623, Asp664, Asp665, Cys666, Phe675, Leu682, Lys687, Arg689
**Active site dimension as determined by taking amino acid residues from all three methodologies**			
Active site		X:0.543719, Y:12.760809, Z:- 11.958161	Leu513, Leu516, Asn612, Ser662, Gly663, Asp664, Asp665, Phe709, Cys710, Ser711, His712, Arg730, Glu734, Arg738, Tyr759, Met762, Thr794, Thr795, Trp796, Ser797, Ile798, Ala800, Trp804

### 3.4. Ligand design

To identify potential inhibitors against the KFDV NS5 protein, a combination of structure-based, template-based, pharmacophore-guided, and ligand-based strategies were employed to maximize chemical diversity and biological relevance. Each approach complements the others by exploring different aspects of chemical space and interaction profiles.

#### 3.4.1. De novo drug designing.

This method was used to generate novel small molecules from molecular fragments without relying on existing ligands, allowing the design of structurally unique compounds that may bind the active site. The web server e-LEA3D utilizes molecular fragments for designing new compounds, combining ligand and structure-based evaluations [90,91]. The design criteria include a molecular weight of 100–499, a log P value of 0–5, 1–5 hydrogen bond (HB) donors, 1–10 HB acceptors, and XYZ coordinates of the active site as X:0.543719, Y:12.760809, Z:- 11.958161. The genetic algorithm uses a starting FDA-approved population of drugs, with a maximum of 20 in each of 50 generations. The e-LEA3D server-generated 51 novel compounds, labeled LIG1 to LIG51, using existing fragments from the database. The full list of these chemicals is available in supplemental information S2 Table and was analyzed using PyRx software.

#### 3.4.2. Template-based design.

To retain the biological relevance of known inhibitor scaffolds, template-based design was performed starting from a known pyridazinone scaffold. The LigDream tool of the playmolecules server was used to design 141 new ligands by artificial neural network based on the template pyridazinone inhibitor (PubChem ID: 135566439) [59]. 100 compounds were generated by replace mode, which will replace the existing substituents with random chemically reasonable moieties, and 41 compounds from grow mode, which will add fragments in a combinatorial approach to the scaffold’s chemically accessible position. The approach employs auto-encoders and captioning networks to differently create many drugs (141), starting with pyridazinone inhibitor. The complete list of these compounds can be found in supplementary information S3 and S4 Tables and was subsequently utilized for virtual screening using PyRx software.

#### 3.4.3. Pharmacophoric screening.

This approach was employed to identify common structural features essential for binding based on known inhibitor interactions, thereby guiding ligand selection through feature-based filtering. The NS5 protein structure of KFDV was submitted to the COACH-D server to predict potential ligand binding sites. Based on structural similarity with the Hepatitis C Virus NS5B protein complexed with pyridazinone inhibitor (PDB ID: 3E51) [92–95], a pyridazinone inhibitor was identified and modelled in complex with the KFDV NS5 polymerase domain. This modelled NS5–pyridazinone complex was then submitted to the Pharmit server to generate a structure-based pharmacophore model. This approach allowed the extraction of key pharmacophoric features [96–99] from a predicted ligand–receptor complex, supporting virtual screening in the absence of multiple known inhibitors. In supporting information S5 Table and Fig 3, you can see the detailed pharmacophore characteristics. Ensuring that the screened compounds aligned with the pharmacophore posture required the use of “inclusive” shape constraints with a tolerance level of 1. This widened the search scope. To find compounds that fit Lipinski’s drug-likeness criteria [100], which include a molecular weight between 100 and 499, a maximum of 10 HB acceptors, and a maximum of 5 HB donors, additional filters were used to find compounds with a more “drug-like” profile. These parameters were measured from 0 to +5. Two large datasets were searched using the model: the Zinc database with 13,127,550 molecules and 122,276,899 conformers, and the library of the COCONUT database with conformers of 8,739,148 from molecules of 793,782. Using the pharmacophore pattern obtained from the pyridazinone inhibitor, the search in the Zinc database gave 659 results, whereas the search in the COCONUT database provided 424 hits. Afterward, the binding energies of the chosen hits were computed using Pharmit’s AutoDock Vina scoring tool.

#### 3.4.4. Ligand-based screening.

This method aimed to retrieve chemically similar analogues from public databases using known ligands as reference, thereby leveraging structural similarity for potential activity. We followed Lipinski’s Rule of 5 (molecular weight cutoff: 100–500), a ligand-based screening against PubChem with an 80% Tanimoto threshold, and a ligand from COACH-D (hydrogen bond donors: 1–5, hydrogen bond acceptors: 4–10). We used PyRx software to virtually screen 242 compounds against the NS5 protein. The compounds were downloaded in SDF format.

### 3.5. Virtual screening

Following the identification and preparation of potential ligands using various ligand design methods, including de novo drug designing, template-based design, and pharmacophore-guided screening, the next step involved the virtual screening of these compounds against the KFDV NS5 protein. The virtual screening was carried out in two stages: primary and secondary screening. In the primary screening phase, an extensive library of compounds selected from different drug designing approaches was screened to identify molecules with potential binding affinity towards the target protein. A total compound of 1523 including 6 FDA approved antiviral compounds was screened using PyRx with AutoDock Vina configured to an ‘exhaustiveness’ value of 100 [101]. The ligands were selected based on the lowest binding affinities [63]. Through this screening process, a total of 304 hits were found based on their strong affinity for binding and their specific positioning inside the protein’s binding region. The top-ranking compounds from the primary screening were subjected to secondary screening, using PyRx with AutoDock Vina configured to an ‘exhaustiveness’ value of 100. This two-stage approach ensured the identification of promising drug candidates with improved binding affinity, stability, and favorable pharmacokinetic properties. Through secondary screening, a total of 34 compounds and 6 FD approved antiviral drugs were taken to process of molecular docking using AutoDock 4.0. Table 6 shows the selected 34 compounds with 6 FDA approved antiviral drugs with their binding affinity score. Virtual screening of different ligands generated from different ligand design methods is provided in supplementary information in S6 Table for primary screening and S7 Table for secondary screening.

**Table 6:** Drugs selected after virtual screening by PyRx (AutoDock Vina) software.

Ligand ID	Formula	Binding Energy (kcal/mol)
CNP0202263.1	C_26_H_26_O_9_	−10.1
CNP0144362.6	C_25_H_22_O_10_	−10.0
ZINC000103114410	C_23_H_17_N_3_O_5_	−10.0
CNP0212589.2	C_23_H_22_O_11_	−9.8
LIGR56	C_23_H_29_N_7_O_6_S	−9.8
CNP0133812	C_22_H_20_O_12_	−9.7
CNP0205063.1	C_22_H_20_O_11_	−9.7
CNP0272687.1	C_24_H_20_O_10_	−9.7
CNP0320547	C_23_H_22_O_12_	−9.7
LIGR20	C_23_H_31_N_7_O_5_S	−9.7
LIGR50	C_24_H_30_N_6_O_5_S_2_	−9.7
CNP0206581	C_22_H_22_O_11_	−9.6
CNP0208322.3	C_23_H_22_O_11_	−9.6
CNP0331352.1	C_21_H_18_O_12_	−9.6
LIGR19	C_24_H_29_N_5_O_7_S	−9.6
LIGR1	C_25_H_36_N_6_O_4_S	−9.6
LIGR4	C_21_H_26_N_8_O_6_S	−9.6
LIGR8	C_24_H_32_N_6_O_6_S	−9.6
ZINC000334160896	C_24_H_30_O_9_	−9.6
CNP0117753	C_22_H_24_O_10_	−9.5
CNP0196129	C_24_H_24_O_11_	−9.5
LIGR2	C_25_H_35_N_5_O_6_S	−9.5
ZINC000504372972	C_25_H_25_NO_9_	−9.5
ZINC000514287430	C_26_H_32_N_4_O_5_	−9.5
CNP0123120.1	C_26_H_22_O_10_	−9.4
CNP0124508.2	C_26_H_24_O_10_	−9.4
CNP0282050	C_25_H_26_FN_3_O_4_	−9.4
CNP0356406	C_22_H_22_O_11_	−9.4
CNP0379615.3	C_26_H_27_NO_8_	−9.4
LIGR12	C_25_H_34_N_6_O_5_S	−9.4
LIGR47	C_23_H_34_N_8_O_4_S	−9.4
ZINC000253523417	C_21_H_27_N_5_O_6_	−9.4
136046538	C_20_H_21_N_5_O_4_S_2_	−9.3
CNP0123015	C_24_H_20_O_11_	−9.3
**FDA approved Antivirals**		
Dasabuvir (CID:56640146)	C_26_H_27_N_3_O_5_S	−8.6
Favipiravir (CID:492405)	C_5_H_4_FN_3_O_2_	−6.2
Galidesivir (CID:10445549)	C_11_H_15_N_5_O_3_	−6.6
Remdesivir (CID:121304016)	C_27_H_35_N_6_O_8_P	−8.1
Ribavirin (CID:37542)	C_8_H_12_N_4_O_5_	−6.9
Sofosbuvir (CID:45375808)	C_22_H_29_FN_3_O_9_P	−8.2

### 3.6. Molecular docking

Following the two-stage virtual screening process, which successfully identified 304 hits in the primary screening and 34 promising compounds in the secondary screening, the next step involved molecular docking to further evaluate the binding interactions between the selected ligands and the NS5 protein. Molecular docking is an essential tool for understanding the precise interaction at the atomic level between ligands and proteins [102]. It has a crucial function in the evaluation of pharmaceuticals from databases. Following the virtual screening, 34 compounds and 6 FDA-approved antivirals underwent a docking process using AutoDock 4.2 implemented in PyRx 0.8 was utilized to analyze the binding affinity. This process analyzed the binding affinities of the compounds to the NS5 protein, providing insights into their potential as inhibitors. The top 11 ligands with the highest binding affinities, as detailed in Table 7, were then selected for further evaluation through ADMET (Absorption, Distribution, Metabolism, Excretion, and Toxicity) analysis to assess their pharmacokinetic properties and drug-likeness.

**Table 7:** Molecular docking by AutoDock 4.0 software.

Ligand ID	Formula	Binding Energy (kcal/mol)	
		AutoDock	AutoDock Vina
CNP0331352.1	C_21_H_18_O_12_	−8.28	−9.6
ZINC000103114410	C_23_H_17_N_3_O_5_	−7.94	−10.0
CNP0202263.1	C_26_H_26_O_9_	−7.61	−10.1
ZINC000514287430	C_26_H_32_N_4_O_5_	−7.5	−9.5
136046538	C_20_H_21_N_5_O_4_S_2_	−7.42	−9.3
ZINC000504372972	C_25_H_25_NO_9_	−7.39	−9.5
CNP0272687.1	C_24_H_20_O_10_	−7.35	−9.7
LIGR20	C_23_H_31_N_7_O_5_S	−7.33	−9.7
LIGR12	C_25_H_34_N_6_O_5_S	−7.16	−9.4
LIGR19	C_24_H_29_N_5_O_7_S	−7.02	−9.6
CNP0379615.3	C_26_H_27_NO_8_	−6.71	−9.4
**FDA approved Antivirals**			
Dasabuvir (CID:56640146)	C_26_H_27_N_3_O_5_S	−8.86	−8.6
Favipiravir (CID:492405)	C_5_H_4_FN_3_O_2_	−4.61	−6.2
Galidesivir (CID:10445549)	C_11_H_15_N_5_O_3_	−5.51	−6.6
Remdesivir (CID:121304016)	C_27_H_35_N_6_O_8_P	−3.51	−8.1
Ribavirin (CID:37542)	C_8_H_12_N_4_O_5_	−4.05	−6.9
Sofosbuvir (CID:45375808)	C_22_H_29_FN_3_O_9_P	−4.13	−8.2

### 3.7. Toxicity measurement

Following the molecular docking analysis, the top 12 compounds selected from the previous screenings were evaluated for toxicity using the ProTox II server. The evaluation encompassed a range of toxicological factors, including carcinogenicity, hepatotoxicity, acute toxicity, cytotoxicity, and mutagenicity, and the calculation of the LD50 (median lethal dosage) in milligrams per kilogram. These findings are displayed in Table 8. Based on the findings by ProTox II, compounds classified in class 4 or higher were further evaluated for specific types of toxicity. CNP0331352.1 and CNP0202263.1 have a high LD50 of 5000 mg/kg and 3000 mg/kg respectively (Class V), indicating low acute toxicity. However, it exhibits very low mutagenic properties. A comparable FDA-approved drug with similar characteristics is Benznidazole, a drug used for treating Chagas disease, which also has a high LD50 but carries mutagenic risks due to the formation of reactive radical species that can damage DNA. Despite their mutagenic effects, benznidazole drugs are used for treatment because their therapeutic benefits outweigh the potential risks, especially in life-threatening diseases. Similarly, CNP0331352.1 and CNP0202263.1 can be considered antiviral drugs as it has very low mutagenic effect with high binding affinity towards NS5 protein [103]. ZINC000103114410, with an LD50 of 1000 mg/kg (Class IV), is hepatotoxic, making it comparable to the antiviral drug Dasabuvir, which has a similar LD50 and is known to cause liver-related side effects. This suggests that both compounds undergo hepatic metabolism, potentially generating toxic metabolites that affect liver function. On the other hand, ZINC000514287430, which has an LD50 of 2300 mg/kg (Class V) and no significant toxicity, is comparable to Galidesivir, an antiviral drug with a relatively safe profile and no major toxic effects reported. This suggests that the compound might be a promising candidate for drug development with fewer long-term risks. Similarly, 136046538, with an LD50 of 1000 mg/kg (Class IV) and no reported toxic effects. The compounds ZINC000504372972, CNP0272687.1, LIGR20, LIGR12, LIGR19, and CNP0379615.3 exhibit diverse toxicity profiles, with some displaying mutagenicity and cytotoxicity, while others show no significant toxic effects. ZINC000504372972, with an LD50 of 7 mg/kg (Class II), is highly acutely toxic and exhibits both mutagenicity and cytotoxicity. CNP0272687.1, with an LD50 of 5000 mg/kg (Class V), has no reported toxic effects, suggesting a relatively safe profile. The LIGR20 and LIGR19 have an LD50 of 800 mg/kg (Class IV) and are both associated with hepatotoxicity. LIGR12, with the same LD50 but no observed toxicity. Lastly, CNP0379615.3, with an LD50 of 7 mg/kg (Class II) and no major toxicity markers, presents a case where high acute toxicity may limit its therapeutic window. Overall, while some of these compounds show promise for drug development, their safety must be carefully evaluated to balance efficacy with potential toxic risks. Table 8 provides a thorough compendium of the compounds selected for toxicity prediction using virtual screening data. Subsequently, based on the ProTox II results, compounds Dasabuvir (L1), CNP0331352.1(L2), ZINC000103114410(L3), CNP0202263.1(L4), ZINC000514287430(L5), 136046538(L6), and CNP0272687.1(L7) were chosen for further analysis focusing on ADME (Absorption, Distribution, Metabolism, and Excretion) considerations.

**Table 8:** ProTox II: Selected drugs toxicity determination.

Name of Drug	Oral Toxicity		Organ toxicity-Hepatotoxicity	Carcinogenicity	Mutagenicity	Cytotoxicity
	LD50 (mg/kg)	Class				
CNP0331352.1	5000	V	IA	IA	A	IA
ZINC000103114410	1000	IV	A	IA	IA	IA
CNP0202263.1	3000	V	IA	IA	A	IA
ZINC000514287430	2300	V	IA	IA	IA	IA
136046538	1000	IV	IA	IA	IA	IA
ZINC000504372972	7	II	IA	IA	A	A
CNP0272687.1	5000	V	IA	IA	IA	IA
LIGR20	800	IV	A	A	IA	IA
LIGR12	800	IV	IA	IA	IA	IA
LIGR19	800	IV	A	A	IA	IA
CNP0379615.3	7	II	IA	IA	IA	IA
Dasabuvir	1000	IV	A	IA	IA	IA
Favipiravir	1717	IV	IA	A	IA	IA
Galidesivir	500	IV	IA	IA	IA	IA
Remdesivir	1000	IV	IA	IA	IA	IA
Ribavirin	2700	V	IA	A	IA	IA
Sofosbuvir	12000	VI	A	IA	IA	IA

IA: Inactive; A: Active.

### 3.8. ADME analysis

Following the toxicity evaluation, the seven selected compounds were further assessed for their physicochemical and pharmacokinetic properties to evaluate their suitability for oral activity. This evaluation was performed using the Swiss ADME tool [104], which provided insights into key drug-like properties and are summarized in supporting information S8 Table. This evaluation involved assessing compliance with Lipinski’s Rule of Five (RO5) and Veber’s Rule to ascertain their suitability for oral activity [100,105]. S8 Table provides a summary of how all the chosen compounds violated both Lipinski and Veber’s rule requirements. The SwissADME analysis of the selected molecules reveals diverse drug-like properties, absorption profiles, and metabolic interactions. Molecular weights range from 415.4 Da (ZINC000103114410) to 493.57 Da (Dasabuvir), with most compounds adhering to Lipinski’s rule, except CNP0331352.1 (L2), which has a high TPSA (189.26 Å²), suggesting poor permeability. Most compounds have a moderate number of rotatable bonds (≤6), indicating reasonable molecular flexibility. Lipophilicity, which enhances the solubility of drug-like substances in fats and oils, increases their dispersion through cell membranes, indicating the possibility for oral delivery. In terms of lipophilicity, Dasabuvir (L1) exhibits the highest Log P (3.80), indicating higher lipid solubility, whereas CNP0331352.1 (L2) has the lowest Log P (0.38), making it highly hydrophilic. The solubility of the chemicals varied, which is an important factor that affects their bioavailability and bioactivity [106]. Water solubility (ESOL Log S) varies, with CNP0331352.1 (L2), ZINC000514287430 (L5), and CNP0272687.1 (L7) classified as “soluble,” while others are “moderately soluble.” Poor solubility but near the insoluble range (e.g., Dasabuvir with Log S = −5.65) can limit bioavailability and require formulation strategies. The solubility of the chemicals varied, which is an important factor that affects their bioavailability and bioactivity [107].

The physicochemical properties of the selected compounds provide insights into their drug-likeness, permeability, and potential bioavailability is assessment through molar refractivity (MR) and topological polar surface area (TPSA) [108,109]. Among them, Dasabuvir (L1) has the highest molar refractivity (MR: 139.63) and moderate topological polar surface area (TPSA: 118.64 Å²), indicating good permeability and molecular interaction potential. CNP0331352.1 (L2) exhibits a low MR (110.74) but a very high TPSA (189.26 Å²), which suggests poor passive absorption, likely requiring alternative delivery methods. ZINC000103114410 (L3) has a moderate MR (120.45) and a low TPSA (100.46 Å²), making it the most promising candidate for oral bioavailability. CNP0202263.1 (L4) presents a high MR (124.46) and a slightly elevated TPSA (145.91 Å²), which may pose some absorption challenges but suggests strong molecular interactions. ZINC000514287430 (L5) and 136046538 (L6) have higher MR values (139.44 and 128.64, respectively), with TPSA values of 113.02 Å² and 176.29 Å², respectively, indicating varied permeability potential. Lastly, CNP0272687.1 (L7) has a moderate MR (118.97) and high TPSA (159.8 Å²), suggesting moderate solubility but limited passive absorption. Overall, L3 appears to have the most favorable balance between permeability and molecular interactions, while L2 and L7 may face challenges in oral bioavailability due to their high TPSA values. Synthetic accessibility (SA) scores indicate the ease of synthesizing a compound, with lower values suggesting higher feasibility [109]. Among the selected compounds, Dasabuvir (L1) has the lowest SA score (3.46), making it the most easily synthesizable. ZINC00103114410 (L3) also has a relatively low SA score (3.7), suggesting good synthetic feasibility. In contrast, ZINC000514287430 (L5) has the highest SA score (6.34), indicating that it is the most challenging to synthesize, possibly due to complex structural features or intricate functional groups. CNP0331352.1 (L2) and CNP0202263.1 (L4) have moderate SA scores of 5.29 and 5.36, respectively, implying a significant level of synthetic effort. Meanwhile, 136046538 (L6) and CNP0272687.1 (L7) have scores of 4.18 and 5.37, respectively, indicating moderate synthetic complexity. Overall, L1 (Dasabuvir) and L3 exhibit the highest synthetic accessibility, whereas L5 presents the most considerable synthetic challenge.

Pharmacokinetic studies evaluated criteria such as the capacity of a substance to pass through the blood-brain barrier (BBB), its absorption in the gastrointestinal tract (GI), and its interactions with permeability glycoprotein (P-gp) [110]. Pharmacokinetically, ZINC000103114410 (L3) and ZINC000514287430 (L5) exhibit high gastrointestinal (GI) absorption, making them better oral candidates, while others have low absorption, potentially requiring alternative formulations [111]. None of the compounds cross the blood-brain barrier (BBB) [112,113]. In terms of skin permeability, CNP0331352.1 (L2) has the lowest log Kp (−9.03 cm/s), indicating minimal transdermal absorption. Drug-likeness assessments show that most molecules have ≤1 Lipinski violation, except CNP0331352.1 (L2), which violates both Lipinski and Veber rules due to its high polarity. The search for possible non-specific interaction with biological targets led to the identification of PAINS (Pan Assay INterference chemicals) [114,115]. PAINS alerts are found in CNP0331352.1 (L2) and ZINC000103114410 (L3), suggesting potential promiscuous binding. From a metabolism perspective, ZINC000103114410 (L3) and Dasabuvir (L1) inhibit multiple CYP enzymes (CYP2C9, CYP2C19, CYP1A2), raising concerns for drug-drug interactions, while ZINC000514287430 (L5) is a P-gp substrate, meaning it may undergo active efflux, reducing intracellular concentration.

In conclusion, ZINC000103114410 (L3) and ZINC000514287430 (L5) emerge as promising oral candidates due to high GI absorption, whereas CNP0331352.1 (L2) and Dasabuvir (L1) face absorption challenges. CYP inhibition risks for ZINC000103114410 (L3) and Dasabuvir (L1) necessitate cautious co-administration, while PAINS alerts in CNP0331352.1 (L2) and ZINC000103114410 (L3) suggest potential false positives in bioassays. The findings highlight the need for further optimization to balance bioavailability, permeability, and metabolic stability in drug development. ZINC000103114410 (L3) and ZINC000514287430 (L5) are the best candidates for further investigation, particularly for oral drug development. CNP0331352.1 (L2) and CNP0202263.1 (L4) may not be the best candidate for oral drug development due to its low GI absorption, high polarity (TPSA = 189.26 Å²), and PAINS alert, which indicates potential promiscuous binding. However, its high water solubility and low lipophilicity (Log P = 0.38) suggest it could be suitable for intravenous administration, where GI absorption is not a concern.

### 3.9. DFT calculations

Following the pharmacokinetic and toxicity evaluations, the seven selected compounds underwent geometry optimization using Density Functional Theory (DFT) in Orca 4.2. The lowest energy geometry corresponds to the most stable conformation of a molecule, as molecules naturally tend to adopt the configuration with the least energy [116]. By applying DFT calculations, we determined the molecular shape that was most optimal for the lowest energy state. This step is essential as it provides a precise description of the electronic structure and ensures accurate evaluation of molecular stability and reactivity. DFT is critical for predicting molecular properties at a quantum mechanical level. It helps us assess factors such as stability, reactivity, and the energy gap between the Highest Occupied Molecular Orbital (HOMO) and the Lowest Unoccupied Molecular Orbital (LUMO). The calculated FMO (Frontier Molecular Orbitals) energy gap serves as an indicator of kinetic stability and chemical reactivity [117]. A larger FMO energy gap suggests increased stability and reduced reactivity, which is an essential factor for drug design, ensuring that the compounds will be less reactive and more stable under physiological conditions. The optimized energy values for ligands L1 to L7 are shown in Table 9, with their corresponding HOMO-LUMO energy gap values with orbitals of the selected ligands provided in S3 Fig. These values reflect the kinetic stability of the compounds. For instance, ligand L5 exhibited a larger energy gap, suggesting greater stability, whereas ligand L6 showed a smaller energy gap, indicating weaker stability. The 2D and 3D optimized structures of these ligands, along with the superimposed structure before and after optimization, are provided in the Supplementary Information (S2 Fig). The large HOMO-LUMO energy gap in the ligands also suggests that their binding affinity during molecular docking did not exhibit significant differences between the optimized and initial structures, supporting their stability and low reactivity during drug-protein interactions.

**Table 9:** FMO energy band values of ligand.

Ligand	Optimized Energy (eV)	HOMO (eV)	LUMO (eV)	Energy Gap (eV)
Dasabuvir (L1)	−52881.76	−5.857	−1.581	4.275
CNP0331352.1 (L2)	−46575.22	−6.039	−2.091	3.947
ZINC00103114410 (L3)	−38782.75	−5.799	−1.854	3.944
CNP0202263.1 (L4)	−45748.86	−5.682	−1.940	3.742
ZINC000514287430 (L5)	−43621.34	−5.806	−0.898	4.907
136046538 (L6)	−58331.95	−5.743	−2.409	3.334
CNP0272687.1 (L7)	−45.625.05	−6.163	−2.266	3.896

### 3.10. Redocking and interaction analysis

Following DFT-based geometry optimization, which confirmed the structural stability and electronic properties of the selected ligands, a redocking analysis was performed to evaluate how structural refinement influences binding interactions. The redocking procedure was implemented via AutoDock 4.0 implemented in PyRx 0.8, which involved the NS5 protein and ligands that were geometrically optimized. This step was essential to ensure that the optimized conformations retained or improved their binding capability with the target protein. Interestingly, after optimization, all the ligands demonstrated a decrease in binding affinity values, indicating that the lower-energy conformations may result in tighter and more favorable interactions within the protein’s active site. After optimization, all the ligands showed a decrease in binding affinity value. Table 10 shows the binding affinity of ligand before and after geometric optimization.

**Table 10:** Ligand binding affinity values before and after geometry optimization.

Compound ID	Name of the compound	Binding affinity(kcal/mol)	
		Before optimization	After optimization
Dasabuvir (L1)	Dasabuvir	−8.86	−6.06
CNP0331352.1 (L2)	6,7-dihydroxy-13-methoxy-14-[(2S,3R,4R,5R,6S)-3,4,5-trihydroxy-6-methyl-tetrahydropyran-2-yl]oxy-2,9-dioxatetracyclo[6.6.2.0^{4,16}.0^{11,15}]hexadeca-1(14),4,6,8(16),11(15),12-hexaene-3,10-dione	−8.28	−5.21
ZINC00103114410 (L3)	5-(1,3-benzodioxol-5-ylmethyliminomethyl)-6-hydroxy-1- naphthalen-1-ylpyrimidine-2,4-dione	−7.94	−7.14
CNP0202263.1 (L4)	6,12-dihydroxy-8-methoxy-3-methyl-1-[(2S,3R,4R,5R,6S)-3,4,5-trihydroxy-6-methyloxan-2-yl]oxy-12H-benzo[a]anthracen-7-one	−7.61	−4.44
ZINC000514287430 (L5)	(1R,5S,8S,20S,23S)-23-hydroxy-6-(pyridin-3-ylmethyl)- 17,24-dioxa-3,6,9-triazatetracyclo[18.3.1.15,8.011, 16]pentacosa-11,13,15-triene-4,10-dione	−7.50	−6.08
136046538 (L6)	4-(7-Amino-1,1-dioxo-1,2-dihydro-1lambda6-benzo [1,2,4]thiadiazin-3-yl)-5-hydroxy-2-(3-methyl-butyl)-6-thiophen-2-yl-2H-pyridazin-3-one	−7.42	−5.99
CNP0272687.1 (L7)	8-(7-hydroxy-2-oxo-chromen-6-yl)-7-[(2S,3R,4R,5R,6S)-3,4,5-trihydroxy-6-methyl-tetrahydropyran-2-yl]oxy-chromen-2-one	−7.35	−4.48

**Fig 4:**
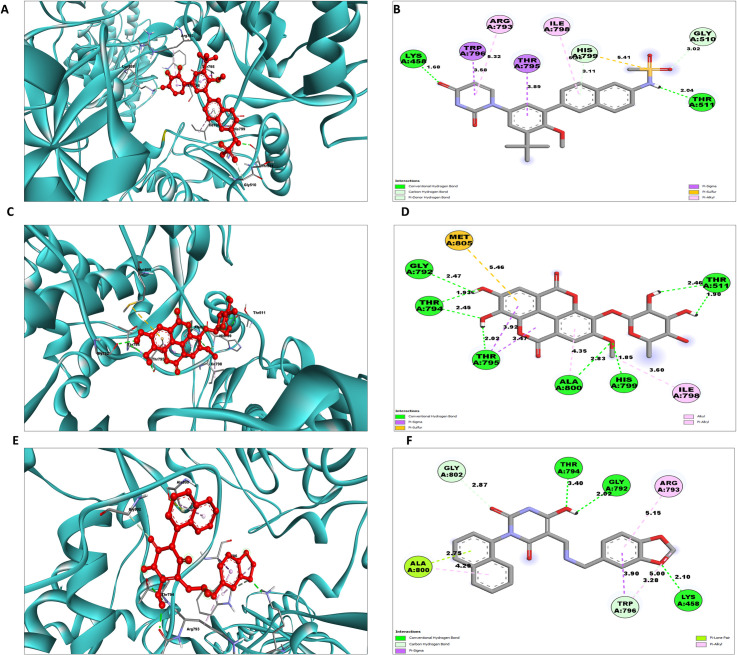
NS5 protein (blue ribbon model)-ligand (ball & stick (red)) 3D interaction analysis (A, C, E) and 2D interaction plot (stick model) (B, D, F) of NS5-L1, L2, and L3 complex respectively.

**Fig 5:**
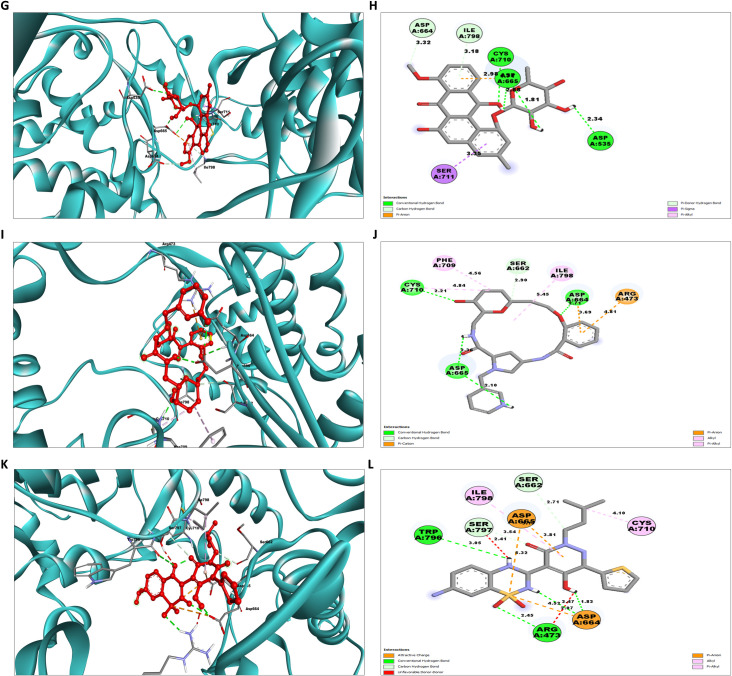
NS5 protein (blue ribbon model)-ligands (ball & stick (red)) 3D interaction analysis (G, I, K) and 2D interaction plot (stick model) (H, J, L) for NS5-L4, L5, and L6 complex respectively.

To obtain a more in-depth understanding, a thorough examination of protein-ligand interactions was accompanied to detect the precise amino acid residues that are associated. The development of pharmaceuticals and the management of disease both heavily depend on these interactions. Consequently, the interaction between the ligands and the NS5 protein was comprehensively analyzed using BIOVIA Discovery Studio Visualizer. The findings of this investigation of interactions, which encompass HB (conventional, pi-donor H-B, and carbon HB), diverse HP contacts (pi-alkyl, alkyl, pi-sigma, and pi-pi T-shaped), and electrostatic interactions (pi-anion), are displayed in Table 11 and Figs 4 and 5.

**Table 11:** NS5 protein-ligand interaction analysis.

Complex	Amino Acid Residue	Bond Distance (Å)	Bond Category	Type of Bond	Chemistry
L1 Dasabuvir	Lys458	1.679	HB	CHB	H-Donor
	Thr511	2.037	HB	CHB	H-Acceptor
	Gly510	3.016	HB	Carbon HB	H-Donor
	His799	3.115	HB	Pi-Donor	H-Donor
	Thr795	3.893	HP	Pi-Sigma	Non polar C-H
	Trp796	3.580	HP	Pi-Sigma	Non polar C-H
	His799	5.408	HP	Pi-Sulfur	Pi-Orbitals
	Ile798	5.359	HP	Pi-Alkyl	Alkyl
	Arg793	5.320	HP	Pi-Alkyl	Alkyl
L2	Thr794	2.450	HB	CHB	H-Donor
	Thr794	1.925	HB	CHB	H-Donor
	His799	1.850	HB	CHB	H-Donor
	Ala800	2.830	HB	CHB	H-Donor
	Thr511	2.458	HB	CHB	H-Acceptor
	Thr511	1.902	HB	CHB	H-Acceptor
	Thr795	2.016	HB	CHB	H-Acceptor
	Gly792	2.473	HB	CHB	H-Acceptor
	Thr795	3.474	HP	Pi-Sigma	Non polar C-H
	Thr795	3.924	HP	Pi-Sigma	Non polar C-H
	Met805	5.464	HP	Pi-Sulfur	Sulfur
	Ile798	3.605	HP	Alkyl	Alkyl
	Ala800	4.346	HP	Pi-Alkyl	Alkyl
L3	Lys458	2.103	HB	CHB	H-Donor
	Thr794	1.987	HB	CHB	H-Donor
	Thr794	1.931	HB	CHB	H-Donor
	Gly792	2.021	HB	CHB	H-Acceptor
	Trp796	3.282	HB	Carbon HB	H-Donor
	Gly802	2.873	HB	Carbon HB	H-Donor
	Trp796	3.899	HP	Pi-Sigma	Non polar C-H
	Ala800	2.750	HP	Pi-Lone Pair	Pi-Orbitals
	Trp796	4.998	HP	Pi-Alkyl	Pi-Orbitals
	Arg793	5.155	HP	Pi-Alkyl	Alkyl
	Ala800	4.951	HP	Pi-Alkyl	Alkyl
	Ala800	4.290	HP	Pi-Alkyl	Alkyl
L4	Cys710	1.709	HB	CHB	H-Donor
	Asp665	1.810	HB	CHB	H-Acceptor
	Asp665	2.881	HB	CHB	H-Acceptor
	Asp535	2.342	HB	CHB	H-Acceptor
	Asp664	3.321	HB	Carbon HB	H-Acceptor
	Asp665	2.977	Electrostatic	Pi-Anion	Negative
	Ile798	3.179	HB	Pi-Donor	H-Donor
	Ser711	3.347	HP	Pi-Sigma	Non polar C-H
	Ile798	5.444	HP	Pi-Alkyl	Alkyl
L5	Asp664	2.713	HB	CHB	H-Donor
	Cys710	2.207	HB	CHB	H-Donor
	Asp665	2.365	HB	CHB	H-Acceptor
	Asp665	2.098	HB	CHB	H-Acceptor
	Ser662	2.901	HB	Carbon HB	H-Acceptor
	Arg473	4.815	Electrostatic	Pi-Cation	Positive
	Asp664	3.691	Electrostatic	Pi-Anion	Negative
	Cys710	4.845	HP	Alkyl	Alkyl
	Ile798	5.446	HP	Alkyl	Alkyl
	Phe709	4.555	HP	Pi-Alkyl	Pi-Orbitals
L6	Asp664	4.520	Electrostatic	Electrostatic	Negative
	Asp665	5.323	Electrostatic	Electrostatic	Negative
	Arg473	2.454	HB	CHB	H-Donor
	Asp664	2.473	HB	CHB	H-Acceptor
	Trp796	3.051	HB	CHB	H-Acceptor
	Asp664	1.817	HB	CHB	H-Acceptor
	Ser797	3.537	HB	Carbon HB	H-Donor
	Ser662	2.707	HB	Carbon HB	H-Acceptor
	Asp665	3.807	Electrostatic	Pi-Anion	Negative
	Cys710	4.104	HP	Alkyl	Alkyl
	Ile798	5.128	HP	Pi-Alkyl	Alkyl

HB: Hydrogen Bond; HP: Hydrophobic; CHB: Conventional H-B.

The examination of the interaction between the protein and the L2 complex (Fig 4C and 4D) identified a total of 13 bonding interactions. These included 8 HB, consisting of 8 conventional bonds (CHB), as well as 5 hydrophobic (HP) contact consists of 2 Non-polar C-H, 1 sulphur and 2 alkyl interaction. The observed interactions were associated with a binding energy of −8.28 kcal/mol. The amino acid residues Thr794 (HH − O), His799 (HN − O), Ala800 (HN − O), Thr511, Thr795, Gly792 (O-HN) formed typical CHB with the ligand. Additionally, Thr797 participated in Non-polar C-H bond, Met805 participated in sulphur interaction and Ile798 and Ala800 participated in alkyl interaction.

The study of the interaction between the NS5-L3 complex (Fig 4E and 4F) revealed the presence of 12 bonding interactions. These interactions include 6 hydrogen bonds (4 CHB and 2 carbon HB bond) as well as 6 HP interactions (4 alkyl, 1 nonpolar CH, and 1 pi-orbital). The interactions were correlated with a binding energy of −7.94 kcal/mol. The residue Lys458, Thr794, and Gly792 established CHB with the ligand through H − O interactions, whereas Trp796 and Gly802 participated in carbon-HB. Trp796 formed two alkyl interaction, whereas Ala800 participated in pi-orbital interactions with the ligand. The residues Arg793 and Ala800 participated in pi-alkyl interaction with ligand.

Regarding the NS5 protein interaction with the L4 complex (Fig 5G and 5H), a total of 9 bonding interactions were identified: 6 HB (4 CHB, 1 Carbon HB, and 1 pi-donor), 1 electrostatic interactions, and 2 HP contacts (1 nonpolar-CH and 1 pi-alkyl). The energy required to link these entities together was −7.61 kilocalories per mole. The residues Cys710, Asp605, and Asp535 engaged in CHB with the ligand through H − O interactions, whereas Asp664 established a carbon-hydrogen bond with the ligand by an HC—O interaction, whereas Ile798 involved in HB (pi-donor0 with ligand. The ligands were involved in electrostatic pi-anion interactions with Ile798, Asp665, and Ser711 were involved in pi-orbital and pi-alkyl interactions with the ligand, respectively.

The examination of the protein’s interaction with the L5 complex (Fig 5I and 5J) uncovered a total of 10 bonding interactions: 5 HB (4 conventional and 1 carbon-HB), 2 electrostatic interaction, and 3 pi-alkyl HP interactions. The binding energy of this interaction was −7.50 kcal/mol. The ligand produced CHB with the amino acids Asp664, Cys710, and Asp665. Additionally, a carbon-HB was formed with the amino acid residue Ser662. The residues Arg473 and Asp664 created an electrostatic contact with the ligand via pi-cation and pi-anion bonding respectively, whereas Cys710, Ile798, and Phe709 established associations with the ligand involving alkyl and pi-alkyl bonding respectively.

The analysis of the protein’s interaction with the L6 complex (Fig 5K and 5L) showed a total of 12 bonding interactions: 7 HB (5 conventional and 2 Carbon H-B bond), 3 electrostatic interactions (2 negative charge and 1 pi-anion), and 2 HP interactions (1 alkyl and 1 pi-alkyl). The interaction demonstrated a binding affinity of −7.42 kcal/mol. The residues Arg473, Asp664, and Trp796 formed CHB with the ligand, whereas Ser797 and Ser662 contributed to carbon- HB. Asp664 and Asp665 residue established three electrostatic interactions, Cys710 and Ile798 formed alkyl and pi-alkyl interaction with ligand. After analyzing this interaction, we selected ligands L2, L3, and L4 together with dasabuvir for further simulation tests.

To compare the interaction analysis of the designed ligand with the FDA approved antiviral, the examination of the NS5 protein and dasabuvir interaction demonstrated a total of 9 bonding interactions (Fig 4A and 4B). The molecule forms a total of 4 HB, consisting of 2 CHB, 1 pi-donor, and 1 carbon HB. Furthermore, there are 5 HP contacts, including 2 nonpolar CH, 1 pi-orbital and 2 pi-alkyl interaction. The interactions exhibited a binding energy of −8.86 kcal/mol. The amino acids Lys458 and Thr511 established carbon-HB with the ligand, whereas Gly510 and His799 established 1 Carbon-HB and 1 pi-donor HB with ligand. The ligand formed pi-sigma and nonpolar CH interactions with Thr795 and Trp796 residues of NS5 protein, whereas His799 established pi-sulfur interaction with ligand. The residues Ile798 and Arg793 established alkyl interactions with the ligand.

Among the designed ligands, L2 exhibited the strongest binding affinity (−8.28 kcal/mol) and the highest number of interactions (13 total), including eight conventional hydrogen bonds and diverse hydrophobic contacts, making it the most promising candidate. L3 and L4 also showed favorable interactions and binding energies (−7.94 and −7.61 kcal/mol), while L5 and L6 displayed slightly weaker affinities and fewer critical contacts. Compared to the reference drug dasabuvir (−8.86 kcal/mol), L2 closely matched in interaction diversity and strength, suggesting its potential as a viable NS5 inhibitor.

### 3.11. Molecular dynamics (MD) simulation

Molecular dynamics (MD) simulation was performed to investigate the structural stability and dynamic behavior of the NS5 protein and its complexes with selected ligands. The simulation aimed to provide insights into the conformational changes, interaction stability, and flexibility of the protein-ligand complexes under physiological conditions. Using Desmond, the systems were prepared, solvated, and simulated under NPT ensemble for 100 ns. The resulting trajectories were analyzed to assess backbone deviations, residue fluctuations, interaction persistence, and conformational stability. These evaluations supported the reliability of the docking results and helped identify ligands with consistent and stable binding behavior over time.

#### 3.11.1. MD simulation of NS5 protein.

**Fig 6:**
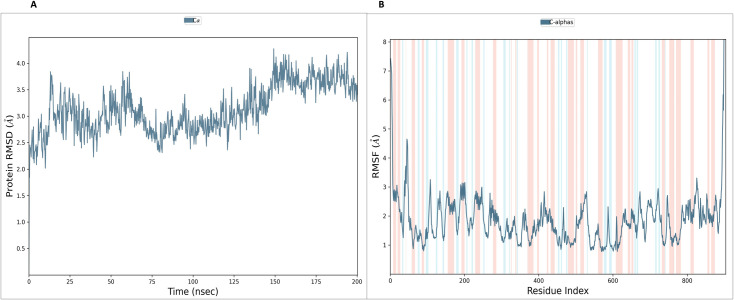
(A) RMSD and; (B) RMSF analysis of NS5 protein at 200 ns.

To assess the stability and convergence of the NS5 protein, MD simulations were performed over 200 ns in triplicates. The Root Mean Square Deviation (RMSD) of the NS5 protein started at 1.9 Å at 0 ns, gradually increased to 3.9 Å at 20 ns, and fluctuated between 2.2 and 3.9 Å from 20 to 150 ns and later increased to 4.2 Å at 152 ns before stabilizing around 3.5 Å after 190 ns (Fig 6A). This RMSD range is acceptable, indicating no major conformational changes. This RMSD range is acceptable in the range 1.84 to 4.316 Å in triplicates, indicating no major conformational changes. The average RMSD of NS5 protein was found to be 3.13 ± 0.45 Å Stable RMSD values suggest good simulation convergence and stable protein conformations. The Root Mean Square Fluctuations (RMSF) plot showed notable fluctuations in regions other than the α-helix and β-sheets, with more fluctuation at the N and C-terminals compared to internal amino acids (Fig 6B). The RMSF Figs indicate that the protein structure maintains its rigidity throughout the simulation. The average RMSD of NS5 protein was found to be 1.872 ± 0.92 Å. The RMSF was fluctuated between 0.287 to 9.981 Å in triplicates. The RMSF graphic includes annotations for secondary structural features, including alpha-helical areas (highlighted in red) and beta-stranded regions (highlighted in blue). The RMSD and RMSF trajectory of NS5 protein of replica 2 was represented in supplementary S4 Fig.

#### 3.11.2. RMSD of NS5 protein-ligand complex.

**Fig 7:**
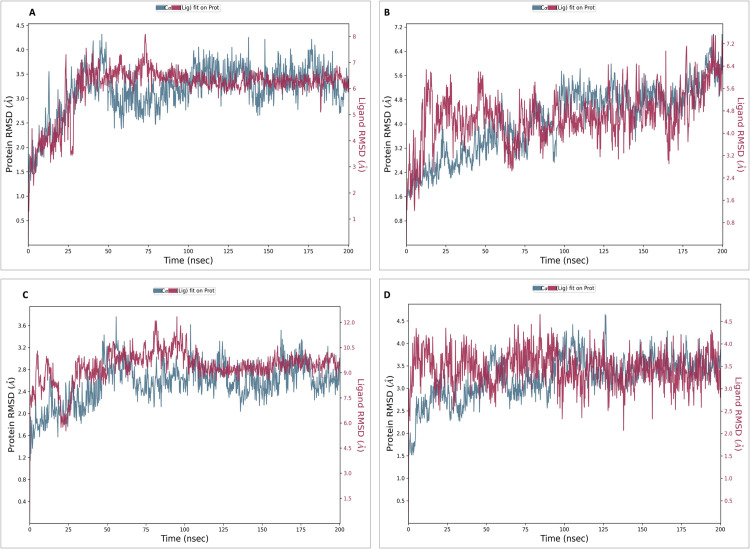
RMSD trajectory of NS5-ligand complex at 200 ns (A) NS5-L3 complex, (B) NS5-L5 complex, (C) NS5-L6 complex, & (D) NS5-dasabuvir complex.

The NS5 protein-ligand complex’s RMSD trajectory is shown graphically in Fig 7. The accompanying image shows the protein’s RMSD trajectory in blue, along with the associated RMSD values displayed in Å units on the left side of the Y-axis. Furthermore, the ligand’s RMSD trajectory is shown in red in the Fig, along with the matching values in Å units on the Y-axis (right). The RMSD of the NS5-L1 (Dasabuvir) complex (Fig 7A) showed oscillations, starting at 0 ns at approximately 1.5 Å and rising to 4.2 Å by 50 ns. After that, the protein’s RMSD trajectory fluctuated between 2.5 Å to 4.0 Å from 50 ns to 120. At the end of the 190 ns MD simulation, the protein RMSD stabilized at 3.6 Å and remained stable. The protein’s RMSD showed oscillations mostly between 2.5 and 4.0 Å, indicating that the protein stayed in a stable structure during the simulation. The protein’s RMSD showed oscillations mostly between 1.247 and 4.316 Å with an average RSMD of 3.173 ± 0.504 Å in triplicates, indicating that the protein stayed in a stable structure during the simulation. In contrast, the RMSD trajectory of the L1 ligand began at 1.0 Å but climbed significantly, reaching about 7.2 Å at 25 ns. Following this, the L1 ligand RMSD varied from 25 to 100 ns, ranging from 6.5 to 8.0 Å. Sharp fluctuations in the ligand’s RMSD were present up to 75 ns of the simulation time. After 100 ns, the ligand RMSD reached stability up to 200 ns of simulation time at 6.0 Å. The ligand RMSD showed oscillations mostly between 1.297 and 8.082 Å with an average RSMD of 6.046 ± 1.297 Å both in triplicates. Overall, the ligand’s RMSD indicates greater stability within the active binding site of the NS5 protein.

The RMSD of the NS5-L2 complex (Fig 7B), which increased gradually to 4.0 Å at 25 ns from 1.6 Å at 0 ns. At 100 ns, the protein’s RMSD trajectory increased to 5.6 Å. additionally, the protein RMSD increased and varied up to 6.6 Å at 190 ns. The protein RMSD showed larger fluctuations around 200 ns of simulation time. The protein’s RMSD showed oscillations mostly between 1.49 and 6.962 Å with an average RSMD of 4.122 ± 1.11 Å in triplicates, indicating that the protein stayed in a stable structure during the simulation. In contrast, the RMSD trajectory of the L2 ligand began at about 1.5 Å and increased quickly to 6.4 Å at 20 ns, with higher variations. Following this, the L2 ligand RMSD fluctuated between 3.2 and 7.2 Å between 50 and 200 ns. The L2-ligand RMSD showed oscillations mostly between 1.231 and 7.536 Å with an average RSMD of 4.62 ± 0.93 Å in triplicates. Similar to protein RMSD, the ligand’s RMSD also fluctuated at a higher level by maintaining a stable position within the active site of the protein.

The NS5-L3 complex’s RMSD (Fig 7C) increased progressively to 2.8 Å at 20 ns from a starting point of about 1.2 Å at 0 ns. The protein’s RMSD trajectory then varied from 20 to 160 ns, ranging from 2.0 to 3.2Å. The protein RMSD stabilized at about 3.0 Å for the next 200 ns after 160 ns. When compared to other NS5 protein-ligand complexes, the protein’s RMSD fluctuated mostly between 1.2 and 3.6 Å, indicating that it kept a stable conformation throughout the simulation. The protein’s RMSD showed oscillations mostly between 1.147 and 3.76 Å with an average RSMD of 2.55 ± 0.36 Å in triplicates, indicating that the protein stayed in a stable structure during the simulation. In contrast, the RMSD trajectory of the L3 ligand began at about 7.5 Å but increased quickly to 10.5 Å at 10 ns with higher variations. Following this, the L3 ligand RMSD dropped to 6.0 Å at 25 ns and further increased to 12.0 Å at 100 ns and fluctuated between 7.5 to 12.0 Å from 30 to 100 ns. Further, the RMSD of the L3 ligand maintained stable RMSD at 9.0 Å after 100 ns throughout the simulation time of 200 ns. The L3-ligand RMSD showed oscillations mostly between 5.52 and 12.33 Å with an average RSMD of 9.38 ± 0.924 Å in triplicates. When compared to other protein-ligand complexes, the ligand’s RMSD generally indicates increased stability inside the NS5 protein’s active binding region.

Similarly, for the NS5 protein-L4 complex (Fig 7D), the protein RMSD started at 1.5 Å at 0 ns and later increased to the first step at 3.5 Å at 25 ns and a second step at 4.0 Å at 60 ns, and the third step at 4.5 Å at 125 ns. The protein RMSD fluctuated between 3.5 to 4.5 Å from 25 ns to 125 ns. Further, the protein RMSD stabilized around 4.0 Å up to 200 ns. The protein’s RMSD showed oscillations mostly between 1.519 and 4.642 Å with an average RSMD of 3.24 ± 0.47 Å in triplicates, indicating that the protein stayed in a stable structure during the simulation. With respect to the L4 ligand, the RMSD started at 2.0 Å at 0 ns and raised to 4.5 Å at 10 ns. Further, the RMSD of L4 ligand fluctuated between 2.5 to 4.5 Å up to 150 ns of simulation time. Further, ligand L4 stabilized around 4.0 Å up to 200 ns. The ligand RMSD showed oscillations mostly between 2.07 and 4.653 Å with an average RSMD of 3.450 ± 0.380 Å both in triplicates. This demonstrated that L4 ligand remained stable at NS5 protein’s active site. The RMSD trajectory of NS5-ligand complex of replica 2 was represented in supplementary S5 Fig.

#### 3.11.3. RMSF of NS5 protein-Ligand complex.

**Fig 8:**
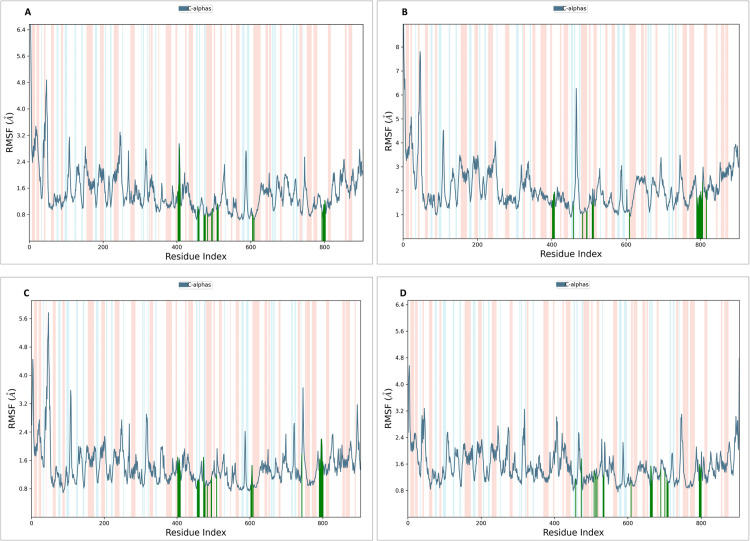
RMSF trajectory of NS5-ligand complex at 200 ns (A) NS5-L1 complex, (B) NS5-L2 complex, (C) NS5-L3 complex, & (D) NS5-L4 complex.

In Fig 8, the RMSF map of the NS5 protein-ligand complex shows green bars indicating amino acids that are interacting with the ligand. The average RMSF for most amino acids in the RMSF plots for NS5-L1 (Fig 8A), NS5-L2 (Fig 8B), NS5-L3 (Fig 8C), and NS4-dasabuvir (Fig 8D) ligand complexes remains below 3.2 Å, 4.5 Å, 3.2 Å, and 3.2 Å, respectively. The RMSF values for most of the amino acid residues involved in the interaction with the NS5 ligand complex were less than 2.0 Å, except for a small number of amino acids at the N and C terminals. The N & C-terminal area exhibited a particularly noticeable fluctuation, which was characterized by a loop shape and resulted in large changes in RMSF [118]. When compared directly to the protein’s unbound state (as shown in Fig 6B), the NS5 protein-ligand complex had reduced RMSF values. This is likely due to the ligand binding in the loop region of the protein. This indicates that the complex exhibits reduced RMSF in comparison to the protein in its unbound form. It is important to mention that secondary structural elements, such as alpha helices and beta strands, generally show less variation compared to loop regions (shown by a white background) in all NS5-ligand complexes. The RMSF trajectory of NS5-L1 ligand complex was fluctuated between 0.64 and 9.27 Å with an average of 1.495 ± 0.724 Å, for NS5-L2 complex between 0.895 and 9.669 Å with an average of 2.115 ± 0.925 Å, for NS5-L3 complex between 0.685 and 5.768 Å with an average of 1.432 ± 0.561 Å, and similarly for NS5-L4 complex between 0.754 and 4.788 Å with an average of 1.525 ± 0.502 Å. Similarly, RMSF trajectory for NS5-ligand complex for replica 2 at 200 ns was represented in supplementary S6 Fig.

S7 Fig presents the RMSF plot of the ligand for all NS5-ligand complexes. The mean RMSF values indicate that atomic displacement in the RMSF plots varied as follows: for the L1 ligand (S7A Fig), fluctuations ranged from 1.516 to 4.636 Å, with an average of 2.300 ± 0.914 Å; for the L2 ligand (S7B Fig), from 1.371 to 3.688 Å, average of 2.374 ± 0.698 Å; for the L3 ligand (S7C Fig), from 2.149 to 6.572 Å, with a mean of 3.104 ± 1.291 Å; and for the L4 ligand (S7D Fig), from 0.92 to 1.543 Å, with an average of 1.126 ± 0.15 Å. These fluctuations suggest that the ligands dynamically explore multiple binding positions in search of a more stable conformation within the NS5 protein’s binding site. Similarly, the RMSF plot of all ligands for the NS5-ligand complexes in replica 2 is presented in Supplementary S8 Fig. A summary of the RMSD of the ligand-protein complexes and the RMSF values for both protein and ligand is provided in Table 12.

**Table 12:** Average value of RMSD and RMSF values of NS5 and NS5-ligand complex.

Molecule	Protein RMSD (Å)	Ligand RMSD (Å)	Protein RMSF (Å)	Ligand RMSF (Å)
**NS5**	3.13 ± 0.45	--	1.872 ± 0.92	--
**NS5-L1 complex**	3.173 ± 0.504	6.046 ± 0.937	1.495 ± 0.724	2.300 ± 0.914
**NS5-L2 complex**	4.122 ± 1.11	4.62 ± 0.93	2.115 ± 0.925	2.374 ± 0.698
**NS5-L3 complex**	2.552 ± 0.36	9.38 ± 0.924	1.432 ± 0.561	3.104 ± 1.291
**NS5-L4 Complex**	3.242 ± 0.475	3.450 ± 0.310	1.525 ± 0.502	1.126 ± 0.15

#### 3.11.4. Interaction diagram of NS5 protein-Ligand complex.

**Fig 9:**
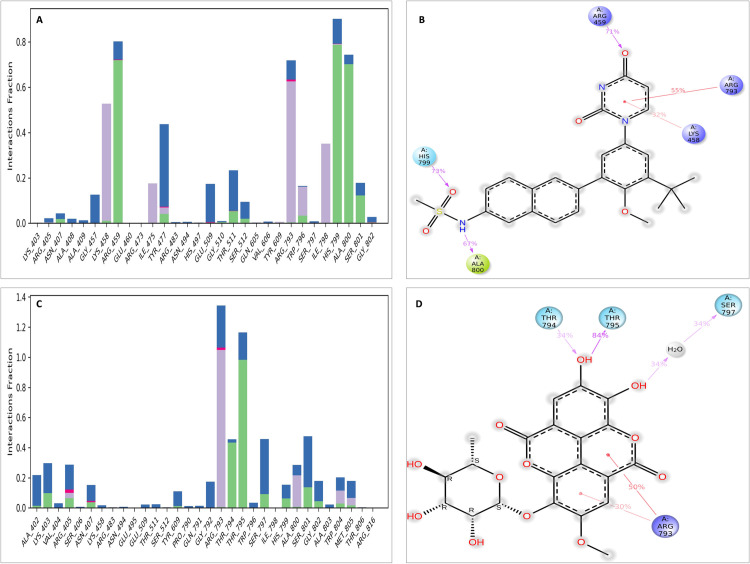
NS5-ligand interaction map (A) NS5-L1 complex, (C) NS5-L2 complex and NS5-ligand contact for more than 30% simulation time (B) NS5-L1 complex, (D) NS5-L2 complex.

**Fig 10:**
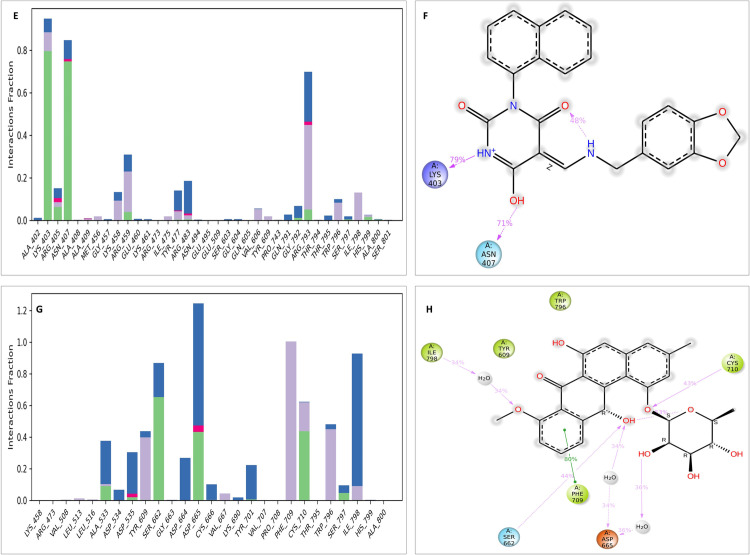
NS5-ligand interaction map (E) NS5-L3 complex, & (G) NS5-L4 complex and NS5-ligand contact for more than 30% simulation time (F) NS5-L3 complex, & (H) NS5-L4 complex.

The NS5 protein-ligand complex during the 200 ns MD simulation is depicted in Figs 9 and 10, where four distinct forms of protein-ligand contacts are identified: hydrophobic, ionic, hydrogen bonds, and water bridges. During the course of the analysis, these stacked bar charts are standardized; higher interaction fractions suggest longer-lasting interactions. According to the interaction map of the NS5-L1 complex (Fig 9A), L1 formed typical hydrogen bonds with amino acids and ligands during the MD simulation. The specific amino acids involved in these hydrogen bonds were His799, Ala800, Ser801, Asn407, Lys458, Arg459, Tyr477, Thr511, Ser512, and Trp796. The L3 ligand exhibits a higher number of interactions with the NS5 protein through water bridges compared to hydrogen bonds. Fig 9B provides a concise overview of the timeline illustrating the many interactions and contacts (such as hydrophobic, ionic, H-bonds, and water bridges) between the L1 ligand and NS5 protein, which occurred for more than 30% of the simulation duration. During the simulation, the amino acids Lys458, Arg793, Arg459, Ala800, and His799 maintained contact times of 32%, 55%, 71%, 67% and 73% respectively.

According to the interaction diagram (Fig 9C) of the NS5-L2 complex, L2 formed typical hydrogen bonds with amino acids and the ligand during the MD simulation. The amino acids involved in these bonds were Ala402, Cys403, Arg405, Asn407, Tyr609, Thr794, Thr795, Ser797, His799, Ser801, Gly807, Trp804, and Met805. The L2 ligand exhibits a greater number of interactions with the NS5 protein through water bridges and hydrophobic interactions compared to hydrogen bonds. Fig 9D provides a summary of the timeline illustrating the various interactions and contacts, such as hydrophobic, ionic, H-bonds, and water bridges, between the L2 ligand and NS5 protein. The interactions and contacts that occurred for more than 30% of the simulation duration are included in this summary. The amino acids Thr794, Thr795, Ser797, and Arg793 maintained contact times of 34%, 84%, 34%and 50%, respectively, during the simulation.

The NS5-L3 complex interaction diagram (Fig 10E) shows that during the MD simulation, L3 forms few hydrogen bonds with amino acids and the ligand. These amino acids include Lys403, Arg405, Asn407, Arg459, Gly792, Arg793, and His799. Fig 10F provides a summary of the timeline illustrating the interactions and contacts between the L3 ligand and NS5 protein. These interactions include hydrophobic, ionic, H-bonds, and water bridges. The overview focuses on the interactions that occurred for more than 30% of the simulation duration. The amino acids Lys403 and Asn407 maintained contact times of 79% and 71% respectively, during the simulation.

In the interaction diagram (Fig 10G) of the NS5-L4 complex, L4 formed traditional hydrogen bonds with several amino acids and the ligand during the MD simulation. These amino acids include Ala533, Asp535, Ser662, Asp665, Cys710, and Ser797. Fig 10H provides a concise overview of the timeline illustrating the many interactions and contacts (such as hydrophobic, ionic, H-bonds, and water bridges) between the L4 ligand and NS5 protein, specifically focusing on interactions that occurred for more than 30% of the simulation duration. The amino acids Ile798, Cys710, Asp665, Phe709, and Ser662 retained 34%, 43%, 34%, 80%, and 44% of their contact time, respectively, throughout the simulation. Similarly, the interaction diagram of all the NS5-ligand complexes of replica 2 was represented in supplementary S9 and S10 Figs.

#### 3.11.5. Interaction time line diagram of NS5 protein-Ligand complex.

S11 and S12 Figs depict the quantity of contact ligands (shown in the top panel) that interact with the NS5 protein. The bottom panel of the Fig displays a timeline diagram illustrating the interaction between the NS5 protein and the ligand complex. During the modeling of the NS5 protein-L1 complex (S11A Fig), the protein and ligand had a varying number of unique interactions, ranging from 1 to 12 interactions. Several residues, including Ala800, His799, Arg793, Arg459, and Lys458, exhibited multiple interactions with the ligand, as shown by a more intense orange color. The ligand underwent a shift in its initial binding location within the first 30 ns but thereafter remained stable from 30 to 200 ns. This is consistent with the information presented in Fig 7A, which demonstrates variations in the ligand’s RMSD and an unsteady binding position.

During the simulation, the NS5 protein-L2 complex (S11B Fig) exhibited a range of 1–14 unique interactions between the protein and ligand, involving amino acids. The ligand made multiple contacts with certain residues, namely Ser801, Ala800, Ser797, Thr794, and Arg793, as evidenced by a deeper orange tone. These findings indicate that the ligand underwent a change in its initial binding location during the MD simulation. This is consistent with Fig 7B, which demonstrates variations in the ligands’ RMSD and an unstable binding conformation.

During the simulation, the NS5 protein-L3 complex (S12C Fig) exhibited a range of 1–10 amino acids with unique interactions between the protein and ligand. Residues Arg793, Asn407, and Lys403 exhibited several interactions with the ligand, as evidenced by a darker orange tone. These residues maintained a consistent contact throughout the MD simulation, as depicted in Fig 6C, which also showed reduced variations in the ligand’s RMSD. As indicated in RMSD Fig 7C, very little interaction occurred during simulation, which can be correlated by only three amino acids interacting with the ligand.

In the case of the NS5 protein-L4 complex (S12D Fig), the simulation revealed that there were between 1 and 15 unique interactions between the protein and ligand involving amino acids. Residues Ile798, Trp796, Cys710, Phe709, Asp665, Ser662, Tyr609, Asp535, and Ala533 exhibited several interactions with the ligand, as indicated by an orange shade. The timeline Fig illustrates the strong interactions between the NS5 protein and the ligand during a 200 ns MD simulation, as evidenced by the Fig 7D protein-ligand RMSD. Similarly, the interaction timeline diagram of all the NS5-ligand complexes of replica 2 was represented in supplementary S13 and S14 Figs.

#### 3.11.6. Binding poses of NS5 protein-Ligand complex.

**Fig 11:**
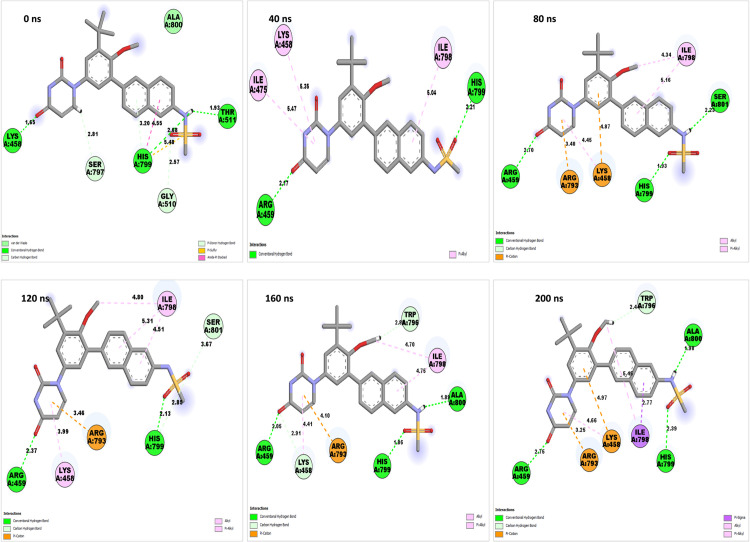
Binding poses of L1 ligand at the active site of NS5 protein at different time intervals during MD simulation.

Throughout the 200 nanosecond MD simulation, the ligands experienced conformational alterations within the active binding pocket of the NS5 protein. These changes occurred as the ligands sought to stabilize themselves by locating more appropriate binding positions depicted in Fig 11 and S15–S17 Figs, which is also apparent in the ligand RMSD trajectories. The ligands aimed to achieve stability by interacting with various amino acid residues over a period of time, as demonstrated by the protein-ligand interaction timeline depicted in Fig 9 for the NS5 protein-L1 complex, during this interaction timeline, ligand finds different amino acids which attach to the active binding pocket of the NS5 protein at various time periods, namely 0 ns, 40 ns, 80 ns, 120 ns, 160 ns, and 200 ns as indicated by Fig 11. Similarly, binding poses for other NS5 protein-ligand complex were depicted in supplementary information in S15 Fig for NS5 –L2 complex, S16 Fig for NS5 –L3 complex, and S17 Fig for NS5 –L4 complex at various time periods, namely 0 ns, 40 ns, 80 ns, 120 ns, 160 ns, and 200 ns.

#### 3.11.7. Binding free energy analysis of NS5 protein-Ligand complex.

In this study, we employed the Prime MM-GBSA method to estimate the binding free energies of ligand-protein complexes during molecular dynamics simulations. We evaluated energy components such as electrostatic interactions, covalent contributions, hydrogen bonding, van der Waals forces, self-contact energies, lipophilic interactions, and solvation effects. These factors were combined to calculate the binding free energy (ΔGbind) in kcal/mol, offering insights into the strength and favourability of ligand-protein interactions—a standard practice in drug discovery and molecular dynamics research. Using the MD simulation trajectory, we calculated the binding free energy and other contributing energies via MM-GBSA for the ligand complexes. As shown in Table 13, the average binding free energy of the NS5 protein with the standard antiviral drug dasabuvir complex (−52.28 ± 2.91 kcal/mol) is slightly higher than that of the L2 (−46.82 ± 4.31 kcal/mol) and L3 (−50.72 ± 6.36 kcal/mol) complexes by −5.46 and −2.56 kcal/mol, respectively. Whereas the NS5-L4 complex shared a higher binding energy of −57.03 ± 4.31, which is higher than the standard FDA-approved drug dasabuvir by −5.75 kcal/mol. This suggests that the NS5 protein-L4 complex is slightly more stable compared to the other ligand complexes. Among the screened ligands, L4 exhibited the highest binding energy.

**Table 13:** Binding free energy components calculated by MM-GBSA for L1, L2, L3, & L4 complex.

Energies (kcal/mol)	L1 complex	L2 complex	L3 complex	L4 Complex
ΔG_bind_	−52.28 ± 2.91	−46.82 ± 4.31	−50.72 ± 6.36	−57.03 ± 4.31
ΔG_bind_Lipo	11.89 ± 0.75	−12.53 ± 1.24	−9.31 ± 1.48	−22.11 ± 0.99
ΔG_bind_vdW	−50.69 ± 1.74	−40.12 ± 2.56	−42.81 ± 3.16	−55.53 ± 2.29
ΔG_bind_Coulomb	10.22 ± 2.42	−15.01 ± 4.09	123.13 ± 14.50	−13.17 ± 3.35
ΔG_bind_H_bond_	−1.60 ± 0.28	−1.34 ± 0.96	−1.26 ± 0.30	−1.16 ± 0.40
ΔG_bind_SolvGB	21.84 ± 1.74	23.023 ± 3.19	−119.46 ± 12.97	28.81 ± 3.16
ΔG_bind_Covalent	2.44 ± 1.20	1.69 ± 0.96	2.51 ± 1.24	7.27 ± 1.37

## 4. Discussion

Given the limited efficacy of the vaccine in combating KFDV, the public health response is weakened, making it less effective in addressing the situation [15,39,119,120]. The rising number of KFDV cases and the expanding endemic areas in India highlight the need for an interdisciplinary approach to develop antiviral drug candidates for KFDV [5,121]. This study aimed to inhibit the function of the NS5 protein, which is involved in the synthesis and methylation of viral RNA, by identifying a novel and effective antiviral drug candidate [122–124] against KFDV using a de novo design and pharmacophore-based screening approach. In this study, we obtained the NS5 protein sequence (2514–3416 of 903 amino acids) from the UniProt database (UniProt ID D7RF80.1) and conducted a sequence analysis. Multiple sequence alignment (MSA) showed that key structural motifs for RNA synthesis are conserved. Specifically, amino acids Asp535, Asp664, and Asp665 from motifs C & A are essential for coordinating divalent metal ions for nucleotide polymerization and are part of the conserved ligand binding sites. Additionally, Trp796, which aids in initiating de novo RNA synthesis by stacking the first nucleotide, is also conserved. The methylation domain interacts with the fingers subdomain of the RNA polymerase through a hydrophobic network involving residues Pro113, Leu115, and Trp121 from MTase domain, and Phe350, Phe466, and Pro584 from RdRp domain, all of which are conserved [125–128] and are highlighted with red arrow mark in S1 Fig.

The lack of a 3D structure for the NS5 protein necessitated the use of Robetta, Swiss Model, and I-Tasser servers to establish its 3D structure. The model developed from Robetta was chosen based on the structural validation procedure and then underwent global energy minimization by using Desmond software. The globally minimized NS5 protein structure with potential energy of −416966.82 kcal/mol was retrieved at 49.58 ns after simulating NS5 protein for 200 ns. The globally minimized structure exhibited a decrease in ERRAT score from 96.629 to 90.40, indicating Robetta model is of high quality [129,130]. The globally minimized structure was further used to determine active site. This globally minimized stable protein structure was crucial for the downstream computational tasks, especially for accurate binding site identification and virtual screening. Prediction of protein-ligand binding sites is crucial for annotating protein activity and discovering drugs. By integrating these validated structural insights with subsequent binding site prediction, the methodology ensured that the identified pocket accurately reflected biologically relevant interaction sites, thereby strengthening the rational design of targeted inhibitors.

To ensure the rational design of inhibitors that effectively target functionally relevant regions of the NS5 protein, accurate identification of the active site is essential following structural refinement. Various techniques for active site prediction include sequence-based, structure-based, and template-based methods. Among these, we employed template-based and structure-based methods, as they have demonstrated superior accuracy and the ability to reveal detailed insights into ligand-binding mechanisms. This is achieved by examining existing templates for proteins that have high-quality structures available [131–133]. Three different methods like COACH-D, CASTp and PrankWeb server were employed for determining the active site of NS5 protein. The amino acid common in two of the predicted binding site were selected as active site residues of NS5 protein. The active site of the protein contains the following amino acids Leu513, Leu516, Asn612, Ser662, Gly663, Asp664, Asp665, Phe709, Cys710, Ser711, His712, Arg730, Glu734, Arg738, Tyr759, Met762, Thr794, Thr795, Trp796, Ser797, Ile798, Ala800, and Trp804. The coordinates of the NS5 protein (X:0.543719, Y:12.760809, Z:- 11.958161) were evaluated using Discovery Studio software. The active site residue identified were located within polymerase motif (A-G) of NS5 protein (residues 273–903 aa). These residues are essential for polymerase function, RNA synthesis and viral replication [134]. The amino acid residue of active site Gly663, Asp664, and Asp665 were part of motif C, which are involved in catalytic activity. The amino acid Arg730, Glu734, and Arg738 are part of motif E called as primer-grip motif, which is important for stabilization of RNA substrate [135]. The identification of well defined active site strengthen its potential as a target for antiviral development [136]. These coordinates were then utilized to discover ligands based on the Pyridazinone inhibitor template (PubChem ID: 135566439).

A total of 1523 compounds were identified using four different methods: de-novo designing by eLea3D (51 drugs), template-based design by LigDream (141 drugs), pharmacophore screening by pharmit 1083 compounds (659 from the COCONUT database, and 424 from ZINC database), and ligand screening by the PubChem database (242 drugs). Virtual screening of all identified compounds was conducted using PyRx and compared with the standard FDA-approved antiviral drugs. After primary and secondary virtual screening, a total of 34 compounds with 6 FDA approved antiviral drugs were considered for molecular docking by AutoDock 4.0. Further 11 ligands with having highest binding affinities were considered for ADMET analysis. Toxicity by ProtTox II revelead that the compound Dasabuvir (L1), CNPO331352.1(L2), ZINC00103114410 (L3), CNPO202263.1(L4), ZINC005214287430 (L5), 136046538 (L6), and CNP0272687.1(L7) were taken for ADME analysis.

In order to determine the suitability of these compounds as drugs, an ADMET analysis of the identified molecule was conducted, using dasabuvir as a reference [137]. The Lipinski rule of five, which states that a compound would have poor absorption if it violates more than two of the amounts listed in S8 Table [138], is widely recognized and valuable in the early stages of therapeutic research. All of the chosen compounds are considered to satisfy Lipinski’s criterion except L2 and can be classified as drug-like compounds. With the exception of L5, all the compounds exhibit a modest level of synthetic accessibility, similar to the reference compound [139]. The ADMET properties of all the selected compounds, are in accordance with the standard dasabuvir medication. Therefore, these compounds have been chosen for geometry optimization.

The selected compound underwent geometry optimization using density functional theory (DFT) with Orca 4.2.1. The FMO method was used to compute the energy difference between the HOMO and the LUMO in order to assess the chemical reactivity of the compounds. All compounds, except L6, had an energy gap of more than 3.50eV. This indicates a high level of kinetic stability and minimal reactivity, which correlates with the compounds’ bioactivity [140,141]. The compounds that underwent geometry optimization were subjected to re-docking using Auto Dock 4.0 with the NS5 protein. This resulted in a drop in the affinity of all the compounds. The decrease and increase in dinding affinity of the geometric optimized ligands may be attributed to structural and conformational changes of ligands. This structural changes affect the loss or increase of hydrogen bonds, altered electrostatic interactions [142,141]. The magnitude of the energy difference between the HOMO and the LUMO is directly correlated with the level of excitability of a molecule. A smaller energy gap indicates that electronic excitation between the HOMO and LUMO is more easily achieved, whereas a larger energy gap implies the opposite. Compounds L5 displayed lower chemical reactivity compared to the other compounds, as evidenced by the docking score [143,144].

Following the completion of geometry optimization, molecular docking was performed to further assess the binding affinities and interaction profiles of the selected ligands against the NS5 protein. This step was critical to validate whether the shortlisted compounds could establish favorable and stable interactions within the previously identified and biologically relevant binding pocket. The docking studies, carried out using AutoDock 4.0, focused on seven ligands, Dasabuvir (reference) and six lead compounds (L2–L7) identified through a rigorous multi-method screening pipeline integrating de novo, template-based, and pharmacophore-driven strategies.

The docking results revealed that all compounds exhibited favorable binding energies and consistent interaction profiles with the active site residues. Notably, the majority of the ligands showed strong interactions with catalytically essential residues such as Asp664 and Asp665 (motif C), which are known to coordinate divalent metal ions during RNA polymerization. In addition, the ligands frequently interacted with residues Trp796, Ile798, and Thr794, involved in RNA primer stacking and substrate stabilization. These interactions were consistent across multiple compounds, underscoring the reliability of the binding site predicted earlier and the relevance of these residues in inhibitor design. Hydrogen bonds are typically essential for maintaining the stability of ligands and enabling their interaction with biological targets. The identified compounds exhibited equivalent or greater traditional hydrogen bonding compared to the reference molecule, suggesting a more effective contact with higher affinity [145–147]. Overall, the docking results support the hypothesis that the selected ligands can effectively bind to functionally critical residues of the NS5 polymerase domain. This strengthens their candidacy as potential antiviral agents against KFDV. Moreover, the structural congruence between the docking interactions and conserved active site residues provides mechanistic insight into how these compounds may inhibit the enzymatic function of NS5, thereby disrupting viral RNA synthesis and replication.

In order to conduct in-silico analysis and determine the stability of the protein-ligand complex, it is necessary to validate molecular docking using MD simulations for a minimum of 100 nanoseconds [148–150]. The re-docking complex structure for the NS5 protein- ligand L2, L3, L4 & dasabuvir (L1) complex was subjected to MD simulation to evaluate the stability of the compounds within the protein’s binding region. The simulated trajectories lasting 200 nanoseconds, which encompassed measurements of RMSD, RMSF, protein-ligand contact mapping, and ligand characteristics [4,151], were subjected to analysis using the Desmond software. Both the NS5 protein-L3 and NS5 protein-L4 complexes displayed stable conformations in the present investigation. During the 200 ns simulation, the ligands in the protein-ligand complexes made efforts to investigate various binding positions while ensuring stability within the specified active site. Previous studies have revealed similar findings, indicating that ligands actively search for stable binding sites during MD simulations [4,148,152,153].

In addition, an assessment of the RMSF was performed for both the protein and the ligand using an all-atom MD simulation. The RMSF values of terminal amino acids were often greater than those of other residues, with the optimal values being below 2 Å. The amino acid residues that interact with the protein should also have RMSF values below 2 Å. During the 200 nanosecond MD simulation, all ligands consistently maintained substantial connections with the protein. The observation was made regarding the association between ligand RMSD, ligand RMSF, and protein-ligand interactions. In addition, the MM-GBSA method was used to calculate the binding free energies of the ligand-protein complexes in MD simulations. The NS5 protein exhibits an average binding free energy of −52.28 ± 2.91 kcal/mol with the L1 complex, −46.82 ± 4.31 kcal/mol with the L2 complex, −50.72 ± 6.36 kcal/mol with the L3 complex, and −57.03 ± 4.31 kcal/mol with the L4 complex. This suggests that the L3 and L4 ligands have a comparable binding affinity to dasabuvir. Collectively, these results underscore the successful identification of two promising lead compounds, L3 and L4, through an integrated computational approach involving structure modeling, binding site prediction, virtual screening, molecular docking, ADMET profiling, and MD simulations. Their stable binding behavior and favorable binding energies highlight their potential as novel antiviral candidates against KFDV. However, further experimental validation through in vitro and in vivo studies is essential to confirm their therapeutic potential and biological efficacy [154].

## 5. Conclusion

The limitations of existing Kyasanur Forest Disease Virus (KFDV) vaccines highlight the urgent need for effective antiviral therapeutics. This study employed computational approaches to identify potential inhibitors targeting the NS5 protein, a key enzyme involved in KFDV RNA replication and methylation. Using molecular modeling, virtual screening, molecular docking, and molecular dynamics (MD) simulations, we identified promising candidates, including CNPO331352.1 (L2), ZINC00103114410 (L3), and CNPO202263.1 (L4), alongside the FDA-approved drug dasabuvir (L1). The molecular docking and MM-GBSA analyses indicated strong binding affinities, while the 200 ns MD simulations confirmed the stability of these protein-ligand complexes, particularly L3 and L4, which maintained interactions with key active site residues. Notably, these ligands exhibited comparable binding affinities to dasabuvir, suggesting their potential as novel NS5 inhibitors. To the best of our knowledge, this is the first study utilizing multiple ligand design approaches to identify NS5 inhibitors for KFDV. The computational analysis provided insights into the binding mechanisms of these ligands, further strengthening their potential as antiviral candidates. While in silico methods offer a robust platform for drug discovery, in vitro and in vivo validation is essential to confirm the efficacy of these inhibitors. Future studies will focus on experimental validation and lead optimization to enhance antiviral activity against KFDV.

## Supporting information

S1 Table NS5 protein-Transmembrane region. (DOCX)

S2 Table List of 51 compounds designed by the eLea3d serve (de novo design). (DOCX)

S3 Table List of 43 compounds designed by LigDream server by Grow mode serve. (DOCX)

S4 Table List of 100 compounds designed by LigDream server by Replace mode. (DOCX)

S5 Table Pharmacophore characteristics of Pyridazione inhibitor. (DOCX)

S6 Table Primary Virtual screening of 1520 compounds with binding energy value by PyRx software. (DOCX)

S7 Table Secondary Virtual screening of 304 compounds with binding energy value by PyRx software. (DOCX)

S8 Table Swiss ADME: Selected drugs ADME characterization. (DOCX)

S1 Fig MSA between NS5 protein of KFDV and homologous sequence. (DOCX)

S2 Fig Geometry optimization of ligands by Orca software. (DOCX)

S3 Fig FMO reflecting HOMO-LUMO and energy gap of the selected ligands. (DOCX)

S4 Fig A. RMSD and B. RMSF analysis of NS5 protein at 200 ns of replica 2. (DOCX)

S5 Fig RMSD trajectory of NS5-ligand complex at 200 ns of replica 2(A) NS5-L3 complex, (B) NS5-L5 complex, (C) NS5-L6 complex, & (D) NS5-dasabuvir complex. (DOCX)

S6 Fig RMSF trajectory of NS5-ligand complex at 200 ns of replica2(A) NS5-L1 complex, (B) NS5-L2 complex, (C) NS5-L3 complex, & (D) NS5-L4 complex. (DOCX)

S7 Fig Ligand RMSF plot of NS5-ligand complex at 200 ns of replica 1(A) L1 ligand, (B) L2 ligand, (C) L3 ligand, & (D) L4 ligand. (DOCX)

S8 Fig Ligand RMSF plot of NS5-ligand complex at 200 ns of replica 2(A) L1 ligand, (B) L2 ligand, (C) L3 ligand, & (D) L4 ligand. (DOCX)

S9 Fig NS5-ligand interaction map of replica 2(A) NS5-L1 complex, (C) NS5-L2 complex and NS5-ligand contact for more than 30% simulation time (B) NS5-L1 complex, (D) NS5-L2 complex. (DOCX)

S10 Fig NS5-ligand interaction map of replica2 (E) NS5-L3 complex, & (G) NS5-L4 complex and NS5-ligand contact for more than 30% simulation time (F) NS5-L3 complex, & (H) NS5-L4 complex. (DOCX)

S11 Fig NS5 protein-ligand interaction timeline of replica 1(A) NS5-L1 complex, (B) NS5-L2 complex. (DOCX)

S12 Fig NS5 protein-ligand interaction timeline of replica 1 (C) NS5-L3 complex, & (D) NS5-L4 complex. (DOCX)

S13 Fig NS5 protein-ligand interaction timeline of replica 2 (A) NS5-L1 complex, (B) NS5-L2 complex. (DOCX)

S14 Fig NS5 protein-ligand interaction timeline of replica 2 (C) NS5-L3 complex, & (D) NS5-L4 complex. (DOCX)

S15 Fig Binding poses of L2 ligand at the active site of NS5 protein at different time intervals during MD simulation. (DOCX)

S16 Fig Binding poses of L3 ligand at the active site of NS5 protein at different time intervals during MD simulation. (DOCX)

S17 Fig Binding poses of L4 ligand at the active site of NS5 protein at different time intervals during MD simulation. (DOCX)
