# Supplementary material for: Exploration of effective pharmacological inhibitors for NS5 protein through computational approach: A strategy to combat the neglected Kyasanur forest disease virus
Source: PLoS One. 2025 Jul 10;20(7):e0325613. doi: 10.1371/journal.pone.0325613 (PMC12244486; doi:10.1371/journal.pone.0325613)
Supplement: S1 Table — (DOCX) [file pone.0325613.s001.docx]

S1 Table. NS5 protein-Transmembrane region

|  | **Location of domain** | **Sequence position** |
| --- | --- | --- |
| NS5 protein | extracellular region | 1-903 |
|  | transmembrane | 0 |
|  | cytoplasmic region | 0 |
|  | TM Helix | 0 |
