# Supplementary material for: Exploration of effective pharmacological inhibitors for NS5 protein through computational approach: A strategy to combat the neglected Kyasanur forest disease virus
Source: PLoS One. 2025 Jul 10;20(7):e0325613. doi: 10.1371/journal.pone.0325613 (PMC12244486; doi:10.1371/journal.pone.0325613)
Supplement: S2 Table — (DOCX) [file pone.0325613.s002.docx]

S2 Table. List of 51 compounds designed by the eLea3d serve (de novo design)

| Sr.  No. | Ligand Name | Molecular Formula | Molecular Wt.(Da) | Smile format |
| --- | --- | --- | --- | --- |
|  | LIG1 | C24 H33 N2 O5 | 429.542 | c1ccccc1CC[C@H](N[C@H](C(=O)N1[C@@H](C[C@@H]2[C@@H]1CCCC2)C(=O)[O-])C)C(=O)OCC |
|  | LIG2 | C24 H33 N2 O5 | 429.529 | c1ccccc1CC[C@H](N[C@H](C(=O)N1[C@@H](C[C@@H]2[C@@H]1CCCC2)C(=O)[O-])C)C(=O)OCC |
|  | LIG3 | C24 H33 N2 O5 | 429.529 | c1ccccc1CC[C@H](N[C@H](C(=O)N1[C@@H](C[C@@H]2[C@@H]1CCCC2)C(=O)[O-])C)C(=O)OCC |
|  | LIG4 | C24 H33 N2 O5 | 429.529 | C(=O)([O-])CC[C@H](N[C@H](C(=O)N1[C@@H](C[C@@H]2[C@@H]1CCCC2)c1ccccc1)C)C(=O)OCC |
|  | LIG5 | C18 H30 N3 O5 | 368.448 | [C@H]1(C[C@@H]2[C@@H](N1C(=O)[C@@H](N[C@H](C(=O)OCC)CCC(=O)[O-])C)CCCC2)N |
|  | LIG6 | C18 H30 N3 O5 | 368.448 | [C@H]1(C[C@@H]2[C@@H](N1C(=O)[C@@H](N[C@H](C(=O)OCC)CCC(=O)[O-])C)CCCC2)N |
|  | LIG7 | C18 H30 N3 O5 | 368.448 | [C@H]1(C[C@@H]2[C@@H](N1C(=O)[C@@H](N[C@H](C(=O)OCC)CCC(=O)[O-])C)CCCC2)N |
|  | LIG8 | C25 H45 N5 O3 | 463.656 | C(=O)([C@@H](N[C@H](C(=O)OCC)CC[C@@H]1C[C@@H]2[C@@H](N1N)CCCC2)C)N1[C@@H](C[C@@H]2[C@@H]1CCCC2)N |
|  | LIG9 | C25 H45 N5 O3 | 463.656 | C(=O)([C@@H](N[C@H](C(=O)OCC)CC[C@@H]1C[C@@H]2[C@@H](N1N)CCCC2)C)N1[C@@H](C[C@@H]2[C@@H]1CCCC2)N |
|  | LIG10 | C25 H45 N5 O3 | 463.656 | C(=O)([C@@H](N[C@H](C(=O)OCC)CC[C@@H]1C[C@@H]2[C@@H](N1N)CCCC2)C)N1[C@@H](C[C@@H]2[C@@H]1CCCC2)N |
|  | LIG11 | C25 H45 N5 O3 | 463.656 | [C@H]1(C[C@@H]2[C@@H](N1C(=O)[C@@H](N[C@H](C(=O)OCC)CC[C@@H]1C[C@@H]3[C@@H](N1N)CCCC3)C)CCCC2)N |
|  | LIG12 | C25 H45 N5 O3 | 463.656 | [C@H]1(C[C@@H]2[C@@H](N1C(=O)[C@@H](N[C@H](C(=O)OCC)CC[C@@H]1C[C@@H]3[C@@H](N1N)CCCC3)C)CCCC2)N |
|  | LIG13 | C25 H45 N5 O3 | 463.656 | [C@H]1(C[C@@H]2[C@@H](N1C(=O)[C@@H](N[C@H](C(=O)OCC)CC[C@@H]1C[C@@H]3[C@@H](N1N)CCCC3)C)CCCC2)N |
|  | LIG14 | C25 H45 N5 O3 | 463.656 | [C@H]1(C[C@@H]2[C@@H](N1C(=O)[C@@H](N[C@H](C(=O)OCC)CC[C@@H]1C[C@@H]3[C@@H](N1N)CCCC3)C)CCCC2)N |
|  | LIG15 | C25 H45 N5 O3 | 463.656 | [C@H]1(C[C@@H]2[C@@H](N1C(=O)[C@@H](N[C@H](C(=O)OCC)CC[C@@H]1C[C@@H]3[C@@H](N1N)CCCC3)C)CCCC2)N |
|  | LIG16 | C25 H45 N5 O3 | 463.656 | [C@H]1(C[C@@H]2[C@@H](N1C(=O)[C@@H](N[C@H](C(=O)OCC)CC[C@@H]1C[C@@H]3[C@@H](N1N)CCCC3)C)CCCC2)N |
|  | LIG17 | C25 H45 N5 O3 | 463.656 | [C@H]1(C[C@@H]2[C@@H](N1C(=O)[C@@H](N[C@H](C(=O)OCC)CC[C@@H]1C[C@@H]3[C@@H](N1N)CCCC3)C)CCCC2)N |
|  | LIG18 | C25 H45 N5 O3 | 463.656 | [C@H]1(C[C@@H]2[C@@H](N1C(=O)[C@@H](N[C@H](C(=O)OCC)CC[C@@H]1C[C@@H]3[C@@H](N1N)CCCC3)C)CCCC2)N |
|  | LIG19 | C25 H45 N5 O3 | 463.656 | [C@H]1(C[C@@H]2[C@@H](N1C(=O)[C@@H](N[C@H](C(=O)OCC)CC[C@@H]1C[C@@H]3[C@@H](N1N)CCCC3)C)CCCC2)N |
|  | LIG20 | C25 H45 N5 O3 | 463.656 | [C@H]1(C[C@@H]2[C@@H](N1C(=O)[C@@H](N[C@H](C(=O)OCC)CC[C@@H]1C[C@@H]3[C@@H](N1N)CCCC3)C)CCCC2)N |
|  | LIG21 | C25 H44 N4 O3 | 448.642 | C1C[C@@H]2[C@@H](N1C(=O)[C@@H](N[C@H](C(=O)OCC)CC[C@@H]1C[C@@H]3[C@@H](N1N)CCCC3)C)CCCC2 |
|  | LIG22 | C25 H44 N4 O3 | 448.642 | [C@H]1(C[C@@H]2[C@@H](N1N)CCCC2)CC[C@H](N[C@H](C(=O)N1CC[C@@H]2[C@@H]1CCCC2)C)C(=O)OCC |
|  | LIG23 | C25 H44 N4 O3 | 448.642 | [C@H]1(C[C@@H]2[C@@H](N1N)CCCC2)CC[C@H](N[C@H](C(=O)N1CC[C@@H]2[C@@H]1CCCC2)C)C(=O)OCC |
|  | LIG24 | C23 H44 N4 O4 | 440.62 | [C@H]1(C[C@@H]2[C@@H](N1N)CCCC2)CC[C@H](N[C@H](C(=O)[C@H](CNC(C)(C)C)O)C)C(=O)OCC |
|  | LIG25 | C23 H44 N4 O4 | 440.62 | [C@H]1(C[C@@H]2[C@@H](N1N)CCCC2)CC[C@H](N[C@H](C(=O)[C@H](CNC(C)(C)C)O)C)C(=O)OCC |
|  | LIG26 | C23 H44 N4 O4 | 440.62 | [C@H]1(C[C@@H]2[C@@H](N1N)CCCC2)CC[C@H](N[C@H](C(=O)[C@H](CNC(C)(C)C)O)C)C(=O)OCC |
|  | LIG27 | C23 H44 N4 O4 | 440.62 | [C@H]1(C[C@@H]2[C@@H](N1N)CCCC2)CC[C@H](N[C@H](C(=O)[C@H](CNC(C)(C)C)O)C)C(=O)OCC |
|  | LIG28 | C23 H44 N4 O4 | 440.62 | [C@H]1(C[C@@H]2[C@@H](N1N)CCCC2)CC[C@H](N[C@H](C(=O)[C@H](CNC(C)(C)C)O)C)C(=O)OCC |
|  | LIG29 | C23 H44 N4 O4 | 440.62 | [C@H]1(C[C@@H]2[C@@H](N1N)CCCC2)CC[C@H](N[C@H](C(=O)[C@H](CNC(C)(C)C)O)C)C(=O)OCC |
|  | LIG30 | C22 H35 N5 O4 | 433.544 | n1c(n(c2c1cccc2)[C@H](CNC(C)(C)C)O)CC[C@H](N[C@H](C(=O)N)C)C(=O)OCC |
|  | LIG31 | C23 H43 N3 O5 | 441.605 | [C@H]1(C[C@@H]2[C@@H](N1O)CCCC2)CC[C@H](N[C@H](C(=O)[C@H](CNC(C)(C)C)O)C)C(=O)OCC |
|  | LIG32 | C22 H35 N5 O4 | 433.544 | n1c(n(c2c1cccc2)[C@H](CNC(C)(C)C)O)CC[C@H](N[C@H](C(=O)N)C)C(=O)OCC |
|  | LIG33 | C24 H35 N5 O4 | 457.566 | n1c(n(c2c1ccc(c2)C#C)[C@H](CNC(C)(C)C)O)CC[C@H](N[C@H](C(=O)N)C)C(=O)OCC |
|  | LIG34 | C24 H35 N5 O4 | 457.566 | n1c(n(c2c1ccc(c2)C#C)[C@H](CNC(C)(C)C)O)CC[C@H](N[C@H](C(=O)N)C)C(=O)OCC |
|  | LIG35 | C24 H35 N5 O4 | 457.566 | n1c(n(c2c1ccc(c2)C#C)[C@H](CNC(C)(C)C)O)CC[C@H](N[C@H](C(=O)N)C)C(=O)OCC |
|  | LIG36 | C24 H35 N5 O4 | 457.566 | n1c(n(c2c1ccc(c2)C#C)[C@H](CNC(C)(C)C)O)CC[C@H](N[C@H](C(=O)N)C)C(=O)OCC |
|  | LIG37 | C24 H35 N5 O4 | 457.566 | n1c(n(c2c1ccc(c2)C#C)[C@H](CNC(C)(C)C)O)CC[C@H](N[C@H](C(=O)N)C)C(=O)OCC |
|  | LIG38 | C24 H35 N5 O4 | 457.566 | n1c(n(c2c1ccc(c2)C#C)[C@H](CNC(C)(C)C)O)CC[C@H](N[C@H](C(=O)N)C)C(=O)OCC |
|  | LIG39 | C24 H35 N5 O4 | 457.566 | n1c(n(c2c1ccc(c2)C#C)[C@H](CNC(C)(C)C)O)CC[C@H](N[C@H](C(=O)N)C)C(=O)OCC |
|  | LIG40 | C24 H35 N5 O4 | 457.566 | n1c(n(c2c1ccc(c2)C#C)[C@H](CNC(C)(C)C)O)CC[C@H](N[C@H](C(=O)N)C)C(=O)OCC |
|  | LIG41 | C24 H35 N5 O4 | 457.566 | C(=O)([C@@H](N[C@H](C(=O)OCC)CCc1nc2c(n1[C@H](CNC(C)(C)C)O)cc(cc2)N)C)C#C |
|  | LIG42 | C24 H35 N5 O4 | 457.566 | C(=O)([C@@H](N[C@H](C(=O)OCC)CCc1nc2c(n1[C@H](CNC(C)(C)C)O)cc(cc2)C#C)C)N |
|  | LIG43 | C24 H35 N5 O4 | 457.566 | C(=O)([C@@H](N[C@H](C(=O)OCC)CCc1nc2c(n1[C@H](CNC(C)(C)C)O)cc(cc2)C#C)C)N |
|  | LIG44 | C24 H35 N5 O4 | 457.566 | C(=O)([C@@H](N[C@H](C(=O)OCC)CCc1nc2c(n1[C@H](CNC(C)(C)C)O)cc(cc2)C#C)C)N |
|  | LIG45 | C24 H35 N5 O4 | 457.566 | C(=O)([C@@H](N[C@H](C(=O)OCC)CCc1nc2c(n1[C@H](CNC(C)(C)C)O)cc(cc2)C#C)C)N |
|  | LIG48 | C24 H35 N5 O4 | 457.566 | C(=O)([C@@H](N[C@H](C(=O)OCC)CCc1nc2c(n1[C@H](CNC(C)(C)C)O)cc(cc2)C#C)C)N |
|  | LIG47 | C24 H35 N5 O4 | 457.566 | C(=O)([C@@H](N[C@H](C(=O)OCC)CCc1nc2c(n1[C@H](CNC(C)(C)C)O)cc(cc2)C#C)C)N |
|  | LIG48 | C24 H35 N5 O4 | 457.566 | C(=O)([C@@H](N[C@H](C(=O)OCC)CCc1nc2c(n1[C@H](CNC(C)(C)C)O)cc(cc2)C#C)C)N |
|  | LIG49 | C24 H35 N5 O4 | 457.566 | C(=O)([C@@H](N[C@H](C(=O)OCC)CCc1nc2c(n1[C@H](CNC(C)(C)C)O)cc(cc2)C#C)C)N |
|  | LIG50 | C24 H35 N5 O4 | 457.566 | C(=O)([C@@H](N[C@H](C(=O)OCC)CCc1nc2c(n1[C@H](CNC(C)(C)C)O)cc(cc2)C#C)C)N |
|  | LIG51 | C24 H35 N5 O4 | 457.566 | C(=O)([C@@H](N[C@H](C(=O)OCC)CCc1nc2c(n1[C@H](CNC(C)(C)C)O)cc(cc2)C#C)C)N |
