# Supplementary material for: Exploration of effective pharmacological inhibitors for NS5 protein through computational approach: A strategy to combat the neglected Kyasanur forest disease virus
Source: PLoS One. 2025 Jul 10;20(7):e0325613. doi: 10.1371/journal.pone.0325613 (PMC12244486; doi:10.1371/journal.pone.0325613)
Supplement: S3 Table — (DOCX) [file pone.0325613.s003.docx]

S3 Table. List of 43 compounds designed by LigDream server by Grow mode serve

| **Sr.**  **No.** | **Ligand Name** | **Molecular Formula** | **Molecular Wt.(Da)** | **Smile format** |
| --- | --- | --- | --- | --- |
|  | LIGG1 | C22H32N6O6S2 | 540.668 | CC(C)CCn1nc(N2CCCC2)c(O)c(C2=N[S+2](O)(O)c3cc(N(C)S(C)(=O)=O)ccc3N2)c1=O |
|  | LIGG2 | C22H32N6O6S2 | 540.668 | COc1c(N2CCCC2)nn(CCC(C)C)c(=O)c1C1=N[S+2](O)(O)c2cc(NS(C)(=O)=O)ccc2N1 |
|  | LIGG3 | C23H34N6O6S2 | 554.695 | CCOc1c(N2CCCC2)nn(CCC(C)C)c(=O)c1C1=N[S+2](O)(O)c2cc(NS(C)(=O)=O)ccc2N1 |
|  | LIGG4 | C21H30N6O7S2 | 542.64 | CC(C)CCn1nc(N2CCCC2)c(OO)c(C2=N[S+2](O)(O)c3cc(NS(C)(=O)=O)ccc3N2)c1=O |
|  | LIGG5 | C21H29BrN6O6S2 | 605.537 | CC(C)CCn1nc(N2CCCC2)c(O)c(C2=N[S+2](O)(O)c3cc(NS(C)(=O)=O)c(Br)cc3N2)c1=O |
|  | LIGG6 | C22H32N6O6S2 | 540.668 | Cc1cc2c(cc1NS(C)(=O)=O)[S+2](O)(O)N=C(c1c(O)c(N3CCCC3)nn(CCC(C)C)c1=O)N2 |
|  | LIGG7 | C21H29ClN6O6S2 | 561.086 | CC(C)CCn1nc(N2CCCC2)c(O)c(C2=N[S+2](O)(O)c3cc(NS(C)(=O)=O)c(Cl)cc3N2)c1=O |
|  | LIGG8 | C22H32N6O7S2 | 556.667 | COc1cc2c(cc1NS(C)(=O)=O)[S+2](O)(O)N=C(c1c(O)c(N3CCCC3)nn(CCC(C)C)c1=O)N2 |
|  | LIGG9 | C21H29FN6O6S2 | 544.631 | CC(C)CCn1nc(N2CCCC2)c(O)c(C2=N[S+2](O)(O)c3cc(NS(C)(=O)=O)c(F)cc3N2)c1=O |
|  | LIGG11 | C21H31N7O6S2 | 541.656 | CC(C)CCn1nc(N2CCCC2)c(O)c(C2=N[S+2](O)(O)c3cc(NS(C)(=O)=O)c(N)cc3N2)c1=O |
|  | LIGG12 | C22H29N7O6S2 | 551.651 | CC(C)CCn1nc(N2CCCC2)c(O)c(C2=N[S+2](O)(O)c3cc(NS(C)(=O)=O)c(C#N)cc3N2)c1=O |
|  | LIGG13 | C21H30N6O7S2 | 542.64 | CC(C)CCn1nc(N2CCCC2)c(O)c(C2=N[S+2](O)(O)c3cc(NS(C)(=O)=O)c(O)cc3N2)c1=O |
|  | LIGG14 | C22H32N6O7S2 | 556.667 | CC(C)CCn1nc(N2CCCC2)c(O)c(C2=N[S+2](O)(O)c3cc(NS(C)(=O)=O)c(CO)cc3N2)c1=O |
|  | LIGG15 | C21H29BrN6O6S2 | 605.537 | CC(C)CCn1nc(N2CCCC2)c(O)c(C2=N[S+2](O)(O)c3cc(NS(C)(=O)=O)cc(Br)c3N2)c1=O |
|  | LIGG16 | C22H32N6O6S2 | 540.668 | Cc1cc(NS(C)(=O)=O)cc2c1NC(c1c(O)c(N3CCCC3)nn(CCC(C)C)c1=O)=N[S+2]2(O)O |
|  | LIGG17 | C23H34N6O6S2 | 554.695 | CCc1cc(NS(C)(=O)=O)cc2c1NC(c1c(O)c(N3CCCC3)nn(CCC(C)C)c1=O)=N[S+2]2(O)O |
|  | LIGG18 | C21H29ClN6O6S2 | 561.086 | CC(C)CCn1nc(N2CCCC2)c(O)c(C2=N[S+2](O)(O)c3cc(NS(C)(=O)=O)cc(Cl)c3N2)c1=O |
|  | LIGG19 | C22H32N6O7S2 | 556.667 | COc1cc(NS(C)(=O)=O)cc2c1NC(c1c(O)c(N3CCCC3)nn(CCC(C)C)c1=O)=N[S+2]2(O)O |
|  | LIGG20 | C21H29FN6O6S2 | 544.631 | CC(C)CCn1nc(N2CCCC2)c(O)c(C2=N[S+2](O)(O)c3cc(NS(C)(=O)=O)cc(F)c3N2)c1=O |
|  | LIGG22 | C22H29N7O6S2 | 551.651 | CC(C)CCn1nc(N2CCCC2)c(O)c(C2=N[S+2](O)(O)c3cc(NS(C)(=O)=O)cc(C#N)c3N2)c1=O |
|  | LIGG23 | C21H30N6O7S2 | 542.64 | CC(C)CCn1nc(N2CCCC2)c(O)c(C2=N[S+2](O)(O)c3cc(NS(C)(=O)=O)cc(O)c3N2)c1=O |
|  | LIGG24 | C22H32N6O6S2 | 540.668 | CCS(=O)(=O)Nc1ccc2c(c1)[S+2](O)(O)N=C(c1c(O)c(N3CCCC3)nn(CCC(C)C)c1=O)N2 |
|  | LIGG25 | C23H34N6O6S2 | 554.695 | CCCS(=O)(=O)Nc1ccc2c(c1)[S+2](O)(O)N=C(c1c(O)c(N3CCCC3)nn(CCC(C)C)c1=O)N2 |
|  | LIGG27 | C21H29FN6O6S2 | 544.631 | CC(C)CCn1nc(N2CCCC2)c(O)c(C2=N[S+2](O)(O)c3cc(NS(=O)(=O)CF)ccc3N2)c1=O |
|  | LIGG29 | C22H32N6O6S2 | 540.668 | CCC(C)CCn1nc(N2CCCC2)c(O)c(C2=N[S+2](O)(O)c3cc(NS(C)(=O)=O)ccc3N2)c1=O |
|  | LIGG30 | C23H34N6O6S2 | 554.695 | CCCC(C)CCn1nc(N2CCCC2)c(O)c(C2=N[S+2](O)(O)c3cc(NS(C)(=O)=O)ccc3N2)c1=O |
|  | LIGG31 | C22H33N7O6S2 | 555.683 | CNCC(C)CCn1nc(N2CCCC2)c(O)c(C2=N[S+2](O)(O)c3cc(NS(C)(=O)=O)ccc3N2)c1=O |
|  | LIGG32 | C21H31N7O6S2 | 541.656 | CC(CN)CCn1nc(N2CCCC2)c(O)c(C2=N[S+2](O)(O)c3cc(NS(C)(=O)=O)ccc3N2)c1=O |
|  | LIGG33 | C21H30N6O7S2 | 542.64 | CC(CO)CCn1nc(N2CCCC2)c(O)c(C2=N[S+2](O)(O)c3cc(NS(C)(=O)=O)ccc3N2)c1=O |
|  | LIGG34 | C22H30N6O7S2 | 554.651 | CC(CC=O)CCn1nc(N2CCCC2)c(O)c(C2=N[S+2](O)(O)c3cc(NS(C)(=O)=O)ccc3N2)c1=O |
|  | LIGG35 | C22H32N6O7S2 | 556.667 | CC(CCO)CCn1nc(N2CCCC2)c(O)c(C2=N[S+2](O)(O)c3cc(NS(C)(=O)=O)ccc3N2)c1=O |
|  | LIGG36 | C21H30N6O6S3 | 558.708 | CC(CS)CCn1nc(N2CCCC2)c(O)c(C2=N[S+2](O)(O)c3cc(NS(C)(=O)=O)ccc3N2)c1=O |
|  | LIGG37 | C22H32N6O6S2 | 540.668 | CC(C)(C)CCn1nc(N2CCCC2)c(O)c(C2=N[S+2](O)(O)c3cc(NS(C)(=O)=O)ccc3N2)c1=O |
|  | LIGG38 | C22H32N6O7S2 | 556.667 | COC(C)(C)CCn1nc(N2CCCC2)c(O)c(C2=N[S+2](O)(O)c3cc(NS(C)(=O)=O)ccc3N2)c1=O |
|  | LIGG39 | C21H31N7O6S2 | 541.656 | CC(C)(N)CCn1nc(N2CCCC2)c(O)c(C2=N[S+2](O)(O)c3cc(NS(C)(=O)=O)ccc3N2)c1=O |
|  | LIGG40 | C22H33N7O6S2 | 555.683 | CC(C)(CN)CCn1nc(N2CCCC2)c(O)c(C2=N[S+2](O)(O)c3cc(NS(C)(=O)=O)ccc3N2)c1=O |
|  | LIGG41 | C21H30N6O7S2 | 542.64 | CC(C)(O)CCn1nc(N2CCCC2)c(O)c(C2=N[S+2](O)(O)c3cc(NS(C)(=O)=O)ccc3N2)c1=O |
|  | LIGG42 | C22H32N6O7S2 | 556.667 | CC(C)(CO)CCn1nc(N2CCCC2)c(O)c(C2=N[S+2](O)(O)c3cc(NS(C)(=O)=O)ccc3N2)c1=O |
|  | LIGG43 | C21H30N6O6S3 | 558.708 | CC(C)(S)CCn1nc(N2CCCC2)c(O)c(C2=N[S+2](O)(O)c3cc(NS(C)(=O)=O)ccc3N2)c1=O |
|  | LIGG44 | C21H30N6O7S2 | 542.64 | CC(C)CCn1nc(N2CCC(O)C2)c(O)c(C2=N[S+2](O)(O)c3cc(NS(C)(=O)=O)ccc3N2)c1=O |
|  | LIGG1 | C22H32N6O6S2 | 540.668 | CC(C)CCn1nc(N2CCCC2)c(O)c(C2=N[S+2](O)(O)c3cc(N(C)S(C)(=O)=O)ccc3N2)c1=O |
|  | LIGG2 | C22H32N6O6S2 | 540.668 | COc1c(N2CCCC2)nn(CCC(C)C)c(=O)c1C1=N[S+2](O)(O)c2cc(NS(C)(=O)=O)ccc2N1 |
|  | LIGG3 | C23H34N6O6S2 | 554.695 | CCOc1c(N2CCCC2)nn(CCC(C)C)c(=O)c1C1=N[S+2](O)(O)c2cc(NS(C)(=O)=O)ccc2N1 |
|  | LIGG4 | C21H30N6O7S2 | 542.64 | CC(C)CCn1nc(N2CCCC2)c(OO)c(C2=N[S+2](O)(O)c3cc(NS(C)(=O)=O)ccc3N2)c1=O |
