# Supplementary material for: Exploration of effective pharmacological inhibitors for NS5 protein through computational approach: A strategy to combat the neglected Kyasanur forest disease virus
Source: PLoS One. 2025 Jul 10;20(7):e0325613. doi: 10.1371/journal.pone.0325613 (PMC12244486; doi:10.1371/journal.pone.0325613)
Supplement: S4 Table — (DOCX) [file pone.0325613.s004.docx]

S4 Table. List of 100 compounds designed by LigDream server by Replace mode

| **Sr.**  **No.** | **Ligand Name** | **Molecular Formula** | **Molecular Wt.(Da)** | **Smile format** |
| --- | --- | --- | --- | --- |
|  | LIGR1 | C25H36N6O4S | 516.67 | CCC1(N)CC1c1ccc2c(c1)[S+2](O)(O)N=C(c1c(O)c(N3CCCC3)nn(CCC(C)C)c1=O)N2 |
|  | LIGR2 | C25H35N5O6S | 533.65 | CC(=O)C(C)C(O)c1ccc2c(c1)[S+2](O)(O)N=C(c1c(O)c(N3CCCC3)nn(CCC(C)C)c1=O)N2 |
|  | LIGR3 | C23H33N5O7S | 523.61 | CC(C)CCn1nc(N2CCCC2)c(O)c(C2=N[S+2](O)(O)c3cc(C(O)C(O)CO)ccc3N2)c1=O |
|  | LIGR4 | C21H26N8O6S | 518.56 | CC(C)CCn1nc(N2CCCC2)c(O)c(C2=N[S+2](O)(O)c3cc(-[n+]4nc(=O)o[n-]4)ccc3N2)c1=O |
|  | LIGR5 | C24H34N6O4S | 502.64 | CC(C)CCn1nc(N2CCCC2)c(O)c(C2=N[S+2](O)(O)c3cc(C4CC4CN)ccc3N2)c1=O |
|  | LIGR6 | C23H32N6O7S | 536.61 | CC(C)CCn1nc(N2CCCC2)c(O)c(C2=N[S+2](O)(O)c3cc(C(O)C(N)C(=O)O)ccc3N2)c1=O |
|  | LIGR7 | C23H34N6O5S | 506.63 | CC(C)CCn1nc(N2CCCC2)c(O)c(C2=N[S+2](O)(O)c3cc(C(O)C(C)N)ccc3N2)c1=O |
|  | LIGR8 | C24H32N6O6S | 532.62 | CC(C)CCn1nc(N2CCCC2)c(O)c(C2=N[S+2](O)(O)c3cc(C4(O)CCC(=O)N4)ccc3N2)c1=O |
|  | LIGR9 | C23H33N5O6S | 507.61 | CC(C)CCn1nc(N2CCCC2)c(O)c(C2=N[S+2](O)(O)c3cc(C(O)C(C)O)ccc3N2)c1=O |
|  | LIGR10 | C21H29N7O6S2 | 539.64 | CC(C)CCn1nc(N2CCCC2)c(O)c(C2=N[S+2](O)(O)c3cc(NC(=N)S(=O)O)ccc3N2)c1=O |
|  | LIGR11 | C23H32N6O4S | 488.61 | CC(C)CCn1nc(N2CCCC2)c(O)c(C2=N[S+2](O)(O)c3cc(C4CC4N)ccc3N2)c1=O |
|  | LIGR12 | C25H34N6O5S | 530.65 | CC(=O)NC1CC1c1ccc2c(c1)[S+2](O)(O)N=C(c1c(O)c(N3CCCC3)nn(CCC(C)C)c1=O)N2 |
|  | LIGR13 | C22H32N5O8PS | 557.57 | CC(C)CCn1nc(N2CCCC2)c(O)c(C2=N[S+2](O)(O)c3cc(C(C)(O)P(=O)(O)O)ccc3N2)c1=O |
|  | LIGR14 | C21H33N9O4S | 507.62 | CC(C)CCn1nc(N2CCCC2)c(O)c(C2=N[S+2](O)(O)c3cc(NC(N)NN)ccc3N2)c1=O |
|  | LIGR15 | C26H35N5O5S | 529.66 | C=C(C)C(O)C=Cc1ccc2c(c1)[S+2](O)(O)N=C(c1c(O)c(N3CCCC3)nn(CCC(C)C)c1=O)N2 |
|  | LIGR16 | C24H30N6O5S | 514.61 | C=C(C#N)C(O)c1ccc2c(c1)[S+2](O)(O)N=C(c1c(O)c(N3CCCC3)nn(CCC(C)C)c1=O)N2 |
|  | LIGR17 | C20H26N8O4S | 474.55 | CC(C)CCn1nc(N2CCCC2)c(O)c(C2=N[S+2](O)(O)c3cc([N-][N+]#N)ccc3N2)c1=O |
|  | LIGR18 | C24H34N6O5S | 518.64 | CC(C)CCn1nc(N2CCCC2)c(O)c(C2=N[S+2](O)(O)c3cc(CC(C)C=NO)ccc3N2)c1=O |
|  | LIGR19 | C24H29N5O7S | 531.59 | CC(C)CCn1nc(N2CCCC2)c(O)c(C2=N[S+2](O)(O)c3cc(C4C(=O)COC4=O)ccc3N2)c1=O |
|  | LIGR20 | C23H31N7O5S | 517.61 | CC(C)CCn1nc(N2CCCC2)c(O)c(C2=N[S+2](O)(O)c3cc(C4COC(N)=N4)ccc3N2)c1=O |
|  | LIGR21 | C22H31N7O5S | 505.60 | CC(C)CCn1nc(N2CCCC2)c(O)c(C2=N[S+2](O)(O)c3cc(C(O)C(=N)N)ccc3N2)c1=O |
|  | LIGR22 | C23H32N6O5S | 504.61 | CC(C)CCn1nc(N2CCCC2)c(O)c(C2=N[S+2](O)(O)c3cc(C4NCCO4)ccc3N2)c1=O |
|  | LIGR23 | C22H29N5O6S2 | 523.64 | CC(C)CCn1nc(N2CCCC2)c(O)c(C2=N[S+2](O)(O)c3cc(C(S)C(=O)O)ccc3N2)c1=O |
|  | LIGR24 | C25H37N5O6S | 535.67 | CC(C)CCn1nc(N2CCCC2)c(O)c(C2=N[S+2](O)(O)c3cc(C(C)(O)C(C)(C)O)ccc3N2)c1=O |
|  | LIGR25 | C24H36N6O5S2 | 552.72 | CC(C)CCn1nc(N2CCCC2)c(O)c(C2=N[S+2](O)(O)c3cc(NS(=O)C(C)(C)C)ccc3N2)c1=O |
|  | LIGR26 | C24H35N5O5S2 | 537.71 | CC(C)CCn1nc(N2CCCC2)c(O)c(C2=N[S+2](O)(O)c3cc(S(=O)CC(C)C)ccc3N2)c1=O |
|  | LIGR27 | C18H23N9O6S2 | 525.57 | CS(=O)(=O)Nc1ccc2c(c1)[S+2](O)(O)N=C(c1c(O)c(N3CCCC3)nn(CC[N-][N+]#N)c1=O)N2 |
|  | LIGR28 | C24H31N5O5S | 501.61 | C#CCC(O)c1ccc2c(c1)[S+2](O)(O)N=C(c1c(O)c(N3CCCC3)nn(CCC(C)C)c1=O)N2 |
|  | LIGR29 | C25H33N5O6S | 531.64 | C=CC(OC(C)=O)c1ccc2c(c1)[S+2](O)(O)N=C(c1c(O)c(N3CCCC3)nn(CCC(C)C)c1=O)N2 |
|  | LIGR30 | C25H33N5O6S | 531.64 | C=C(C(C)=O)C(O)c1ccc2c(c1)[S+2](O)(O)N=C(c1c(O)c(N3CCCC3)nn(CCC(C)C)c1=O)N2 |
|  | LIGR31 | C24H35N5O7S | 537.64 | CC(C)CCn1nc(N2CCCC2)c(O)c(C2=N[S+2](O)(O)c3cc(C(O)C(CO)CO)ccc3N2)c1=O |
|  | LIGR32 | C20H29N7O7S2 | 543.63 | CC(CCn1nc(N2CCCC2)c(O)c(C2=N[S+2](O)(O)c3cc(NS(C)(=O)=O)ccc3N2)c1=O)NO |
|  | LIGR33 | C20H28N6O6S3 | 544.68 | CC(S)CCn1nc(N2CCCC2)c(O)c(C2=N[S+2](O)(O)c3cc(NS(C)(=O)=O)ccc3N2)c1=O |
|  | LIGR34 | C23H35N7O5S | 521.64 | CC(C)CCn1nc(N2CCCC2)c(O)c(C2=N[S+2](O)(O)c3cc(C(O)CN(C)N)ccc3N2)c1=O |
|  | LIGR35 | C22H30N6O6S2 | 538.65 | C=CC(C)CCn1nc(N2CCCC2)c(O)c(C2=N[S+2](O)(O)c3cc(NS(C)(=O)=O)ccc3N2)c1=O |
|  | LIGR36 | C20H30N8O6S2 | 542.64 | CC(CCn1nc(N2CCCC2)c(O)c(C2=N[S+2](O)(O)c3cc(NS(C)(=O)=O)ccc3N2)c1=O)NN |
|  | LIGR37 | C19H25N9O6S2 | 539.60 | CS(=O)(=O)Nc1ccc2c(c1)[S+2](O)(O)N=C(c1c(O)c(N3CCCC3)nn(CCC[N-][N+]#N)c1=O)N2 |
|  | LIGR38 | C23H31N5O5S | 489.60 | C=CC(O)c1ccc2c(c1)[S+2](O)(O)N=C(c1c(O)c(N3CCCC3)nn(CCC(C)C)c1=O)N2 |
|  | LIGR39 | C21H28N6O7S2 | 540.62 | CC(C=O)CCn1nc(N2CCCC2)c(O)c(C2=N[S+2](O)(O)c3cc(NS(C)(=O)=O)ccc3N2)c1=O |
|  | LIGR40 | C22H30N8O4S | 502.60 | CC(C)CCn1nc(N2CCCC2)c(O)c(C2=N[S+2](O)(O)c3cc(CC[N-][N+]#N)ccc3N2)c1=O |
|  | LIGR41 | C21H27N7O6S2 | 537.62 | CC(C#N)CCn1nc(N2CCCC2)c(O)c(C2=N[S+2](O)(O)c3cc(NS(C)(=O)=O)ccc3N2)c1=O |
|  | LIGR42 | C21H29N5O5S2 | 495.63 | CC(C)CCn1nc(N2CCCC2)c(O)c(C2=N[S+2](O)(O)c3cc(S(C)=O)ccc3N2)c1=O |
|  | LIGR43 | C22H30N6O7S2 | 554.65 | CC(CC=O)CCn1nc(N2CCCC2)c(O)c(C2=N[S+2](O)(O)c3cc(NS(C)(=O)=O)ccc3N2)c1=O |
|  | LIGR44 | C20H27N9O6S2 | 553.63 | CS(=O)(=O)Nc1ccc2c(c1)[S+2](O)(O)N=C(c1c(O)c(N3CCCC3)nn(CCCC[N-][N+]#N)c1=O)N2 |
|  | LIGR45 | C22H33N7O5S | 507.62 | CC(C)CCn1nc(N2CCCC2)c(O)c(C2=N[S+2](O)(O)c3cc(C(O)CNN)ccc3N2)c1=O |
|  | LIGR46 | C21H30N6O5S | 478.58 | CC(C)CCn1nc(N2CCCC2)c(O)c(C2=N[S+2](O)(O)c3cc(C(N)O)ccc3N2)c1=O |
|  | LIGR47 | C23H34N8O4S | 518.64 | CC(C)CCn1nc(N2CCCC2)c(O)c(C2=N[S+2](O)(O)c3cc(C(C)NC(=N)N)ccc3N2)c1=O |
|  | LIGR48 | C22H28F3N5O5S | 531.56 | CC(C)CCn1nc(N2CCCC2)c(O)c(C2=N[S+2](O)(O)c3cc(C(O)C(F)(F)F)ccc3N2)c1=O |
|  | LIGR49 | C25H35N7O4S | 529.67 | CCC(C#N)NCc1ccc2c(c1)[S+2](O)(O)N=C(c1c(O)c(N3CCCC3)nn(CCC(C)C)c1=O)N2 |
|  | LIGR50 | C24H30N6O5S2 | 546.68 | CC(C)CCn1nc(N2CCCC2)c(O)c(C2=N[S+2](O)(O)c3cc(C(O)c4nccs4)ccc3N2)c1=O |
|  | LIGR51 | C23H34N6O5S | 506.63 | CC(C)CCn1nc(N2CCCC2)c(O)c(C2=N[S+2](O)(O)c3cc(CC(C)NO)ccc3N2)c1=O |
|  | LIGR52 | C22H33N7O6S2 | 555.68 | CC(CCn1nc(N2CCCC2)c(O)c(C2=N[S+2](O)(O)c3cc(NS(C)(=O)=O)ccc3N2)c1=O)N(C)C |
|  | LIGR53 | C23H31N5O7S | 521.60 | CC(C)CCn1nc(N2CCCC2)c(O)c(C2=N[S+2](O)(O)c3cc(C(=O)C(O)CO)ccc3N2)c1=O |
|  | LIGR54 | C25H36N6O5S | 532.67 | CC(C)CCn1nc(N2CCCC2)c(O)c(C2=N[S+2](O)(O)c3cc(C(C(N)=O)C(C)C)ccc3N2)c1=O |
|  | LIGR55 | C24H34N6O6S | 534.64 | CC(C)CCn1nc(N2CCCC2)c(O)c(C2=N[S+2](O)(O)c3cc(CC(C)(N)C(=O)O)ccc3N2)c1=O |
|  | LIGR56 | C23H29N7O6S | 531.60 | CC(C)CCn1nc(N2CCCC2)c(O)c(C2=N[S+2](O)(O)c3cc(C4NC(=O)NC4=O)ccc3N2)c1=O |
|  | LIGR57 | C21H30N6O7S2 | 542.64 | COC(C)CCn1nc(N2CCCC2)c(O)c(C2=N[S+2](O)(O)c3cc(NS(C)(=O)=O)ccc3N2)c1=O |
|  | LIGR58 | C21H31N7O6S2 | 541.66 | CC(CN)CCn1nc(N2CCCC2)c(O)c(C2=N[S+2](O)(O)c3cc(NS(C)(=O)=O)ccc3N2)c1=O |
|  | LIGR59 | C21H29N7O7S2 | 555.64 | CC(CCn1nc(N2CCCC2)c(O)c(C2=N[S+2](O)(O)c3cc(NS(C)(=O)=O)ccc3N2)c1=O)C(N)=O |
|  | LIGR60 | C22H29N9O5S | 531.60 | CC(C)CCn1nc(N2CCCC2)c(O)c(C2=N[S+2](O)(O)c3cc(NC(=O)C[N-][N+]#N)ccc3N2)c1=O |
|  | LIGR61 | C23H32N6O6S2 | 552.68 | CC(C)CCn1nc(N2CCCC2)c(O)c(C2=N[S+2](O)(O)c3cc(NC(CS)C(=O)O)ccc3N2)c1=O |
|  | LIGR62 | C23H32N6O6S2 | 552.68 | CC(C)CCn1nc(N2CCCC2)c(O)c(C2=N[S+2](O)(O)c3cc(SCC(N)C(=O)O)ccc3N2)c1=O |
|  | LIGR63 | C23H32N6O5S2 | 536.68 | CC(C)CCn1nc(N2CCCC2)c(O)c(C2=N[S+2](O)(O)c3cc(NC(=O)C(C)S)ccc3N2)c1=O |
|  | LIGR64 | C23H32N6O6S | 520.61 | CC(C)CCn1nc(N2CCCC2)c(O)c(C2=N[S+2](O)(O)c3cc(C(O)CC(N)=O)ccc3N2)c1=O |
|  | LIGR65 | C25H36N6O5S | 532.67 | CC(C)CCn1nc(N2CCCC2)c(O)c(C2=N[S+2](O)(O)c3cc(C(=O)C(C)N(C)C)ccc3N2)c1=O |
|  | LIGR66 | C22H32N6O5S | 492.60 | CC(C)CCn1nc(N2CCCC2)c(O)c(C2=N[S+2](O)(O)c3cc(C(O)CN)ccc3N2)c1=O |
|  | LIGR67 | C21H31N7O6S2 | 541.66 | CNC(C)CCn1nc(N2CCCC2)c(O)c(C2=N[S+2](O)(O)c3cc(NS(C)(=O)=O)ccc3N2)c1=O |
|  | LIGR68 | C23H34N8O5S | 534.64 | CC(C)CCn1nc(N2CCCC2)c(O)c(C2=N[S+2](O)(O)c3cc(NC(C)C(=O)NN)ccc3N2)c1=O |
|  | LIGR69 | C21H30N6O7S2 | 542.64 | CC(CO)CCn1nc(N2CCCC2)c(O)c(C2=N[S+2](O)(O)c3cc(NS(C)(=O)=O)ccc3N2)c1=O |
|  | LIGR70 | C25H35N5O6S | 533.65 | CC(C)CCn1nc(N2CCCC2)c(O)c(C2=N[S+2](O)(O)c3cc(C(C(=O)O)C(C)C)ccc3N2)c1=O |
|  | LIGR71 | C24H33N5O7S | 535.62 | COC(=O)C(OC)c1ccc2c(c1)[S+2](O)(O)N=C(c1c(O)c(N3CCCC3)nn(CCC(C)C)c1=O)N2 |
|  | LIGR72 | C22H32N6O7S2 | 556.67 | CC(CCO)CCn1nc(N2CCCC2)c(O)c(C2=N[S+2](O)(O)c3cc(NS(C)(=O)=O)ccc3N2)c1=O |
|  | LIGR73 | C23H32N6O6S | 520.61 | COC(=O)C(N)c1ccc2c(c1)[S+2](O)(O)N=C(c1c(O)c(N3CCCC3)nn(CCC(C)C)c1=O)N2 |
|  | LIGR74 | C20H29N7O6S2 | 527.63 | CC(N)CCn1nc(N2CCCC2)c(O)c(C2=N[S+2](O)(O)c3cc(NS(C)(=O)=O)ccc3N2)c1=O |
|  | LIGR75 | C25H35N5O6S | 533.65 | CCC(OC(C)=O)c1ccc2c(c1)[S+2](O)(O)N=C(c1c(O)c(N3CCCC3)nn(CCC(C)C)c1=O)N2 |
|  | LIGR76 | C24H34N6O5S | 518.64 | CNC(C)C(=O)c1ccc2c(c1)[S+2](O)(O)N=C(c1c(O)c(N3CCCC3)nn(CCC(C)C)c1=O)N2 |
|  | LIGR77 | C25H36N6O5S | 532.67 | CCNC(C)C(=O)c1ccc2c(c1)[S+2](O)(O)N=C(c1c(O)c(N3CCCC3)nn(CCC(C)C)c1=O)N2 |
|  | LIGR78 | C23H32N6O5S | 504.61 | CC(C)CCn1nc(N2CCCC2)c(O)c(C2=N[S+2](O)(O)c3cc(C(=O)C(C)N)ccc3N2)c1=O |
|  | LIGR79 | C22H30N6O6S | 506.59 | CC(C)CCn1nc(N2CCCC2)c(O)c(C2=N[S+2](O)(O)c3cc(C(N)C(=O)O)ccc3N2)c1=O |
|  | LIGR80 | C22H31N5O6S | 493.59 | CC(C)CCn1nc(N2CCCC2)c(O)c(C2=N[S+2](O)(O)c3cc(C(O)CO)ccc3N2)c1=O |
|  | LIGR81 | C24H35N5O5S | 505.64 | CC(C)CCn1nc(N2CCCC2)c(O)c(C2=N[S+2](O)(O)c3cc(C(O)C(C)C)ccc3N2)c1=O |
|  | LIGR82 | C23H33N7O6S | 535.63 | CC(C)CCn1nc(N2CCCC2)c(O)c(C2=N[S+2](O)(O)c3cc(NCC(N)C(=O)O)ccc3N2)c1=O |
|  | LIGR83 | C23H31N5O6S | 505.60 | CC(C)CCn1nc(N2CCCC2)c(O)c(C2=N[S+2](O)(O)c3cc(OC4CCO4)ccc3N2)c1=O |
|  | LIGR83 | C23H34N6O5S | 506.63 | CNCC(O)c1ccc2c(c1)[S+2](O)(O)N=C(c1c(O)c(N3CCCC3)nn(CCC(C)C)c1=O)N2 |
|  | LIGR84 | C23H32N6O5S | 504.61 | CC(C)CCn1nc(N2CCCC2)c(O)c(C2=N[S+2](O)(O)c3cc(C(C)C(N)=O)ccc3N2)c1=O |
|  | LIGR85 | C22H29N5O7S | 507.57 | CC(C)CCn1nc(N2CCCC2)c(O)c(C2=N[S+2](O)(O)c3cc(C(O)C(=O)O)ccc3N2)c1=O |
|  | LIGR86 | C23H31N5O7S | 521.60 | COC(=O)C(O)c1ccc2c(c1)[S+2](O)(O)N=C(c1c(O)c(N3CCCC3)nn(CCC(C)C)c1=O)N2 |
|  | LIGR87 | C21H28N6O8S2 | 556.62 | CC(CCn1nc(N2CCCC2)c(O)c(C2=N[S+2](O)(O)c3cc(NS(C)(=O)=O)ccc3N2)c1=O)C(=O)O |
|  | LIGR88 | C20H28N6O7S2 | 528.61 | CC(O)CCn1nc(N2CCCC2)c(O)c(C2=N[S+2](O)(O)c3cc(NS(C)(=O)=O)ccc3N2)c1=O |
|  | LIGR90 | C24H35N5O6S | 521.64 | CCOC(C)Oc1ccc2c(c1)[S+2](O)(O)N=C(c1c(O)c(N3CCCC3)nn(CCC(C)C)c1=O)N2 |
|  | LIGR91 | C23H31N5O7S | 521.60 | CC(C)CCn1nc(N2CCCC2)c(O)c(C2=N[S+2](O)(O)c3cc(CC(O)C(=O)O)ccc3N2)c1=O |
|  | LIGR92 | C23H32N6O6S | 520.61 | CC(C)CCn1nc(N2CCCC2)c(O)c(C2=N[S+2](O)(O)c3cc(C(N)CC(=O)O)ccc3N2)c1=O |
|  | LIGR93 | C23H33N5O6S | 507.61 | CC(C)CCn1nc(N2CCCC2)c(O)c(C2=N[S+2](O)(O)c3cc(CC(O)CO)ccc3N2)c1=O |
|  | LIGR94 | C22H32N6O6S2 | 540.67 | CCC(C)CCn1nc(N2CCCC2)c(O)c(C2=N[S+2](O)(O)c3cc(NS(C)(=O)=O)ccc3N2)c1=O |
|  | LIGR95 | C25H38N6O5S | 534.68 | CC(C)CCn1nc(N2CCCC2)c(O)c(C2=N[S+2](O)(O)c3cc(C(O)CNC(C)C)ccc3N2)c1=O |
|  | LIGR96 | C24H36N6O5S | 520.66 | CCNCC(O)c1ccc2c(c1)[S+2](O)(O)N=C(c1c(O)c(N3CCCC3)nn(CCC(C)C)c1=O)N2 |
|  | LIGR97 | C24H36N6O6S | 536.66 | COCC(O)CNc1ccc2c(c1)[S+2](O)(O)N=C(c1c(O)c(N3CCCC3)nn(CCC(C)C)c1=O)N2 |
|  | LIGR98 | C23H33N5O5S | 491.61 | CCC(O)c1ccc2c(c1)[S+2](O)(O)N=C(c1c(O)c(N3CCCC3)nn(CCC(C)C)c1=O)N2 |
|  | LIGR99 | C25H36N6O5S | 532.67 | CC(C)CCn1nc(N2CCCC2)c(O)c(C2=N[S+2](O)(O)c3cc(NC4CCCC4O)ccc3N2)c1=O |
|  | LIGR  100 | C23H30N6O5S2 | 534.66 | CC(C)CCn1nc(N2CCCC2)c(O)c(C2=N[S+2](O)(O)c3cc(SC4CC(=O)N4)ccc3N2)c1=O |
