# Supplementary material for: Exploration of effective pharmacological inhibitors for NS5 protein through computational approach: A strategy to combat the neglected Kyasanur forest disease virus
Source: PLoS One. 2025 Jul 10;20(7):e0325613. doi: 10.1371/journal.pone.0325613 (PMC12244486; doi:10.1371/journal.pone.0325613)
Supplement: S5 Table — (DOCX) [file pone.0325613.s005.docx]

S5 Table. Pharmacophore characteristics of Pyridazione inhibitor

| **Characteristics**  **(Pharmacophore)** | **Co-ordinates (center)** | | | **Radius (Å)** | **Remarks** |
| --- | --- | --- | --- | --- | --- |
|  | **X** | **Y** | **Z** |  |  |
| ARO | 2.035 | 6.169 | -8.687 | 1.0 |  |
| HB-D | 4.685 | 7.101 | -8.724 | 1.0 | Considered |
| HB-D | -0.603 | 5.143 | -8.789 | 1.0 |  |
| HB-D | 7.654 | 8.146 | -6.87 | 1.0 | Considered |
| HB-D | 3.392 | 7.369 | -5.3 | 1.0 |  |
| HB-D | 1.954 | 9.166 | -6.33 | 1.0 |  |
| HB-A | 5.302 | 9.301 | -10.768 | 1.0 | Considered |
| HB-A | 7.654 | 8.146 | -6.87 | 1.0 | Considered |
| HB-A | 3.392 | 7.369 | -5.3 | 1.0 |  |
| HB-A | 1.954 | 9.166 | -6.33 | 1.0 |  |
| HB-A | -1.6 | 5.249 | -11.184 | 1.0 |  |
| HB-A | -1.727 | 7.274 | -9.733 | 1.0 | Considered |
| HP | 2.035 | 6.169 | -8.687 | 1.0 |  |
| HP | -3.294 | 5.314 | -9.189 | 1.0 |  |
| HP | 6.841 | 10.11 | -14.536 | 1.0 |  |
| HP | 10.458 | 10.315 | -6.319 | 1.0 |  |
| HP | 6.6510 | 8.838 | -14.620 | 1.0 | Considered |
| PI | 2.982 | 8.165 | -6.441 | 1.0 |  |
| PI | 8.742 | 10.338 | -8.424 | 1.0 |  |
| PI | 4.692 | 8.076 | -8.018 | 1.0 |  |

Aromatic: ARO;Hydrogen bond Acceptor:HB-A; Hydrophobic:HP; Hydrogen bond Donor:HB-D ; PI:Positive Ion
