# Supplementary material for: Exploration of effective pharmacological inhibitors for NS5 protein through computational approach: A strategy to combat the neglected Kyasanur forest disease virus
Source: PLoS One. 2025 Jul 10;20(7):e0325613. doi: 10.1371/journal.pone.0325613 (PMC12244486; doi:10.1371/journal.pone.0325613)
Supplement: S6 Table — (DOCX) [file pone.0325613.s006.docx]

S6 Table. Primary Virtual screening of 1520 compounds with binding energy value by PyRx software

| **Sr.No.** | **Ligand Name** | **Binding Energy (kcal/mol)** | **Sr.No.** | **Ligand Name** | **Binding Energy**  **(kcal/mol)** |
| --- | --- | --- | --- | --- | --- |
|  | CNP0144362.6 | -10.2 |  | CNP0368630.1 | -7.8 |
|  | CNP0202263.1 | -10.1 |  | CNP0390398.1 | -7.8 |
|  | ZINC000103114410 | -10 |  | CNP0399037 | -7.8 |
|  | CNP0212589.2 | -9.8 |  | CNP0453047 | -7.8 |
|  | CNP0133812 | -9.7 |  | CNP0473687.1 | -7.8 |
|  | CNP0208322.3 | -9.7 |  | LIG11 | -7.8 |
|  | LIGR20 | -9.7 |  | LIG21 | -7.8 |
|  | LIGR56 | -9.7 |  | LIG22 | -7.8 |
|  | CNP0205063.1 | -9.6 |  | LIG9 | -7.8 |
|  | CNP0206581 | -9.6 |  | LIGG31 | -7.8 |
|  | CNP0320547 | -9.6 |  | ZINC000000863228 | -7.8 |
|  | LIGR8 | -9.6 |  | ZINC000003823813 | -7.8 |
|  | LIGR19 | -9.5 |  | ZINC000004194000 | -7.8 |
|  | LIGR4 | -9.5 |  | ZINC000008818203 | -7.8 |
|  | ZINC000504372972 | -9.5 |  | ZINC000009329526 | -7.8 |
|  | CNP0117753 | -9.4 |  | ZINC000009459622 | -7.8 |
|  | CNP0123120.1 | -9.4 |  | ZINC000015938282 | -7.8 |
|  | CNP0124508.2 | -9.4 |  | ZINC000017016715 | -7.8 |
|  | CNP0272687.1 | -9.4 |  | ZINC000033850600 | -7.8 |
|  | CNP0331352.1 | -9.4 |  | ZINC000035572486 | -7.8 |
|  | CNP0379615.3 | -9.4 |  | ZINC000049003383 | -7.8 |
|  | LIGR12 | -9.4 |  | ZINC000057478091 | -7.8 |
|  | LIGR15 | -9.4 |  | ZINC000071748004 | -7.8 |
|  | LIGR1 | -9.4 |  | ZINC000071788407 | -7.8 |
|  | LIGR47 | -9.4 |  | ZINC000077407155 | -7.8 |
|  | LIGR50 | -9.4 |  | ZINC000219299439 | -7.8 |
|  | LIGR6 | -9.4 |  | ZINC000253429956 | -7.8 |
|  | ZINC000253523417 | -9.4 |  | ZINC000257258084 | -7.8 |
|  | ZINC000514287430 | -9.4 |  | ZINC000257331518 | -7.8 |
|  | 2325851248 | -9.3 |  | ZINC001704308488 | -7.8 |
|  | CNP0117753.2 | -9.3 |  | 110225285 | -7.7 |
|  | CNP0123015 | -9.3 |  | 135465448 | -7.7 |
|  | CNP0123120 | -9.3 |  | 135465467 | -7.7 |
|  | CNP0189030.2 | -9.3 |  | 135974582 | -7.7 |
|  | CNP0196129 | -9.3 |  | 136036674 | -7.7 |
|  | CNP0282050 | -9.3 |  | 136046535 | -7.7 |
|  | CNP0356406 | -9.3 |  | 136096851 | -7.7 |
|  | CNP0420202 | -9.3 |  | 136472999 | -7.7 |
|  | LIGG12 | -9.3 |  | 136473038 | -7.7 |
|  | LIGG5 | -9.3 |  | 136473043 | -7.7 |
|  | LIGG6 | -9.3 |  | 136487603 | -7.7 |
|  | LIGR25 | -9.3 |  | 137194574 | -7.7 |
|  | ZINC000017195339 | -9.3 |  | 143717638 | -7.7 |
|  | ZINC000040555045 | -9.3 |  | 146041809 | -7.7 |
|  | 55882280 | -9.2 |  | 155998177 | -7.7 |
|  | CNP0173802.3 | -9.2 |  | 155998202 | -7.7 |
|  | CNP0180902 | -9.2 |  | 1857527200 | -7.7 |
|  | CNP0196129.1 | -9.2 |  | 1857532584 | -7.7 |
|  | CNP0198388.1 | -9.2 |  | 54713053 | -7.7 |
|  | CNP0259215 | -9.2 |  | 54728378 | -7.7 |
|  | CNP0310261 | -9.2 |  | 54742507 | -7.7 |
|  | CNP0465153 | -9.2 |  | 68839540 | -7.7 |
|  | LIGG7 | -9.2 |  | CNP0007394 | -7.7 |
|  | LIGR100 | -9.2 |  | CNP0007974 | -7.7 |
|  | LIGR11 | -9.2 |  | CNP0016452.1 | -7.7 |
|  | LIGR2 | -9.2 |  | CNP0033932.2 | -7.7 |
|  | LIGR53 | -9.2 |  | CNP0060871 | -7.7 |
|  | LIGR7 | -9.2 |  | CNP0078310.1 | -7.7 |
|  | LIGR84 | -9.2 |  | CNP0103840 | -7.7 |
|  | ZINC000009886207 | -9.2 |  | CNP0112791 | -7.7 |
|  | 136046531 | -9.1 |  | CNP0185234 | -7.7 |
|  | 56057572 | -9.1 |  | CNP0207799 | -7.7 |
|  | CNP0005097.1 | -9.1 |  | CNP0230224 | -7.7 |
|  | CNP0188547 | -9.1 |  | CNP0265332 | -7.7 |
|  | CNP0320329.1 | -9.1 |  | CNP0290484.4 | -7.7 |
|  | CNP0359758 | -9.1 |  | CNP0296515 | -7.7 |
|  | CNP0422042 | -9.1 |  | CNP0328268.1 | -7.7 |
|  | CNP0435060.1 | -9.1 |  | CNP0401577 | -7.7 |
|  | LIGG44 | -9.1 |  | CNP0425142 | -7.7 |
|  | LIGR13 | -9.1 |  | CNP0467829 | -7.7 |
|  | LIGR16 | -9.1 |  | CNP0478012 | -7.7 |
|  | LIGR21 | -9.1 |  | LIG12 | -7.7 |
|  | LIGR22 | -9.1 |  | LIG13 | -7.7 |
|  | LIGR34 | -9.1 |  | LIG15 | -7.7 |
|  | LIGR46 | -9.1 |  | LIG20 | -7.7 |
|  | LIGR5 | -9.1 |  | LIG38 | -7.7 |
|  | LIGR65 | -9.1 |  | ZINC000004193999 | -7.7 |
|  | LIGR78 | -9.1 |  | ZINC000004834420 | -7.7 |
|  | LIGR85 | -9.1 |  | ZINC000004848436 | -7.7 |
|  | LIGR99 | -9.1 |  | ZINC000009424025 | -7.7 |
|  | ZINC000016976305 | -9.1 |  | ZINC000009470150 | -7.7 |
|  | ZINC000257282951 | -9.1 |  | ZINC000013130430 | -7.7 |
|  | 135530464 | -9 |  | ZINC000014540309 | -7.7 |
|  | 136046527 | -9 |  | ZINC000014751513 | -7.7 |
|  | 136355278 | -9 |  | ZINC000047552079 | -7.7 |
|  | 16299763 | -9 |  | ZINC000058268701 | -7.7 |
|  | CNP0097869 | -9 |  | ZINC000071614168 | -7.7 |
|  | CNP0188208.3 | -9 |  | ZINC000071751474 | -7.7 |
|  | CNP0200240 | -9 |  | ZINC000071764765 | -7.7 |
|  | CNP0253159.2 | -9 |  | ZINC000071765273 | -7.7 |
|  | CNP0318639 | -9 |  | ZINC000095397510 | -7.7 |
|  | CNP0385849 | -9 |  | ZINC000095465387 | -7.7 |
|  | CNP0387432 | -9 |  | ZINC000100564263 | -7.7 |
|  | CNP0449425 | -9 |  | ZINC000100658558 | -7.7 |
|  | LIGG11 | -9 |  | ZINC000218314899 | -7.7 |
|  | LIGG13 | -9 |  | ZINC000222943948 | -7.7 |
|  | LIGG14 | -9 |  | ZINC000253407000 | -7.7 |
|  | LIGG15 | -9 |  | ZINC000253429951 | -7.7 |
|  | LIGG20 | -9 |  | ZINC000261828172 | -7.7 |
|  | LIGR10 | -9 |  | 124053972 | -7.6 |
|  | LIGR3 | -9 |  | 135427946 | -7.6 |
|  | LIGR42 | -9 |  | 135465451 | -7.6 |
|  | LIGR48 | -9 |  | 135818242 | -7.6 |
|  | LIGR63 | -9 |  | 135955895 | -7.6 |
|  | LIGR73 | -9 |  | 136335218 | -7.6 |
|  | LIGR79 | -9 |  | 136439944 | -7.6 |
|  | LIGR86 | -9 |  | 137969937 | -7.6 |
|  | LIGR9 | -9 |  | 143247354 | -7.6 |
|  | ZINC000020610819 | -9 |  | 143662090 | -7.6 |
|  | ZINC000020611654 | -9 |  | 143696381 | -7.6 |
|  | ZINC000020611656 | -9 |  | 145520553 | -7.6 |
|  | ZINC000104296444 | -9 |  | 145892500 | -7.6 |
|  | ZINC000334160896 | -9 |  | 2049252075 | -7.6 |
|  | CNP0123015.1 | -8.9 |  | 206545382 | -7.6 |
|  | CNP0188208.2 | -8.9 |  | 86771858 | -7.6 |
|  | CNP0198388.2 | -8.9 |  | 9716232320 | -7.6 |
|  | CNP0233893 | -8.9 |  | CNP0005488.1 | -7.6 |
|  | CNP0253159 | -8.9 |  | CNP0006376.1 | -7.6 |
|  | CNP0316724.2 | -8.9 |  | CNP0071963 | -7.6 |
|  | CNP0320762 | -8.9 |  | CNP0105330.2 | -7.6 |
|  | CNP0343375.1 | -8.9 |  | CNP0119609 | -7.6 |
|  | CNP0385143 | -8.9 |  | CNP0146419 | -7.6 |
|  | CNP0385182 | -8.9 |  | CNP0148341.1 | -7.6 |
|  | LIGG1 | -8.9 |  | CNP0156788.1 | -7.6 |
|  | LIGG23 | -8.9 |  | CNP0218363.1 | -7.6 |
|  | LIGG9 | -8.9 |  | CNP0223686 | -7.6 |
|  | LIGR17 | -8.9 |  | CNP0286246.4 | -7.6 |
|  | LIGR24 | -8.9 |  | CNP0305378.1 | -7.6 |
|  | LIGR26 | -8.9 |  | CNP0311652.1 | -7.6 |
|  | LIGR45 | -8.9 |  | CNP0325649.3 | -7.6 |
|  | LIGR64 | -8.9 |  | CNP0408207 | -7.6 |
|  | LIGR81 | -8.9 |  | CNP0441687.1 | -7.6 |
|  | LIGR82 | -8.9 |  | CNP0464517.1 | -7.6 |
|  | LIGR88 | -8.9 |  | LIG10 | -7.6 |
|  | LIGR92 | -8.9 |  | LIG14 | -7.6 |
|  | LIGR95 | -8.9 |  | LIG17 | -7.6 |
|  | ZINC000003820040 | -8.9 |  | LIG18 | -7.6 |
|  | ZINC000009014304 | -8.9 |  | LIG8 | -7.6 |
|  | ZINC000009014305 | -8.9 |  | ZINC000001439654 | -7.6 |
|  | ZINC000012531078 | -8.9 |  | ZINC000004785930 | -7.6 |
|  | ZINC000016973367 | -8.9 |  | ZINC000004834432 | -7.6 |
|  | ZINC000016973372 | -8.9 |  | ZINC000005659654 | -7.6 |
|  | ZINC000016995668 | -8.9 |  | ZINC000008721154 | -7.6 |
|  | 136148564 | -8.8 |  | ZINC000008817195 | -7.6 |
|  | 137194578 | -8.8 |  | ZINC000008986584 | -7.6 |
|  | 2037880000 | -8.8 |  | ZINC000009424950 | -7.6 |
|  | 56111416 | -8.8 |  | ZINC000009437140 | -7.6 |
|  | CNP0014634.1 | -8.8 |  | ZINC000009458424 | -7.6 |
|  | CNP0097932.1 | -8.8 |  | ZINC000009460824 | -7.6 |
|  | CNP0164211 | -8.8 |  | ZINC000009766829 | -7.6 |
|  | CNP0190162 | -8.8 |  | ZINC000012527526 | -7.6 |
|  | CNP0202263 | -8.8 |  | ZINC000013955886 | -7.6 |
|  | CNP0261311.1 | -8.8 |  | ZINC000013956047 | -7.6 |
|  | CNP0265152.1 | -8.8 |  | ZINC000015671977 | -7.6 |
|  | CNP0310099 | -8.8 |  | ZINC000015672286 | -7.6 |
|  | CNP0314764.1 | -8.8 |  | ZINC000019647309 | -7.6 |
|  | CNP0314764 | -8.8 |  | ZINC000020462696 | -7.6 |
|  | CNP0362192.1 | -8.8 |  | ZINC000020612226 | -7.6 |
|  | CNP0368565 | -8.8 |  | ZINC000022269398 | -7.6 |
|  | CNP0382903.1 | -8.8 |  | ZINC000024527630 | -7.6 |
|  | LIGR23 | -8.8 |  | ZINC000058332433 | -7.6 |
|  | LIGR30 | -8.8 |  | ZINC000067245470 | -7.6 |
|  | LIGR32 | -8.8 |  | ZINC000067245493 | -7.6 |
|  | LIGR38 | -8.8 |  | ZINC000071497798 | -7.6 |
|  | LIGR51 | -8.8 |  | ZINC000072147340 | -7.6 |
|  | LIGR54 | -8.8 |  | ZINC000077262857 | -7.6 |
|  | LIGR55 | -8.8 |  | ZINC000095408300 | -7.6 |
|  | LIGR68 | -8.8 |  | ZINC000097307635 | -7.6 |
|  | LIGR70 | -8.8 |  | ZINC000098212030 | -7.6 |
|  | LIGR83 | -8.8 |  | ZINC000100564260 | -7.6 |
|  | LIGR98 | -8.8 |  | ZINC000100812224 | -7.6 |
|  | ZINC000017017142 | -8.8 |  | ZINC000102488860 | -7.6 |
|  | ZINC000257237166 | -8.8 |  | ZINC000102511762 | -7.6 |
|  | ZINC000575416790 | -8.8 |  | ZINC000103089602 | -7.6 |
|  | 1141678564 | -8.7 |  | ZINC000107147281 | -7.6 |
|  | 135955912 | -8.7 |  | ZINC000222959106 | -7.6 |
|  | 136046502 | -8.7 |  | ZINC000244884093 | -7.6 |
|  | 163555390 | -8.7 |  | ZINC000244899432 | -7.6 |
|  | CNP0005976.1 | -8.7 |  | ZINC000299763696 | -7.6 |
|  | CNP0066174 | -8.7 |  | ZINC000299802352 | -7.6 |
|  | CNP0119004 | -8.7 |  | ZINC000408959400 | -7.6 |
|  | CNP0175288.1 | -8.7 |  | ZINC000524730013 | -7.6 |
|  | CNP0200240.2 | -8.7 |  | ZINC000952844836 | -7.6 |
|  | CNP0200426 | -8.7 |  | ZINC001560410213 | -7.6 |
|  | CNP0208611.1 | -8.7 |  | 11704127 | -7.5 |
|  | CNP0244603 | -8.7 |  | 121304016 | -7.5 |
|  | CNP0257991.1 | -8.7 |  | 123920489 | -7.5 |
|  | CNP0273685.1 | -8.7 |  | 130002960 | -7.5 |
|  | CNP0390813 | -8.7 |  | 135465453 | -7.5 |
|  | CNP0423488.1 | -8.7 |  | 135818241 | -7.5 |
|  | CNP0440159 | -8.7 |  | 136169590 | -7.5 |
|  | CNP0466957 | -8.7 |  | 143662108 | -7.5 |
|  | LIGG18 | -8.7 |  | 166202659 | -7.5 |
|  | LIGG19 | -8.7 |  | 1857531310 | -7.5 |
|  | LIGG33 | -8.7 |  | 1875308838 | -7.5 |
|  | LIGG41 | -8.7 |  | 1875329998 | -7.5 |
|  | LIGG4 | -8.7 |  | 206432695 | -7.5 |
|  | LIGR18 | -8.7 |  | 54736447 | -7.5 |
|  | LIGR27 | -8.7 |  | 55969096 | -7.5 |
|  | LIGR28 | -8.7 |  | 56576613 | -7.5 |
|  | LIGR66 | -8.7 |  | 83281822 | -7.5 |
|  | LIGR74 | -8.7 |  | CNP0006351.1 | -7.5 |
|  | LIGR80 | -8.7 |  | CNP0014363 | -7.5 |
|  | LIGR91 | -8.7 |  | CNP0024870 | -7.5 |
|  | ZINC000009319819 | -8.7 |  | CNP0049777.1 | -7.5 |
|  | ZINC000013118848 | -8.7 |  | CNP0071681.2 | -7.5 |
|  | ZINC000017015470 | -8.7 |  | CNP0119415.1 | -7.5 |
|  | ZINC000020610887 | -8.7 |  | CNP0125401.9 | -7.5 |
|  | ZINC000020611465 | -8.7 |  | CNP0185234.1 | -7.5 |
|  | ZINC000022064081 | -8.7 |  | CNP0193115.1 | -7.5 |
|  | ZINC000032095213 | -8.7 |  | CNP0232150 | -7.5 |
|  | ZINC000032100163 | -8.7 |  | CNP0233077 | -7.5 |
|  | ZINC000096296013 | -8.7 |  | CNP0253329 | -7.5 |
|  | ZINC000097514680 | -8.7 |  | CNP0281938 | -7.5 |
|  | ZINC000102921292 | -8.7 |  | CNP0290510.2 | -7.5 |
|  | ZINC000253397487 | -8.7 |  | CNP0290510 | -7.5 |
|  | ZINC000253401118 | -8.7 |  | CNP0317742 | -7.5 |
|  | ZINC000575606741 | -8.7 |  | CNP0332633 | -7.5 |
|  | 136373709 | -8.6 |  | CNP0340860.2 | -7.5 |
|  | 136487614 | -8.6 |  | CNP0348129.2 | -7.5 |
|  | 136613484 | -8.6 |  | CNP0355218.2 | -7.5 |
|  | 136650834 | -8.6 |  | CNP0372993.1 | -7.5 |
|  | 137128355 | -8.6 |  | CNP0430065.1 | -7.5 |
|  | 137194584 | -8.6 |  | CNP0464624 | -7.5 |
|  | 137836573 | -8.6 |  | CNP0467817.1 | -7.5 |
|  | 16344675 | -8.6 |  | LIG36 | -7.5 |
|  | 1875402450 | -8.6 |  | LIG51 | -7.5 |
|  | 2037880067 | -8.6 |  | ZINC000001327622 | -7.5 |
|  | 55546190 | -8.6 |  | ZINC000002520871 | -7.5 |
|  | 56640146 | -8.6 |  | ZINC000003257119 | -7.5 |
|  | 90958653 | -8.6 |  | ZINC000004142281 | -7.5 |
|  | 91943913 | -8.6 |  | ZINC000004820006 | -7.5 |
|  | CNP0054971.1 | -8.6 |  | ZINC000008816528 | -7.5 |
|  | CNP0107928 | -8.6 |  | ZINC000009130709 | -7.5 |
|  | CNP0116159 | -8.6 |  | ZINC000009306173 | -7.5 |
|  | CNP0122112.2 | -8.6 |  | ZINC000009425255 | -7.5 |
|  | CNP0251948 | -8.6 |  | ZINC000009671440 | -7.5 |
|  | CNP0283955.1 | -8.6 |  | ZINC000011538620 | -7.5 |
|  | CNP0304066 | -8.6 |  | ZINC000012799734 | -7.5 |
|  | CNP0316724.1 | -8.6 |  | ZINC000014254851 | -7.5 |
|  | CNP0338460.1 | -8.6 |  | ZINC000015938278 | -7.5 |
|  | CNP0343259.1 | -8.6 |  | ZINC000017015896 | -7.5 |
|  | CNP0367053 | -8.6 |  | ZINC000019356175 | -7.5 |
|  | CNP0387025 | -8.6 |  | ZINC000019462266 | -7.5 |
|  | CNP0425604 | -8.6 |  | ZINC000020463978 | -7.5 |
|  | CNP0437380.1 | -8.6 |  | ZINC000020756916 | -7.5 |
|  | CNP0455764 | -8.6 |  | ZINC000036043165 | -7.5 |
|  | LIGG17 | -8.6 |  | ZINC000048249678 | -7.5 |
|  | LIGG22 | -8.6 |  | ZINC000059489055 | -7.5 |
|  | LIGG27 | -8.6 |  | ZINC000072410235 | -7.5 |
|  | LIGG2 | -8.6 |  | ZINC000095467508 | -7.5 |
|  | LIGG32 | -8.6 |  | ZINC000100658573 | -7.5 |
|  | LIGG37 | -8.6 |  | ZINC000102507730 | -7.5 |
|  | LIGR14 | -8.6 |  | ZINC000103110954 | -7.5 |
|  | LIGR36 | -8.6 |  | ZINC000104188697 | -7.5 |
|  | LIGR39 | -8.6 |  | ZINC000130277701 | -7.5 |
|  | LIGR41 | -8.6 |  | ZINC000170624467 | -7.5 |
|  | LIGR69 | -8.6 |  | ZINC000223421750 | -7.5 |
|  | ZINC000009317563 | -8.6 |  | ZINC000225284551 | -7.5 |
|  | ZINC000009329624 | -8.6 |  | ZINC000408948050 | -7.5 |
|  | ZINC000012406941 | -8.6 |  | ZINC000408957449 | -7.5 |
|  | ZINC000020611475 | -8.6 |  | ZINC000888090210 | -7.5 |
|  | ZINC000080325037 | -8.6 |  | ZINC000952844801 | -7.5 |
|  | ZINC000080325053 | -8.6 |  | ZINC001704332109 | -7.5 |
|  | ZINC000219466318 | -8.6 |  | 10247231 | -7.4 |
|  | ZINC000952844716 | -8.6 |  | 135444686 | -7.4 |
|  | 135424877 | -8.5 |  | 135465447 | -7.4 |
|  | 135949495 | -8.5 |  | 135818246 | -7.4 |
|  | 135955816 | -8.5 |  | 135818249 | -7.4 |
|  | 136046538 | -8.5 |  | 136344237 | -7.4 |
|  | 136148553 | -8.5 |  | 136472998 | -7.4 |
|  | 136631513 | -8.5 |  | 143662106 | -7.4 |
|  | 143696341 | -8.5 |  | 143717653 | -7.4 |
|  | 171520346 | -8.5 |  | 157013444 | -7.4 |
|  | 55880619 | -8.5 |  | 157017525 | -7.4 |
|  | 55919673 | -8.5 |  | 158664074 | -7.4 |
|  | 90956452 | -8.5 |  | 1624474069 | -7.4 |
|  | CNP0025201.1 | -8.5 |  | 246464049 | -7.4 |
|  | CNP0114503 | -8.5 |  | 247426399 | -7.4 |
|  | CNP0130962 | -8.5 |  | 45905177 | -7.4 |
|  | CNP0148000 | -8.5 |  | 55383309 | -7.4 |
|  | CNP0162743.1 | -8.5 |  | 90488169 | -7.4 |
|  | CNP0162743 | -8.5 |  | 91635777 | -7.4 |
|  | CNP0200240.1 | -8.5 |  | 92576349 | -7.4 |
|  | CNP0283955 | -8.5 |  | 97950939 | -7.4 |
|  | CNP0304693 | -8.5 |  | CNP0012488 | -7.4 |
|  | CNP0306160.1 | -8.5 |  | CNP0012826.2 | -7.4 |
|  | CNP0307268 | -8.5 |  | CNP0105330.1 | -7.4 |
|  | CNP0313199.2 | -8.5 |  | CNP0105330 | -7.4 |
|  | CNP0375032 | -8.5 |  | CNP0194425 | -7.4 |
|  | CNP0376607 | -8.5 |  | CNP0263986 | -7.4 |
|  | CNP0443976 | -8.5 |  | CNP0277344.3 | -7.4 |
|  | LIGR29 | -8.5 |  | CNP0295137 | -7.4 |
|  | LIGR31 | -8.5 |  | CNP0308028 | -7.4 |
|  | LIGR33 | -8.5 |  | CNP0340860.3 | -7.4 |
|  | LIGR37 | -8.5 |  | CNP0376779.2 | -7.4 |
|  | LIGR40 | -8.5 |  | CNP0389793.1 | -7.4 |
|  | LIGR62 | -8.5 |  | CNP0420241.1 | -7.4 |
|  | LIGR77 | -8.5 |  | CNP0420401.1 | -7.4 |
|  | LIGR93 | -8.5 |  | CNP0426041 | -7.4 |
|  | ZINC000002590388 | -8.5 |  | CNP0453284.1 | -7.4 |
|  | ZINC000004844338 | -8.5 |  | CNP0478431 | -7.4 |
|  | ZINC000009331999 | -8.5 |  | LIG19 | -7.4 |
|  | ZINC000015671969 | -8.5 |  | LIG33 | -7.4 |
|  | ZINC000020519216 | -8.5 |  | LIG37 | -7.4 |
|  | ZINC000020611029 | -8.5 |  | LIG39 | -7.4 |
|  | ZINC000020611068 | -8.5 |  | ZINC000008826377 | -7.4 |
|  | ZINC000020757636 | -8.5 |  | ZINC000008867944 | -7.4 |
|  | ZINC000035877597 | -8.5 |  | ZINC000008934420 | -7.4 |
|  | ZINC000044963335 | -8.5 |  | ZINC000009261763 | -7.4 |
|  | ZINC000049564996 | -8.5 |  | ZINC000009425062 | -7.4 |
|  | ZINC000247660882 | -8.5 |  | ZINC000009460840 | -7.4 |
|  | ZINC000604382067 | -8.5 |  | ZINC000009662095 | -7.4 |
|  | ZINC04844338 | -8.5 |  | ZINC000012557352 | -7.4 |
|  | 1298917211 | -8.4 |  | ZINC000019696611 | -7.4 |
|  | 135949507 | -8.4 |  | ZINC000019705465 | -7.4 |
|  | 135955742 | -8.4 |  | ZINC000020610716 | -7.4 |
|  | 135955768 | -8.4 |  | ZINC000025668061 | -7.4 |
|  | 135977656 | -8.4 |  | ZINC000034035806 | -7.4 |
|  | 136096764 | -8.4 |  | ZINC000038536828 | -7.4 |
|  | 136461731 | -8.4 |  | ZINC000072314266 | -7.4 |
|  | 136472268 | -8.4 |  | ZINC000079019870 | -7.4 |
|  | 136472297 | -8.4 |  | ZINC000096000195 | -7.4 |
|  | 140535835 | -8.4 |  | ZINC000100501177 | -7.4 |
|  | 154048781 | -8.4 |  | ZINC000143703253 | -7.4 |
|  | 156000422 | -8.4 |  | ZINC000219772836 | -7.4 |
|  | 1627196717 | -8.4 |  | ZINC000223410248 | -7.4 |
|  | 1875402409 | -8.4 |  | ZINC000253535202 | -7.4 |
|  | 4847401 | -8.4 |  | ZINC000408948055 | -7.4 |
|  | CNP0002431 | -8.4 |  | ZINC000524731382 | -7.4 |
|  | CNP0006254.1 | -8.4 |  | 121565264 | -7.3 |
|  | CNP0094551.1 | -8.4 |  | 135465446 | -7.3 |
|  | CNP0139025.2 | -8.4 |  | 135465452 | -7.3 |
|  | CNP0189161 | -8.4 |  | 135485533 | -7.3 |
|  | CNP0216554.2 | -8.4 |  | 135818239 | -7.3 |
|  | CNP0216922.1 | -8.4 |  | 136487636 | -7.3 |
|  | CNP0248726.1 | -8.4 |  | 155971677 | -7.3 |
|  | CNP0296367 | -8.4 |  | 1565516001 | -7.3 |
|  | CNP0305723 | -8.4 |  | 1875283151 | -7.3 |
|  | CNP0314719.1 | -8.4 |  | 55985606 | -7.3 |
|  | CNP0339890.1 | -8.4 |  | 71097345 | -7.3 |
|  | CNP0351351 | -8.4 |  | CNP0005488 | -7.3 |
|  | CNP0369737.1 | -8.4 |  | CNP0115372.2 | -7.3 |
|  | CNP0385355 | -8.4 |  | CNP0137149 | -7.3 |
|  | CNP0386293 | -8.4 |  | CNP0140017.3 | -7.3 |
|  | CNP0438250 | -8.4 |  | CNP0204218 | -7.3 |
|  | CNP0445757 | -8.4 |  | CNP0283639 | -7.3 |
|  | IGR87 | -8.4 |  | CNP0313158 | -7.3 |
|  | LIGG24 | -8.4 |  | LIG2 | -7.3 |
|  | LIGG25 | -8.4 |  | LIG3 | -7.3 |
|  | LIGG29 | -8.4 |  | LIG41 | -7.3 |
|  | LIGG36 | -8.4 |  | ZINC000001061836 | -7.3 |
|  | LIGR35 | -8.4 |  | ZINC000004158400 | -7.3 |
|  | LIGR59 | -8.4 |  | ZINC000004310999 | -7.3 |
|  | LIGR60 | -8.4 |  | ZINC000004822402 | -7.3 |
|  | LIGR61 | -8.4 |  | ZINC000006465378 | -7.3 |
|  | LIGR71 | -8.4 |  | ZINC000007425665 | -7.3 |
|  | LIGR76 | -8.4 |  | ZINC000009095912 | -7.3 |
|  | LIGR94 | -8.4 |  | ZINC000009424189 | -7.3 |
|  | LIGR96 | -8.4 |  | ZINC000009425002 | -7.3 |
|  | ZINC000004186650 | -8.4 |  | ZINC000011956761 | -7.3 |
|  | ZINC000008918560 | -8.4 |  | ZINC000024527621 | -7.3 |
|  | ZINC000012232209 | -8.4 |  | ZINC000034868036 | -7.3 |
|  | ZINC000020610580 | -8.4 |  | ZINC000035027728 | -7.3 |
|  | ZINC000020610817 | -8.4 |  | ZINC000072156466 | -7.3 |
|  | ZINC000020610828 | -8.4 |  | ZINC000072430066 | -7.3 |
|  | ZINC000020722362 | -8.4 |  | ZINC000072463820 | -7.3 |
|  | ZINC000035684315 | -8.4 |  | ZINC000096135813 | -7.3 |
|  | ZINC000051330392 | -8.4 |  | ZINC000223392668 | -7.3 |
|  | ZINC000054114293 | -8.4 |  | ZINC000408958254 | -7.3 |
|  | ZINC000077263275 | -8.4 |  | ZINC000426434519 | -7.3 |
|  | ZINC000103091956 | -8.4 |  | ZINC000426535680 | -7.3 |
|  | ZINC000103223376 | -8.4 |  | ZINC000584893538 | -7.3 |
|  | ZINC000257286206 | -8.4 |  | ZINC000585098795 | -7.3 |
|  | 135466554 | -8.3 |  | ZINC000585291905 | -7.3 |
|  | 135487650 | -8.3 |  | ZINC000626564825 | -7.3 |
|  | 135500103 | -8.3 |  | ZINC001704310815 | -7.3 |
|  | 135955691 | -8.3 |  | 135465450 | -7.2 |
|  | 136461672 | -8.3 |  | 135465469 | -7.2 |
|  | 136473037 | -8.3 |  | 135500012 | -7.2 |
|  | 136478378 | -8.3 |  | 136096872 | -7.2 |
|  | 136487596 | -8.3 |  | 136410294 | -7.2 |
|  | 136487604 | -8.3 |  | 136487615 | -7.2 |
|  | 136490097 | -8.3 |  | 136538351 | -7.2 |
|  | 136591125 | -8.3 |  | 145940140 | -7.2 |
|  | 136659420 | -8.3 |  | 155956207 | -7.2 |
|  | 160110143 | -8.3 |  | 1775959496 | -7.2 |
|  | 160110144 | -8.3 |  | 1857793844 | -7.2 |
|  | 1875325452 | -8.3 |  | 1875344766 | -7.2 |
|  | 372902582 | -8.3 |  | 56107913 | -7.2 |
|  | 372902587 | -8.3 |  | CNP0007887 | -7.2 |
|  | 54742501 | -8.3 |  | CNP0017078.1 | -7.2 |
|  | 55881643 | -8.3 |  | CNP0077552 | -7.2 |
|  | CNP0011349 | -8.3 |  | CNP0109975.1 | -7.2 |
|  | CNP0062416.1 | -8.3 |  | CNP0139665.2 | -7.2 |
|  | CNP0065764.1 | -8.3 |  | CNP0217205 | -7.2 |
|  | CNP0103443 | -8.3 |  | CNP0258248 | -7.2 |
|  | CNP0132632 | -8.3 |  | CNP0275430 | -7.2 |
|  | CNP0184194 | -8.3 |  | CNP0295966.2 | -7.2 |
|  | CNP0184794.2 | -8.3 |  | CNP0330460.1 | -7.2 |
|  | CNP0216554 | -8.3 |  | CNP0465278.1 | -7.2 |
|  | CNP0217745 | -8.3 |  | LIG1 | -7.2 |
|  | CNP0283374.4 | -8.3 |  | LIG40 | -7.2 |
|  | CNP0284506.1 | -8.3 |  | LIG43 | -7.2 |
|  | CNP0328268 | -8.3 |  | LIG44 | -7.2 |
|  | CNP0338307 | -8.3 |  | LIG47 | -7.2 |
|  | CNP0351359.2 | -8.3 |  | LIG48 | -7.2 |
|  | CNP0352021.1 | -8.3 |  | LIG4 | -7.2 |
|  | CNP0375141 | -8.3 |  | ZINC000000645776 | -7.2 |
|  | CNP0384515 | -8.3 |  | ZINC000001062437 | -7.2 |
|  | LIGG30 | -8.3 |  | ZINC000002435198 | -7.2 |
|  | LIGG39 | -8.3 |  | ZINC000006408127 | -7.2 |
|  | LIGG40 | -8.3 |  | ZINC000008780248 | -7.2 |
|  | LIGG42 | -8.3 |  | ZINC000009424751 | -7.2 |
|  | LIGG8 | -8.3 |  | ZINC000009437137 | -7.2 |
|  | LIGR75 | -8.3 |  | ZINC000012411659 | -7.2 |
|  | ZINC000002128528 | -8.3 |  | ZINC000013124221 | -7.2 |
|  | ZINC000003294746 | -8.3 |  | ZINC000057318494 | -7.2 |
|  | ZINC000008994950 | -8.3 |  | ZINC000065372024 | -7.2 |
|  | ZINC000009331433 | -8.3 |  | ZINC000069698572 | -7.2 |
|  | ZINC000014540311 | -8.3 |  | ZINC000077263966 | -7.2 |
|  | ZINC000015074508 | -8.3 |  | ZINC000096153341 | -7.2 |
|  | ZINC000019702704 | -8.3 |  | ZINC000100564262 | -7.2 |
|  | ZINC000020610811 | -8.3 |  | ZINC000104055335 | -7.2 |
|  | ZINC000020727946 | -8.3 |  | ZINC000223923858 | -7.2 |
|  | ZINC000020757510 | -8.3 |  | ZINC000253404961 | -7.2 |
|  | ZINC000020757517 | -8.3 |  | ZINC000253406275 | -7.2 |
|  | ZINC000020757641 | -8.3 |  | ZINC000299801209 | -7.2 |
|  | ZINC000035240699 | -8.3 |  | ZINC000408676889 | -7.2 |
|  | ZINC000040150332 | -8.3 |  | 1105976249 | -7.1 |
|  | ZINC000072403898 | -8.3 |  | 136404090 | -7.1 |
|  | ZINC000080325042 | -8.3 |  | 136473029 | -7.1 |
|  | ZINC000102684731 | -8.3 |  | 136487637 | -7.1 |
|  | ZINC000245255941 | -8.3 |  | 138058463 | -7.1 |
|  | ZINC000257347238 | -8.3 |  | 155989549 | -7.1 |
|  | ZINC001772491090 | -8.3 |  | 1875318575 | -7.1 |
|  | 135425236 | -8.2 |  | 1875402480 | -7.1 |
|  | 135818240 | -8.2 |  | 86771859 | -7.1 |
|  | 135869616 | -8.2 |  | 886096853 | -7.1 |
|  | 135949496 | -8.2 |  | CNP0017078 | -7.1 |
|  | 135955813 | -8.2 |  | CNP0040492.1 | -7.1 |
|  | 136036679 | -8.2 |  | CNP0040492.2 | -7.1 |
|  | 136096785 | -8.2 |  | CNP0067001 | -7.1 |
|  | 136194001 | -8.2 |  | CNP0087153.4 | -7.1 |
|  | 136347156 | -8.2 |  | CNP0265232.2 | -7.1 |
|  | 136410219 | -8.2 |  | CNP0280907.1 | -7.1 |
|  | 136461621 | -8.2 |  | CNP0292635 | -7.1 |
|  | 136461705 | -8.2 |  | CNP0328274.1 | -7.1 |
|  | 136461728 | -8.2 |  | CNP0344395.2 | -7.1 |
|  | 136473033 | -8.2 |  | CNP0359886.1 | -7.1 |
|  | 136473039 | -8.2 |  | CNP0427337.1 | -7.1 |
|  | 137194586 | -8.2 |  | CNP0477644 | -7.1 |
|  | 137194588 | -8.2 |  | LIG35 | -7.1 |
|  | 140535832 | -8.2 |  | LIG42 | -7.1 |
|  | 161664696 | -8.2 |  | LIG45 | -7.1 |
|  | 55817366 | -8.2 |  | LIG46 | -7.1 |
|  | 6453665605 | -8.2 |  | LIG50 | -7.1 |
|  | 67012032 | -8.2 |  | ZINC000001062434 | -7.1 |
|  | CNP0005162 | -8.2 |  | ZINC000004765658 | -7.1 |
|  | CNP0005220 | -8.2 |  | ZINC000009261848 | -7.1 |
|  | CNP0064280 | -8.2 |  | ZINC000009425176 | -7.1 |
|  | CNP0092600.2 | -8.2 |  | ZINC000009471394 | -7.1 |
|  | CNP0098499.2 | -8.2 |  | ZINC000009980931 | -7.1 |
|  | CNP0117009.1 | -8.2 |  | ZINC000023292489 | -7.1 |
|  | CNP0179845 | -8.2 |  | ZINC000065436092 | -7.1 |
|  | CNP0197738.2 | -8.2 |  | ZINC000100147593 | -7.1 |
|  | CNP0200426.1 | -8.2 |  | ZINC000217462425 | -7.1 |
|  | CNP0207784 | -8.2 |  | ZINC000252478116 | -7.1 |
|  | CNP0213511 | -8.2 |  | ZINC000299789768 | -7.1 |
|  | CNP0238365 | -8.2 |  | ZINC000299794426 | -7.1 |
|  | CNP0273591.1 | -8.2 |  | ZINC000299802349 | -7.1 |
|  | CNP0287263 | -8.2 |  | ZINC000426370197 | -7.1 |
|  | CNP0298240 | -8.2 |  | ZINC000426431083 | -7.1 |
|  | CNP0299541 | -8.2 |  | ZINC000585138302 | -7.1 |
|  | CNP0301822 | -8.2 |  | ZINC001704335486 | -7.1 |
|  | CNP0314182 | -8.2 |  | 136473003 | -7 |
|  | CNP0385860 | -8.2 |  | 136487605 | -7 |
|  | CNP0386629 | -8.2 |  | 246518090 | -7 |
|  | CNP0401577.1 | -8.2 |  | 28962303 | -7 |
|  | CNP0408866 | -8.2 |  | 935170814 | -7 |
|  | CNP0449050.1 | -8.2 |  | 97950940 | -7 |
|  | CNP0464849.1 | -8.2 |  | CNP0087315.6 | -7 |
|  | LIGG38 | -8.2 |  | CNP0125401.7 | -7 |
|  | LIGG3 | -8.2 |  | CNP0128651.1 | -7 |
|  | LIGR97 | -8.2 |  | CNP0291470.1 | -7 |
|  | ZINC000004819758 | -8.2 |  | CNP0297445.2 | -7 |
|  | ZINC000004820233 | -8.2 |  | CNP0393576.2 | -7 |
|  | ZINC000004820234 | -8.2 |  | LIG30 | -7 |
|  | ZINC000006735249 | -8.2 |  | LIG31 | -7 |
|  | ZINC000008994951 | -8.2 |  | LIG49 | -7 |
|  | ZINC000009405330 | -8.2 |  | ZINC000002211120 | -7 |
|  | ZINC000010219914 | -8.2 |  | ZINC000002410107 | -7 |
|  | ZINC000010219916 | -8.2 |  | ZINC000002624483 | -7 |
|  | ZINC000012406923 | -8.2 |  | ZINC000002867000 | -7 |
|  | ZINC000013041329 | -8.2 |  | ZINC000005393003 | -7 |
|  | ZINC000013427215 | -8.2 |  | ZINC000006821407 | -7 |
|  | ZINC000014743975 | -8.2 |  | ZINC000009009341 | -7 |
|  | ZINC000017107042 | -8.2 |  | ZINC000009471156 | -7 |
|  | ZINC000020610564 | -8.2 |  | ZINC000015672192 | -7 |
|  | ZINC000020610613 | -8.2 |  | ZINC000019935799 | -7 |
|  | ZINC000020611021 | -8.2 |  | ZINC000024527627 | -7 |
|  | ZINC000020611382 | -8.2 |  | ZINC000034719910 | -7 |
|  | ZINC000020756343 | -8.2 |  | ZINC000036619649 | -7 |
|  | ZINC000023114540 | -8.2 |  | ZINC000052256126 | -7 |
|  | ZINC000026977303 | -8.2 |  | ZINC000077264549 | -7 |
|  | ZINC000032096199 | -8.2 |  | ZINC000084086221 | -7 |
|  | ZINC000065553614 | -8.2 |  | ZINC000104297181 | -7 |
|  | ZINC000100502061 | -8.2 |  | ZINC000217458675 | -7 |
|  | ZINC000238950414 | -8.2 |  | ZINC000219024370 | -7 |
|  | ZINC000257224341 | -8.2 |  | ZINC000252511681 | -7 |
|  | ZINC000952844734 | -8.2 |  | ZINC000426739782 | -7 |
|  | 135424841 | -8.1 |  | ZINC000514288245 | -7 |
|  | 135485514 | -8.1 |  | ZINC000886097272 | -7 |
|  | 135813053 | -8.1 |  | 135399740 | -6.9 |
|  | 135955690 | -8.1 |  | 136487617 | -6.9 |
|  | 135955932 | -8.1 |  | 155997119 | -6.9 |
|  | 135955947 | -8.1 |  | 71959584 | -6.9 |
|  | 135974577 | -8.1 |  | CNP0003169 | -6.9 |
|  | 136117773 | -8.1 |  | CNP0036457.1 | -6.9 |
|  | 136148570 | -8.1 |  | CNP0093534 | -6.9 |
|  | 136159035 | -8.1 |  | CNP0117176 | -6.9 |
|  | 136159042 | -8.1 |  | CNP0157450.2 | -6.9 |
|  | 136167889 | -8.1 |  | CNP0238224 | -6.9 |
|  | 136194000 | -8.1 |  | CNP0247007 | -6.9 |
|  | 136473030 | -8.1 |  | CNP0281998.1 | -6.9 |
|  | 136487644 | -8.1 |  | CNP0333252.2 | -6.9 |
|  | 137194581 | -8.1 |  | CNP0353672.1 | -6.9 |
|  | 137194587 | -8.1 |  | CNP0355937 | -6.9 |
|  | 153808665 | -8.1 |  | CNP0433457.1 | -6.9 |
|  | 161664697 | -8.1 |  | LIG26 | -6.9 |
|  | 163338010 | -8.1 |  | ZINC000002413002 | -6.9 |
|  | 2037880078 | -8.1 |  | ZINC000006465367 | -6.9 |
|  | 2325853804 | -8.1 |  | ZINC000006465377 | -6.9 |
|  | 546915994 | -8.1 |  | ZINC000006533004 | -6.9 |
|  | 68622376 | -8.1 |  | ZINC000008192845 | -6.9 |
|  | 83280941 | -8.1 |  | ZINC000008826430 | -6.9 |
|  | CNP0033076 | -8.1 |  | ZINC000009262198 | -6.9 |
|  | CNP0055136 | -8.1 |  | ZINC000009469933 | -6.9 |
|  | CNP0078555 | -8.1 |  | ZINC000012554989 | -6.9 |
|  | CNP0080285 | -8.1 |  | ZINC000018600253 | -6.9 |
|  | CNP0091580.2 | -8.1 |  | ZINC000021981425 | -6.9 |
|  | CNP0113163.1 | -8.1 |  | ZINC000031576334 | -6.9 |
|  | CNP0128464 | -8.1 |  | ZINC000033689607 | -6.9 |
|  | CNP0178601.1 | -8.1 |  | ZINC000036274712 | -6.9 |
|  | CNP0197045 | -8.1 |  | ZINC000057315509 | -6.9 |
|  | CNP0203266 | -8.1 |  | ZINC000057391320 | -6.9 |
|  | CNP0217026.1 | -8.1 |  | ZINC000223526090 | -6.9 |
|  | CNP0231408 | -8.1 |  | ZINC000659013361 | -6.9 |
|  | CNP0251059.1 | -8.1 |  | ZINC000952844726 | -6.9 |
|  | CNP0273591.2 | -8.1 |  | ZINC001549161539 | -6.9 |
|  | CNP0291848.2 | -8.1 |  | 155997566 | -6.8 |
|  | CNP0301345 | -8.1 |  | 2325839857 | -6.8 |
|  | CNP0316720 | -8.1 |  | CNP0005145.1 | -6.8 |
|  | CNP0357660 | -8.1 |  | CNP0012201.1 | -6.8 |
|  | CNP0368227 | -8.1 |  | CNP0248684.3 | -6.8 |
|  | CNP0378053.2 | -8.1 |  | CNP0346349 | -6.8 |
|  | CNP0386397 | -8.1 |  | CNP0466192.1 | -6.8 |
|  | CNP0386999 | -8.1 |  | CNP0474770.1 | -6.8 |
|  | CNP0420401 | -8.1 |  | LIG25 | -6.8 |
|  | CNP0427028 | -8.1 |  | LIG32 | -6.8 |
|  | CNP0431188.2 | -8.1 |  | LIG5 | -6.8 |
|  | CNP0478391.1 | -8.1 |  | ZINC000002423645 | -6.8 |
|  | LIGG35 | -8.1 |  | ZINC000004366079 | -6.8 |
|  | LIGR49 | -8.1 |  | ZINC000004826227 | -6.8 |
|  | LIGR67 | -8.1 |  | ZINC000005180320 | -6.8 |
|  | LIGR90 | -8.1 |  | ZINC000005477684 | -6.8 |
|  | ZINC000000678568 | -8.1 |  | ZINC000006465357 | -6.8 |
|  | ZINC000001032205 | -8.1 |  | ZINC000015675194 | -6.8 |
|  | ZINC000002131088 | -8.1 |  | ZINC000026540684 | -6.8 |
|  | ZINC000002161273 | -8.1 |  | ZINC000033653012 | -6.8 |
|  | ZINC000002285841 | -8.1 |  | ZINC000035800739 | -6.8 |
|  | ZINC000004618998 | -8.1 |  | ZINC000055254201 | -6.8 |
|  | ZINC000009012719 | -8.1 |  | ZINC000058164004 | -6.8 |
|  | ZINC000009330062 | -8.1 |  | ZINC000079058006 | -6.8 |
|  | ZINC000009332000 | -8.1 |  | ZINC000217461238 | -6.8 |
|  | ZINC000009359818 | -8.1 |  | ZINC000225510473 | -6.8 |
|  | ZINC000013041331 | -8.1 |  | ZINC000247434422 | -6.8 |
|  | ZINC000020142790 | -8.1 |  | ZINC000514288247 | -6.8 |
|  | ZINC000020610541 | -8.1 |  | 136473002 | -6.7 |
|  | ZINC000020611023 | -8.1 |  | 146093648 | -6.7 |
|  | ZINC000020611463 | -8.1 |  | 1875284218 | -6.7 |
|  | ZINC000020612228 | -8.1 |  | 1875369751 | -6.7 |
|  | ZINC000021981690 | -8.1 |  | 71956933 | -6.7 |
|  | ZINC000022922211 | -8.1 |  | CNP0040662.1 | -6.7 |
|  | ZINC000032096060 | -8.1 |  | CNP0065084.1 | -6.7 |
|  | ZINC000065547669 | -8.1 |  | CNP0106649.1 | -6.7 |
|  | ZINC000067245474 | -8.1 |  | CNP0309618.1 | -6.7 |
|  | ZINC000240471655 | -8.1 |  | CNP0382448.1 | -6.7 |
|  | ZINC000408957447 | -8.1 |  | CNP0449146.1 | -6.7 |
|  | 10343986 | -8 |  | LIG6 | -6.7 |
|  | 135461048 | -8 |  | ZINC000002388588 | -6.7 |
|  | 135818248 | -8 |  | ZINC000002423648 | -6.7 |
|  | 135955717 | -8 |  | ZINC000003122277 | -6.7 |
|  | 135955795 | -8 |  | ZINC000004826228 | -6.7 |
|  | 136117762 | -8 |  | ZINC000005274106 | -6.7 |
|  | 136117772 | -8 |  | ZINC000006465296 | -6.7 |
|  | 136148569 | -8 |  | ZINC000006465325 | -6.7 |
|  | 136347367 | -8 |  | ZINC000019462262 | -6.7 |
|  | 136351972 | -8 |  | ZINC000020739908 | -6.7 |
|  | 136428317 | -8 |  | ZINC000022087602 | -6.7 |
|  | 136473040 | -8 |  | ZINC000057438537 | -6.7 |
|  | 136487642 | -8 |  | ZINC000073410556 | -6.7 |
|  | 136490093 | -8 |  | ZINC000089883934 | -6.7 |
|  | 138808766 | -8 |  | ZINC000096481405 | -6.7 |
|  | 143696361 | -8 |  | ZINC000104055328 | -6.7 |
|  | 145936684 | -8 |  | ZINC000223567352 | -6.7 |
|  | 155998185 | -8 |  | ZINC000225406796 | -6.7 |
|  | 157014556 | -8 |  | ZINC000409000492 | -6.7 |
|  | 1857676891 | -8 |  | ZINC000426715921 | -6.7 |
|  | 1875402675 | -8 |  | ZINC000585126573 | -6.7 |
|  | 2037972604 | -8 |  | 10445549 | -6.6 |
|  | 45375808 | -8 |  | 90488170 | -6.6 |
|  | 51956830 | -8 |  | CNP0088696.1 | -6.6 |
|  | 55381949 | -8 |  | CNP0163245.1 | -6.6 |
|  | 55968977 | -8 |  | CNP0348173.5 | -6.6 |
|  | 68623822 | -8 |  | CNP0379676.2 | -6.6 |
|  | 71959591 | -8 |  | CNP0379676 | -6.6 |
|  | 83279779 | -8 |  | CNP0420241.2 | -6.6 |
|  | 83279888 | -8 |  | LIG28 | -6.6 |
|  | 97536704 | -8 |  | ZINC000002412377 | -6.6 |
|  | CNP0053639.3 | -8 |  | ZINC000002413003 | -6.6 |
|  | CNP0057058.1 | -8 |  | ZINC000002434695 | -6.6 |
|  | CNP0101402 | -8 |  | ZINC000002451181 | -6.6 |
|  | CNP0131757.1 | -8 |  | ZINC000002451182 | -6.6 |
|  | CNP0209616.1 | -8 |  | ZINC000005215457 | -6.6 |
|  | CNP0216364.3 | -8 |  | ZINC000006465305 | -6.6 |
|  | CNP0270976 | -8 |  | ZINC000006465364 | -6.6 |
|  | CNP0290484 | -8 |  | ZINC000008252452 | -6.6 |
|  | CNP0338848 | -8 |  | ZINC000009306212 | -6.6 |
|  | CNP0386190 | -8 |  | ZINC000012209809 | -6.6 |
|  | CNP0389793 | -8 |  | ZINC000020350871 | -6.6 |
|  | CNP0407620 | -8 |  | ZINC000044955240 | -6.6 |
|  | CNP0449015.1 | -8 |  | ZINC000077263819 | -6.6 |
|  | CNP0449656.1 | -8 |  | ZINC000084805073 | -6.6 |
|  | CNP0468896.1 | -8 |  | ZINC000089755099 | -6.6 |
|  | LIG34 | -8 |  | ZINC000217476855 | -6.6 |
|  | LIGG43 | -8 |  | ZINC000223529857 | -6.6 |
|  | LIGR43 | -8 |  | ZINC000253403450 | -6.6 |
|  | LIGR44 | -8 |  | ZINC000253534719 | -6.6 |
|  | LIGR52 | -8 |  | ZINC000514288246 | -6.6 |
|  | LIGR57 | -8 |  | ZINC000534577754 | -6.6 |
|  | LIGR58 | -8 |  | ZINC000534662752 | -6.6 |
|  | ZINC000002130323 | -8 |  | ZINC001549159238 | -6.6 |
|  | ZINC000002130324 | -8 |  | 137992184 | -6.5 |
|  | ZINC000009014039 | -8 |  | 2325788374 | -6.5 |
|  | ZINC000009014040 | -8 |  | 37542 | -6.5 |
|  | ZINC000009034143 | -8 |  | CNP0087168 | -6.5 |
|  | ZINC000009095911 | -8 |  | CNP0157450.3 | -6.5 |
|  | ZINC000009303416 | -8 |  | CNP0189555 | -6.5 |
|  | ZINC000009354890 | -8 |  | CNP0302399 | -6.5 |
|  | ZINC000013041328 | -8 |  | CNP0428330.1 | -6.5 |
|  | ZINC000019702902 | -8 |  | LIG7 | -6.5 |
|  | ZINC000020723456 | -8 |  | ZINC000002441700 | -6.5 |
|  | ZINC000022269318 | -8 |  | ZINC000004262851 | -6.5 |
|  | ZINC000031934171 | -8 |  | ZINC000004893793 | -6.5 |
|  | ZINC000067245468 | -8 |  | ZINC000004962387 | -6.5 |
|  | ZINC000100697594 | -8 |  | ZINC000005499845 | -6.5 |
|  | ZINC000102684730 | -8 |  | ZINC000005499849 | -6.5 |
|  | ZINC000299780261 | -8 |  | ZINC000005501441 | -6.5 |
|  | 135465449 | -7.9 |  | ZINC000006465344 | -6.5 |
|  | 135465470 | -7.9 |  | ZINC000006465365 | -6.5 |
|  | 135818247 | -7.9 |  | ZINC000008826469 | -6.5 |
|  | 135818250 | -7.9 |  | ZINC000009615001 | -6.5 |
|  | 135908000 | -7.9 |  | ZINC000011845424 | -6.5 |
|  | 135949455 | -7.9 |  | ZINC000217462525 | -6.5 |
|  | 136036666 | -7.9 |  | ZINC000253388180 | -6.5 |
|  | 136148558 | -7.9 |  | ZINC000260722796 | -6.5 |
|  | 136180725 | -7.9 |  | ZINC000328603940 | -6.5 |
|  | 136347152 | -7.9 |  | ZINC001506401579 | -6.5 |
|  | 136473000 | -7.9 |  | ZINC001704313548 | -6.5 |
|  | 136473031 | -7.9 |  | CNP0106649.2 | -6.4 |
|  | 136647815 | -7.9 |  | CNP0157450.1 | -6.4 |
|  | 143662110 | -7.9 |  | LIG24 | -6.4 |
|  | 155998620 | -7.9 |  | LIG29 | -6.4 |
|  | 156001482 | -7.9 |  | ZINC000002399051 | -6.4 |
|  | 1857531228 | -7.9 |  | ZINC000002426598 | -6.4 |
|  | 54742502 | -7.9 |  | ZINC000002450320 | -6.4 |
|  | 55881891 | -7.9 |  | ZINC000005352896 | -6.4 |
|  | 57678025 | -7.9 |  | ZINC000005501445 | -6.4 |
|  | 92576348 | -7.9 |  | ZINC000005603330 | -6.4 |
|  | CNP0022753.1 | -7.9 |  | ZINC000006465297 | -6.4 |
|  | CNP0137812.1 | -7.9 |  | ZINC000006465326 | -6.4 |
|  | CNP0142948.1 | -7.9 |  | ZINC000006465345 | -6.4 |
|  | CNP0158843 | -7.9 |  | ZINC000006465382 | -6.4 |
|  | CNP0203903 | -7.9 |  | ZINC000023292487 | -6.4 |
|  | CNP0223541.1 | -7.9 |  | ZINC000033684336 | -6.4 |
|  | CNP0224269.1 | -7.9 |  | ZINC000100621169 | -6.4 |
|  | CNP0230336 | -7.9 |  | ZINC000253534720 | -6.4 |
|  | CNP0261311 | -7.9 |  | ZINC000575629767 | -6.4 |
|  | CNP0262638 | -7.9 |  | 2325878463 | -6.3 |
|  | CNP0284180 | -7.9 |  | 248467510 | -6.3 |
|  | CNP0288280.1 | -7.9 |  | CNP0124430.3 | -6.3 |
|  | CNP0324517.1 | -7.9 |  | CNP0306082.1 | -6.3 |
|  | CNP0339978.2 | -7.9 |  | CNP0348041 | -6.3 |
|  | CNP0368185.2 | -7.9 |  | CNP0348173 | -6.3 |
|  | CNP0417634.1 | -7.9 |  | LIG27 | -6.3 |
|  | CNP0452263 | -7.9 |  | ZINC000002412466 | -6.3 |
|  | LIG16 | -7.9 |  | ZINC000002418724 | -6.3 |
|  | LIG23 | -7.9 |  | ZINC000005047160 | -6.3 |
|  | LIGR72 | -7.9 |  | ZINC000006465313 | -6.3 |
|  | ZINC000004109696 | -7.9 |  | ZINC000006533027 | -6.3 |
|  | ZINC000005000191 | -7.9 |  | ZINC000013515574 | -6.3 |
|  | ZINC000007425707 | -7.9 |  | ZINC000018331576 | -6.3 |
|  | ZINC000008951475 | -7.9 |  | ZINC000018331578 | -6.3 |
|  | ZINC000009144732 | -7.9 |  | ZINC000020717076 | -6.3 |
|  | ZINC000009332239 | -7.9 |  | ZINC000020892443 | -6.3 |
|  | ZINC000012230866 | -7.9 |  | ZINC000067947987 | -6.3 |
|  | ZINC000015671865 | -7.9 |  | ZINC000089423812 | -6.3 |
|  | ZINC000016000899 | -7.9 |  | ZINC000217489816 | -6.3 |
|  | ZINC000016996545 | -7.9 |  | ZINC000585141786 | -6.3 |
|  | ZINC000017211914 | -7.9 |  | ZINC000587974054 | -6.3 |
|  | ZINC000019818591 | -7.9 |  | 166430240 | -6.2 |
|  | ZINC000020142965 | -7.9 |  | CNP0086830 | -6.2 |
|  | ZINC000022022953 | -7.9 |  | CNP0106649 | -6.2 |
|  | ZINC000031934177 | -7.9 |  | CNP0189555.3 | -6.2 |
|  | ZINC000035240698 | -7.9 |  | CNP0359454 | -6.2 |
|  | ZINC000035572490 | -7.9 |  | ZINC000012324270 | -6.2 |
|  | ZINC000100564261 | -7.9 |  | ZINC000012522941 | -6.2 |
|  | ZINC000103099558 | -7.9 |  | ZINC000022809283 | -6.2 |
|  | ZINC000107430701 | -7.9 |  | ZINC000069769931 | -6.2 |
|  | ZINC000126348215 | -7.9 |  | 57438586 | -6.1 |
|  | 10434111 | -7.8 |  | CNP0117888.1 | -6.1 |
|  | 135465468 | -7.8 |  | CNP0132980.4 | -6.1 |
|  | 135465474 | -7.8 |  | CNP0143456 | -6.1 |
|  | 135509999 | -7.8 |  | CNP0350797.2 | -6.1 |
|  | 135818251 | -7.8 |  | CNP0397173.1 | -6.1 |
|  | 136194002 | -7.8 |  | ZINC000217428387 | -6.1 |
|  | 136355279 | -7.8 |  | ZINC000217463606 | -6.1 |
|  | 136487641 | -7.8 |  | ZINC000217531028 | -6.1 |
|  | 136627737 | -7.8 |  | 164946506 | -6 |
|  | 143247368 | -7.8 |  | CNP0135294.1 | -6 |
|  | 154048783 | -7.8 |  | ZINC000004899525 | -6 |
|  | 155997131 | -7.8 |  | ZINC000095430126 | -6 |
|  | 155998186 | -7.8 |  | ZINC000217524075 | -6 |
|  | 2325786225 | -7.8 |  | ZINC000217524159 | -6 |
|  | 2325868963 | -7.8 |  | ZINC000217530942 | -6 |
|  | 257262902 | -7.8 |  | ZINC000583651893 | -6 |
|  | 531017582 | -7.8 |  | CNP0087168.1 | -5.9 |
|  | 55495956 | -7.8 |  | CNP0340544 | -5.9 |
|  | 57678028 | -7.8 |  | ZINC000217437153 | -5.9 |
|  | 6605658984 | -7.8 |  | ZINC000217461554 | -5.9 |
|  | 89271500 | -7.8 |  | 136575094 | -5.8 |
|  | 90954970 | -7.8 |  | 819331512 | -5.8 |
|  | 97536703 | -7.8 |  | CNP0106012 | -5.8 |
|  | CNP0007393 | -7.8 |  | CNP0114754.1 | -5.8 |
|  | CNP0008414 | -7.8 |  | CNP0114754 | -5.8 |
|  | CNP0012101.2 | -7.8 |  | CNP0217481.1 | -5.8 |
|  | CNP0012797 | -7.8 |  | CNP0454763.1 | -5.8 |
|  | CNP0047257.4 | -7.8 |  | ZINC000006465275 | -5.8 |
|  | CNP0047257 | -7.8 |  | ZINC000585152201 | -5.8 |
|  | CNP0127680 | -7.8 |  | CNP0223191 | -5.7 |
|  | CNP0139751 | -7.8 |  | CNP0256183 | -5.7 |
|  | CNP0178601.4 | -7.8 |  | ZINC000004026460 | -5.7 |
|  | CNP0247482.1 | -7.8 |  | ZINC000006533015 | -5.7 |
|  | CNP0263158 | -7.8 |  | CNP0141456.1 | -5.6 |
|  | CNP0265332.1 | -7.8 |  | ZINC000008076118 | -5.6 |
|  | CNP0299541.1 | -7.8 |  | 492405 | -5.5 |
|  | CNP0322044 | -7.8 |  | ZINC000217507756 | -5.5 |
|  | CNP0350453.2 | -7.8 |  | ZINC000008394184 | -5.4 |
|  | CNP0144362.6 | -10.2 |  | CNP0190580.3 | -5.3 |
