# Supplementary material for: Exploration of effective pharmacological inhibitors for NS5 protein through computational approach: A strategy to combat the neglected Kyasanur forest disease virus
Source: PLoS One. 2025 Jul 10;20(7):e0325613. doi: 10.1371/journal.pone.0325613 (PMC12244486; doi:10.1371/journal.pone.0325613)
Supplement: S7 Table — (DOCX) [file pone.0325613.s007.docx]

S7 Table. Secondary Virtual screening of 304 compounds with binding energy value by PyRx software

| **Sr.No.** | **Ligand Name** | **Binding Energy (kcal/mol)** | **Sr.No.** | **Ligand Name** | **Binding Energy**  **(kcal/mol)** |
| --- | --- | --- | --- | --- | --- |
|  | CNP0202263.1 | -10.1 |  | ZINC000096296013 | -8.9 |
|  | CNP0144362.6 | -10 |  | 136046502 | -8.8 |
|  | ZINC000103114410 | -10 |  | 136148564 | -8.8 |
|  | CNP0212589.2 | -9.8 |  | 137194578 | -8.8 |
|  | LIGR56 | -9.8 |  | 137836573 | -8.8 |
|  | CNP0133812 | -9.7 |  | 2037880000 | -8.8 |
|  | CNP0205063.1 | -9.7 |  | 2325851248 | -8.8 |
|  | CNP0272687.1 | -9.7 |  | 56057572 | -8.8 |
|  | CNP0320547 | -9.7 |  | 56111416 | -8.8 |
|  | LIGR20 | -9.7 |  | CNP0014634.1 | -8.8 |
|  | LIGR50 | -9.7 |  | CNP0066174 | -8.8 |
|  | CNP0206581 | -9.6 |  | CNP0097932.1 | -8.8 |
|  | CNP0208322.3 | -9.6 |  | CNP0173802.3 | -8.8 |
|  | CNP0331352.1 | -9.6 |  | CNP0190162 | -8.8 |
|  | LIGR19 | -9.6 |  | CNP0202263 | -8.8 |
|  | LIGR1 | -9.6 |  | CNP0244603 | -8.8 |
|  | LIGR4 | -9.6 |  | CNP0261311.1 | -8.8 |
|  | LIGR8 | -9.6 |  | CNP0265152.1 | -8.8 |
|  | ZINC000334160896 | -9.6 |  | CNP0310099 | -8.8 |
|  | CNP0117753 | -9.5 |  | CNP0314764.1 | -8.8 |
|  | CNP0196129 | -9.5 |  | CNP0367053 | -8.8 |
|  | LIGR2 | -9.5 |  | CNP0382903.1 | -8.8 |
|  | ZINC000504372972 | -9.5 |  | CNP0385182 | -8.8 |
|  | ZINC000514287430 | -9.5 |  | LIGG22 | -8.8 |
|  | CNP0123120.1 | -9.4 |  | LIGG4 | -8.8 |
|  | CNP0124508.2 | -9.4 |  | LIGR14 | -8.8 |
|  | CNP0282050 | -9.4 |  | LIGR16 | -8.8 |
|  | CNP0356406 | -9.4 |  | LIGR23 | -8.8 |
|  | CNP0379615.3 | -9.4 |  | LIGR27 | -8.8 |
|  | LIGR12 | -9.4 |  | LIGR31 | -8.8 |
|  | LIGR47 | -9.4 |  | LIGR32 | -8.8 |
|  | ZINC000253523417 | -9.4 |  | LIGR38 | -8.8 |
|  | 136046538 | -9.3 |  | LIGR45 | -8.8 |
|  | CNP0123015 | -9.3 |  | LIGR51 | -8.8 |
|  | CNP0123120 | -9.3 |  | LIGR83 | -8.8 |
|  | CNP0188547 | -9.3 |  | LIGR86 | -8.8 |
|  | CNP0368565 | -9.3 |  | LIGR98 | -8.8 |
|  | CNP0420202 | -9.3 |  | ZINC000004844338 | -8.8 |
|  | CNP0465153 | -9.3 |  | ZINC000017017142 | -8.8 |
|  | LIGG12 | -9.3 |  | ZINC000020611465 | -8.8 |
|  | LIGG5 | -9.3 |  | ZINC000020611475 | -8.8 |
|  | LIGG6 | -9.3 |  | ZINC000102921292 | -8.8 |
|  | LIGR25 | -9.3 |  | ZINC000253397487 | -8.8 |
|  | LIGR99 | -9.3 |  | ZINC000257237166 | -8.8 |
|  | ZINC000017195339 | -9.3 |  | ZINC000575416790 | -8.8 |
|  | ZINC000040555045 | -9.3 |  | 135949495 | -8.7 |
|  | ZINC000257282951 | -9.3 |  | 135955912 | -8.7 |
|  | 136355278 | -9.2 |  | 136148553 | -8.7 |
|  | 55882280 | -9.2 |  | 163555390 | -8.7 |
|  | CNP0117753.2 | -9.2 |  | CNP0005976.1 | -8.7 |
|  | CNP0180902 | -9.2 |  | CNP0119004 | -8.7 |
|  | CNP0189030.2 | -9.2 |  | CNP0123015.1 | -8.7 |
|  | CNP0196129.1 | -9.2 |  | CNP0200426 | -8.7 |
|  | CNP0198388.1 | -9.2 |  | CNP0251948 | -8.7 |
|  | CNP0310261 | -9.2 |  | CNP0257991.1 | -8.7 |
|  | CNP0422042 | -9.2 |  | CNP0273685.1 | -8.7 |
|  | LIGG14 | -9.2 |  | CNP0283955.1 | -8.7 |
|  | LIGG7 | -9.2 |  | CNP0390813 | -8.7 |
|  | LIGR100 | -9.2 |  | CNP0423488.1 | -8.7 |
|  | LIGR11 | -9.2 |  | CNP0440159 | -8.7 |
|  | LIGR15 | -9.2 |  | CNP0466957 | -8.7 |
|  | LIGR21 | -9.2 |  | LIGG17 | -8.7 |
|  | LIGR22 | -9.2 |  | LIGG27 | -8.7 |
|  | LIGR6 | -9.2 |  | LIGR28 | -8.7 |
|  | LIGR73 | -9.2 |  | LIGR36 | -8.7 |
|  | LIGR79 | -9.2 |  | LIGR39 | -8.7 |
|  | LIGR7 | -9.2 |  | LIGR91 | -8.7 |
|  | LIGR85 | -9.2 |  | LIGR93 | -8.7 |
|  | CNP0005097.1 | -9.1 |  | ZINC000009319819 | -8.7 |
|  | CNP0188208.2 | -9.1 |  | ZINC000017015470 | -8.7 |
|  | CNP0259215 | -9.1 |  | ZINC000020610887 | -8.7 |
|  | CNP0320329.1 | -9.1 |  | ZINC000022064081 | -8.7 |
|  | CNP0359758 | -9.1 |  | ZINC000032095213 | -8.7 |
|  | CNP0435060.1 | -9.1 |  | ZINC000032100163 | -8.7 |
|  | LIGG44 | -9.1 |  | ZINC000097514680 | -8.7 |
|  | LIGR13 | -9.1 |  | ZINC000253401118 | -8.7 |
|  | LIGR30 | -9.1 |  | 1141678564 | -8.6 |
|  | LIGR5 | -9.1 |  | 136046531 | -8.6 |
|  | LIGR63 | -9.1 |  | 136487614 | -8.6 |
|  | LIGR68 | -9.1 |  | 136650834 | -8.6 |
|  | LIGR69 | -9.1 |  | 137194584 | -8.6 |
|  | LIGR78 | -9.1 |  | 1875402450 | -8.6 |
|  | LIGR9 | -9.1 |  | 2037880067 | -8.6 |
|  | ZINC000009886207 | -9.1 |  | 56640146 | -8.6 |
|  | ZINC000016976305 | -9.1 |  | 91943913 | -8.6 |
|  | ZINC000020610819 | -9.1 |  | CNP0025201.1 | -8.6 |
|  | ZINC000104296444 | -9.1 |  | CNP0107928 | -8.6 |
|  | 136046527 | -9 |  | CNP0116159 | -8.6 |
|  | 16299763 | -9 |  | CNP0130962 | -8.6 |
|  | CNP0097869 | -9 |  | CNP0208611.1 | -8.6 |
|  | CNP0175288.1 | -9 |  | CNP0304066 | -8.6 |
|  | CNP0188208.3 | -9 |  | CNP0304693 | -8.6 |
|  | CNP0198388.2 | -9 |  | CNP0307268 | -8.6 |
|  | CNP0200240 | -9 |  | CNP0313199.2 | -8.6 |
|  | CNP0233893 | -9 |  | CNP0316724.1 | -8.6 |
|  | CNP0253159.2 | -9 |  | CNP0338460.1 | -8.6 |
|  | CNP0318639 | -9 |  | CNP0343259.1 | -8.6 |
|  | CNP0385849 | -9 |  | CNP0375032 | -8.6 |
|  | CNP0387432 | -9 |  | CNP0387025 | -8.6 |
|  | CNP0449425 | -9 |  | CNP0425604 | -8.6 |
|  | LIGG11 | -9 |  | CNP0437380.1 | -8.6 |
|  | LIGG13 | -9 |  | LIGG19 | -8.6 |
|  | LIGG15 | -9 |  | LIGG33 | -8.6 |
|  | LIGG18 | -9 |  | LIGG41 | -8.6 |
|  | LIGG20 | -9 |  | LIGR40 | -8.6 |
|  | LIGG23 | -9 |  | LIGR62 | -8.6 |
|  | LIGG9 | -9 |  | LIGR82 | -8.6 |
|  | LIGR10 | -9 |  | ZINC000002590388 | -8.6 |
|  | LIGR3 | -9 |  | ZINC000009317563 | -8.6 |
|  | LIGR42 | -9 |  | ZINC000009329624 | -8.6 |
|  | LIGR46 | -9 |  | ZINC000080325037 | -8.6 |
|  | LIGR48 | -9 |  | ZINC000080325053 | -8.6 |
|  | LIGR53 | -9 |  | ZINC000219466318 | -8.6 |
|  | LIGR64 | -9 |  | ZINC000575606741 | -8.6 |
|  | LIGR66 | -9 |  | ZINC000952844716 | -8.6 |
|  | LIGR77 | -9 |  | 135955816 | -8.5 |
|  | LIGR80 | -9 |  | 136373709 | -8.5 |
|  | LIGR81 | -9 |  | 136613484 | -8.5 |
|  | LIGR88 | -9 |  | 136631513 | -8.5 |
|  | LIGR92 | -9 |  | 143696341 | -8.5 |
|  | ZINC000013118848 | -9 |  | 171520346 | -8.5 |
|  | ZINC000020611654 | -9 |  | 55880619 | -8.5 |
|  | ZINC000020611656 | -9 |  | 55919673 | -8.5 |
|  | 135530464 | -8.9 |  | 90956452 | -8.5 |
|  | 16344675 | -8.9 |  | 90958653 | -8.5 |
|  | CNP0164211 | -8.9 |  | CNP0054971.1 | -8.5 |
|  | CNP0200240.2 | -8.9 |  | CNP0114503 | -8.5 |
|  | CNP0253159 | -8.9 |  | CNP0122112.2 | -8.5 |
|  | CNP0316724.2 | -8.9 |  | CNP0148000 | -8.5 |
|  | CNP0320762 | -8.9 |  | CNP0162743 | -8.5 |
|  | CNP0343375.1 | -8.9 |  | CNP0200240.1 | -8.5 |
|  | CNP0362192.1 | -8.9 |  | CNP0283955 | -8.5 |
|  | CNP0385143 | -8.9 |  | CNP0306160.1 | -8.5 |
|  | LIGG1 | -8.9 |  | CNP0314764 | -8.5 |
|  | LIGR17 | -8.9 |  | CNP0376607 | -8.5 |
|  | LIGR18 | -8.9 |  | CNP0443976 | -8.5 |
|  | LIGR24 | -8.9 |  | LIGG2 | -8.5 |
|  | LIGR26 | -8.9 |  | LIGG32 | -8.5 |
|  | LIGR34 | -8.9 |  | LIGG37 | -8.5 |
|  | LIGR54 | -8.9 |  | LIGR29 | -8.5 |
|  | LIGR55 | -8.9 |  | ZINC000009331999 | -8.5 |
|  | LIGR65 | -8.9 |  | 55546190 | -8.4 |
|  | LIGR70 | -8.9 |  | CNP0162743.1 | -8.4 |
|  | LIGR74 | -8.9 |  | LIGR33 | -8.4 |
|  | LIGR84 | -8.9 |  | LIGR37 | -8.4 |
|  | LIGR95 | -8.9 |  | LIGR41 | -8.4 |
|  | ZINC000003820040 | -8.9 |  | ZINC000012406941 | -8.4 |
|  | ZINC000009014304 | -8.9 |  | ZINC000012531078 | -8.4 |
|  | ZINC000009014305 | -8.9 |  | ZINC000015671969 | -8.4 |
|  | ZINC000016973367 | -8.9 |  | 137128355 | -8.3 |
|  | ZINC000016973372 | -8.9 |  | CNP0455764 | -8.1 |
|  | ZINC000016995668 | -8.9 |  | 135424877 | -7.9 |
