# Supplementary material for: Exploration of effective pharmacological inhibitors for NS5 protein through computational approach: A strategy to combat the neglected Kyasanur forest disease virus
Source: PLoS One. 2025 Jul 10;20(7):e0325613. doi: 10.1371/journal.pone.0325613 (PMC12244486; doi:10.1371/journal.pone.0325613)
Supplement: S8 Table — (DOCX) [file pone.0325613.s008.docx]

**S8 Table. Swiss ADME: Selected drugs ADME characterization**

| **Molecule** | **CNP0331352.1(L2)** | **ZINC000103114410)L3)** | **CNP0202263.1(L4)** | **ZINC000514287430(L5)** | **136046538(L6)** | **CNP0272687.1(L7)** | **Dasabuvir(L1)** |
| --- | --- | --- | --- | --- | --- | --- | --- |
| Formula | C_21_H_18_O_12_ | C_23_H_17_N_3_O_5_ | C_26_H_26_O_9_ | C_26_H_32_N_4_O_5_ | C_20_H_21_N_5_O_4_S_2_ | C_24_H_20_O_10_ | C_26_H_27_N_3_O_5_S |
| **Physico-chemical properties** | | | | | | |  |
| Weight  (molecular)(Da) | 462.36 | 415.4 | 482.48 | 480.56 | 459.54 | 468.41 | 493.57 |
| #Heavy atoms | 33 | 31 | 35 | 35 | 31 | 34 | 35 |
| #Rotatable bonds | 3 | 4 | 3 | 2 | 5 | 3 | 6 |
| #Aromatic heavy atoms | 16 | 16 | 16 | 12 | 17 | 20 | 22 |
| #H-bond donors | 5 | 2 | 5 | 3 | 3 | 4 | 2 |
| #H-bond acceptors | 12 | 6 | 9 | 7 | 6 | 10 | 5 |
| MR | 110.74 | 120.45 | 124.46 | 139.44 | 128.64 | 118.97 | 139.63 |
| TPSA | 189.26 | 100.46 | 145.91 | 113.02 | 176.29 | 159.8 | 118.64 |
| Lipophilicity  Consensus Log P | 0.38 | 3.06 | 1.48 | 0.98 | 2.69 | 1.53 | 3.80 |
| Water Solubility  ESOL Log S | -2.95 | -5.23 | -4.46 | -3.7 | -4.43 | -3.93 | -5.65 |
| Water Solubility  ESOL Class | Soluble | Moderately soluble | Moderately soluble | Soluble | Moderately soluble | Soluble | Moderately soluble |
| **Pharmacokinetics** | | | | | | |  |
| GI absorption | Low | High | Low | High | Low | Low | Low |
| log Kp (cm/s) skin permeation | -9.03 | -5.8 | -7.56 | -8.38 | -7.23 | -8.09 | -6.29 |
| BBB permeate | No | No | No | No | No | No | No |
| **Drug likeness** | | | | | | |  |
| Lipinski #violations | 1 | 0 | 0 | 0 | 0 | 0 | 0 |
| Veber #violations | 1 | 0 | 1 | 0 | 1 | 1 | 0 |
| Bioavailability Score | 0.55 | 0.55 | 0.55 | 0.55 | 0.55 | 0.55 | 0.56 |
| PAINS #alerts | 1 | 1 | 0 | 0 | 0 | 0 | 0 |
| Leadlikeness #violations | 1 | 2 | 1 | 1 | 1 | 1 | 2 |
| Synthetic Accessibility | 5.29 | 3.7 | 5.36 | 6.34 | 4.18 | 5.37 | 3.46 |
| **Metabolism** | | | | | | |  |
| Pgp substrate | N | N | N | Y | N | N | N |
| CYP2D6 inhibitor | N | N | N | Y | N | N | N |
| CYP2C9 inhibitor | N | Y | N | N | N | N | Y |
| CYP2C19 inhibitor | N | Y | N | N | N | N | Y |
| CYP1A2 inhibitor | N | Y | N | N | N | N | Y |
| CYP3A4 inhibitor | N | N | N | N | N | N | N |
