# Supplementary figures and images for: Exploration of effective pharmacological inhibitors for NS5 protein through computational approach: A strategy to combat the neglected Kyasanur forest disease virus
Source: PLoS One. 2025 Jul 10;20(7):e0325613. doi: 10.1371/journal.pone.0325613 (PMC12244486; doi:10.1371/journal.pone.0325613)

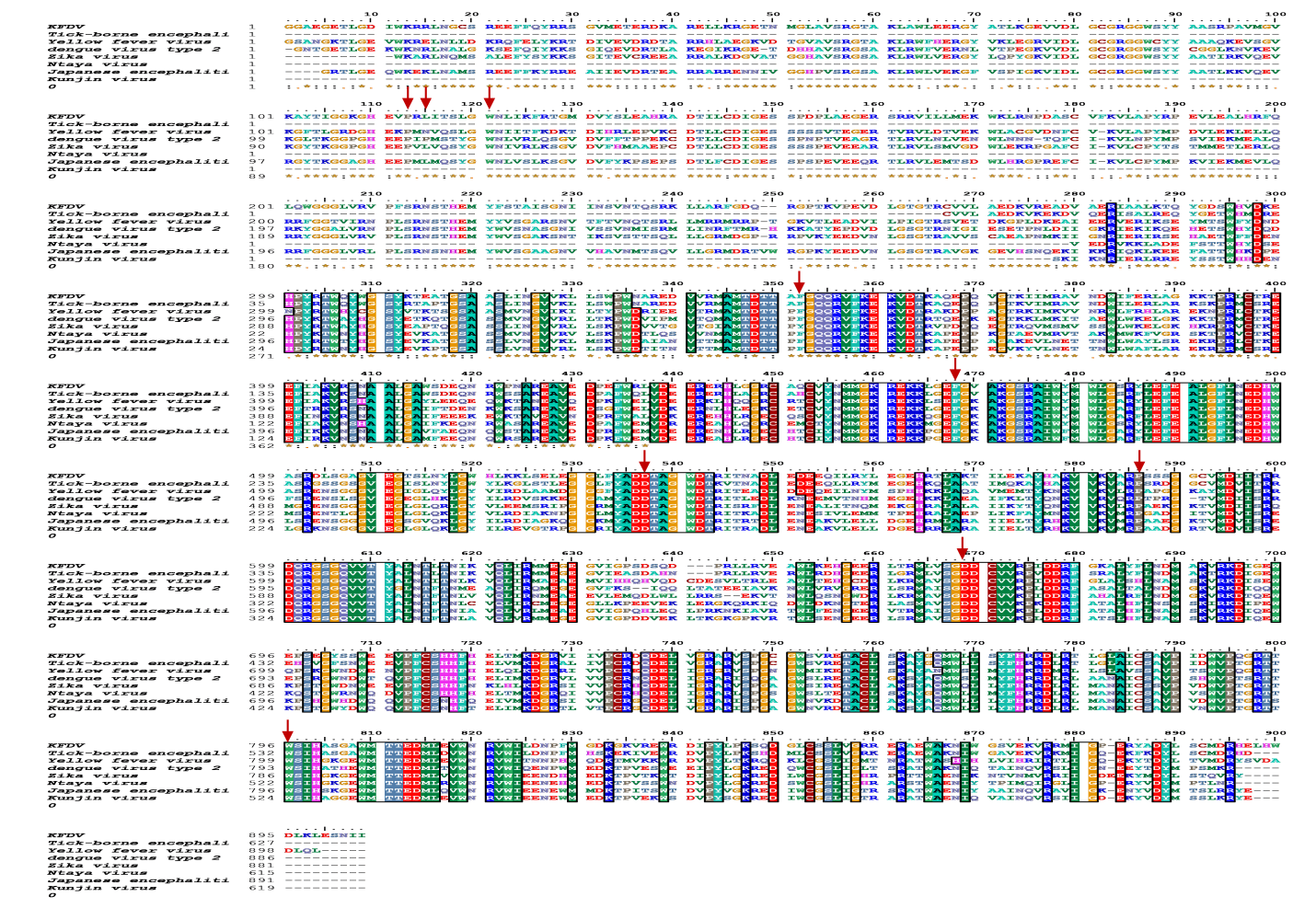


S1 Fig. MSA between NS5 protein of KFDV and homologous sequence

Supplement: S1 Fig — (DOCX) [file pone.0325613.s009.docx]

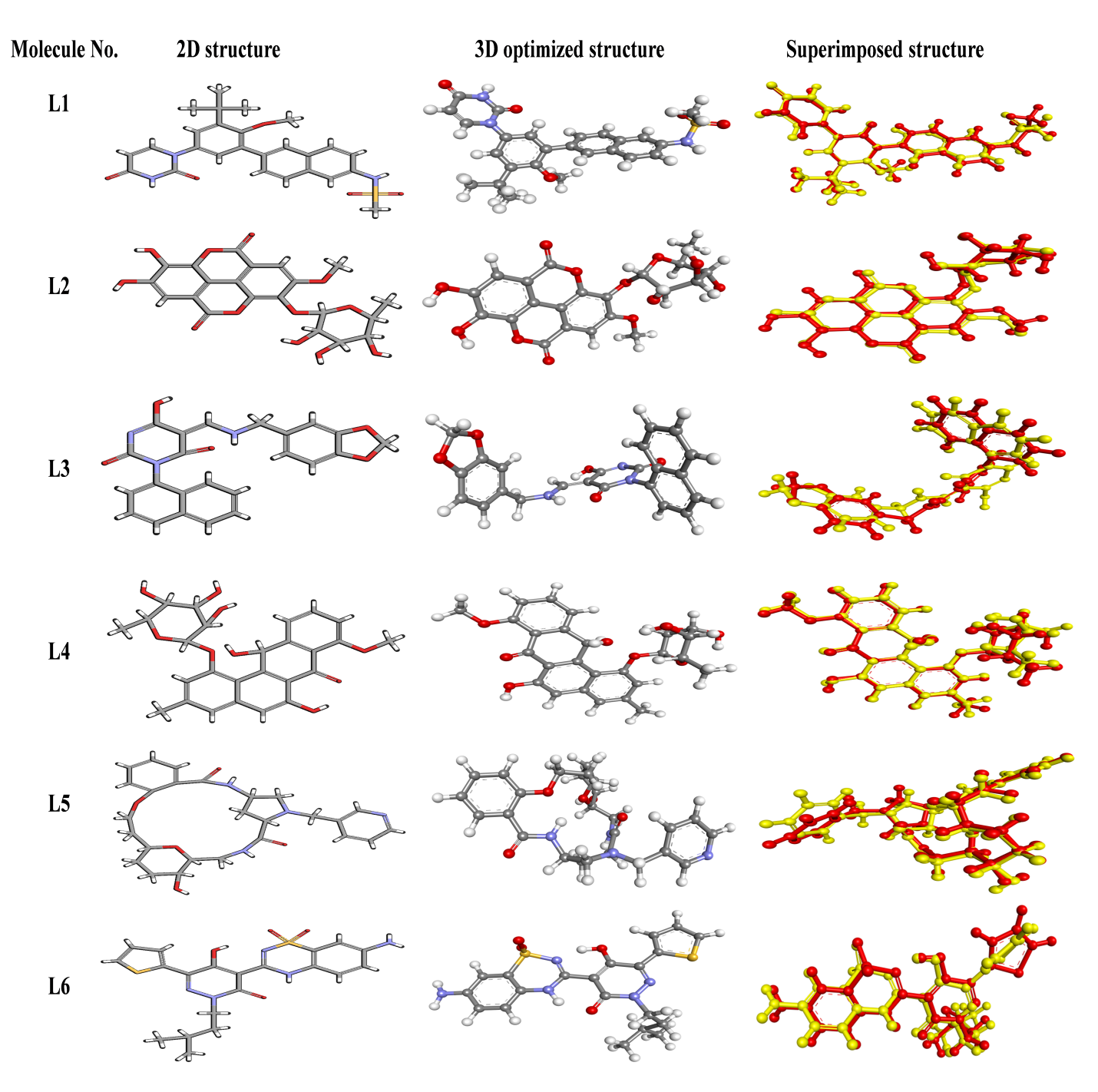


**S2 Fig. Geometry optimization of ligands by Orca software**

Supplement: S2 Fig — (DOCX) [file pone.0325613.s010.docx]

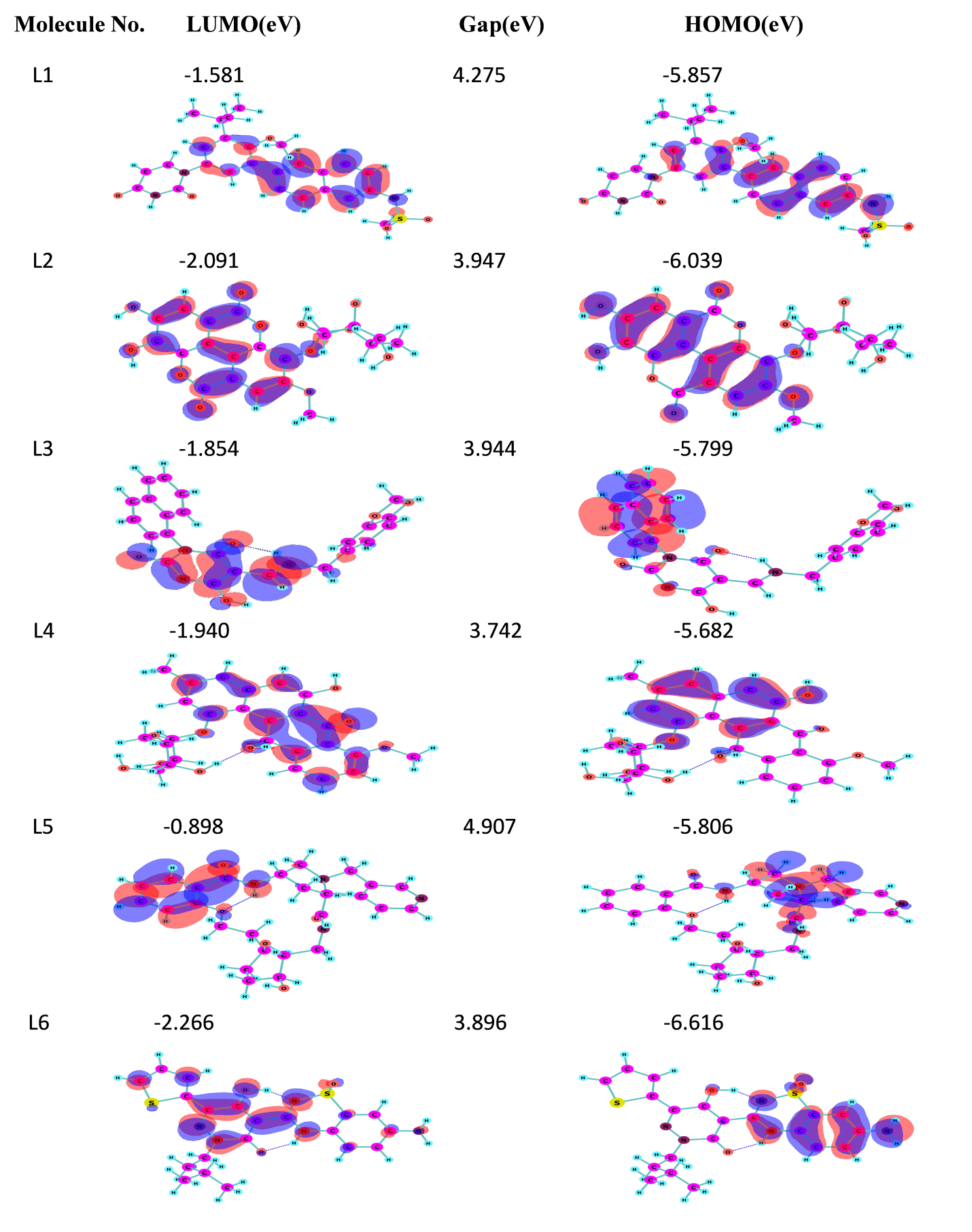


**S3 Fig.FMO reflecting HOMO-LUMO and energy gap of the selected ligands**

Supplement: S3 Fig — (DOCX) [file pone.0325613.s011.docx]

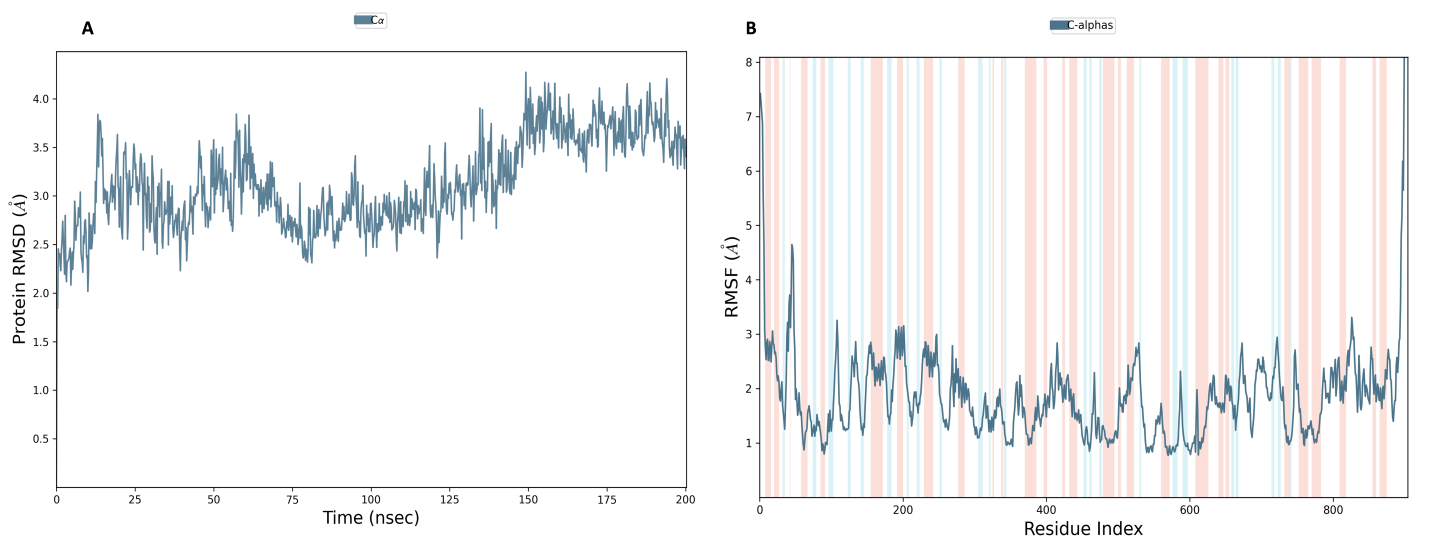


**S4 Fig. A. RMSD and B. RMSF analysis of NS5 protein at 200 ns of replica2**

Supplement: S4 Fig — (DOCX) [file pone.0325613.s012.docx]

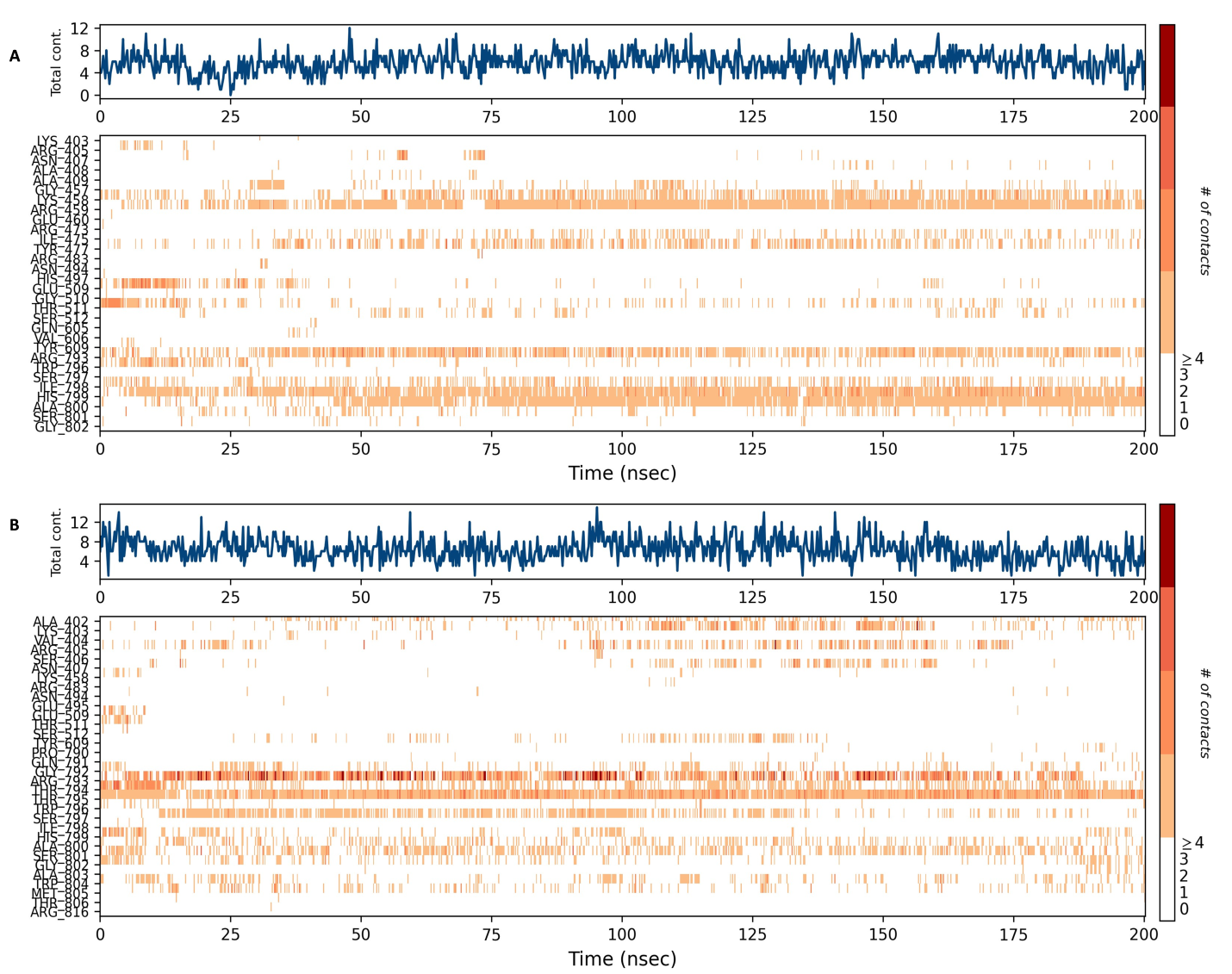


**S11 Fig. NS5 protein-ligand interaction timeline of replica 1(A) NS5-L1 complex, (B) NS5-L2 complex.**

Supplement: S11 Fig — (DOCX) [file pone.0325613.s019.docx]

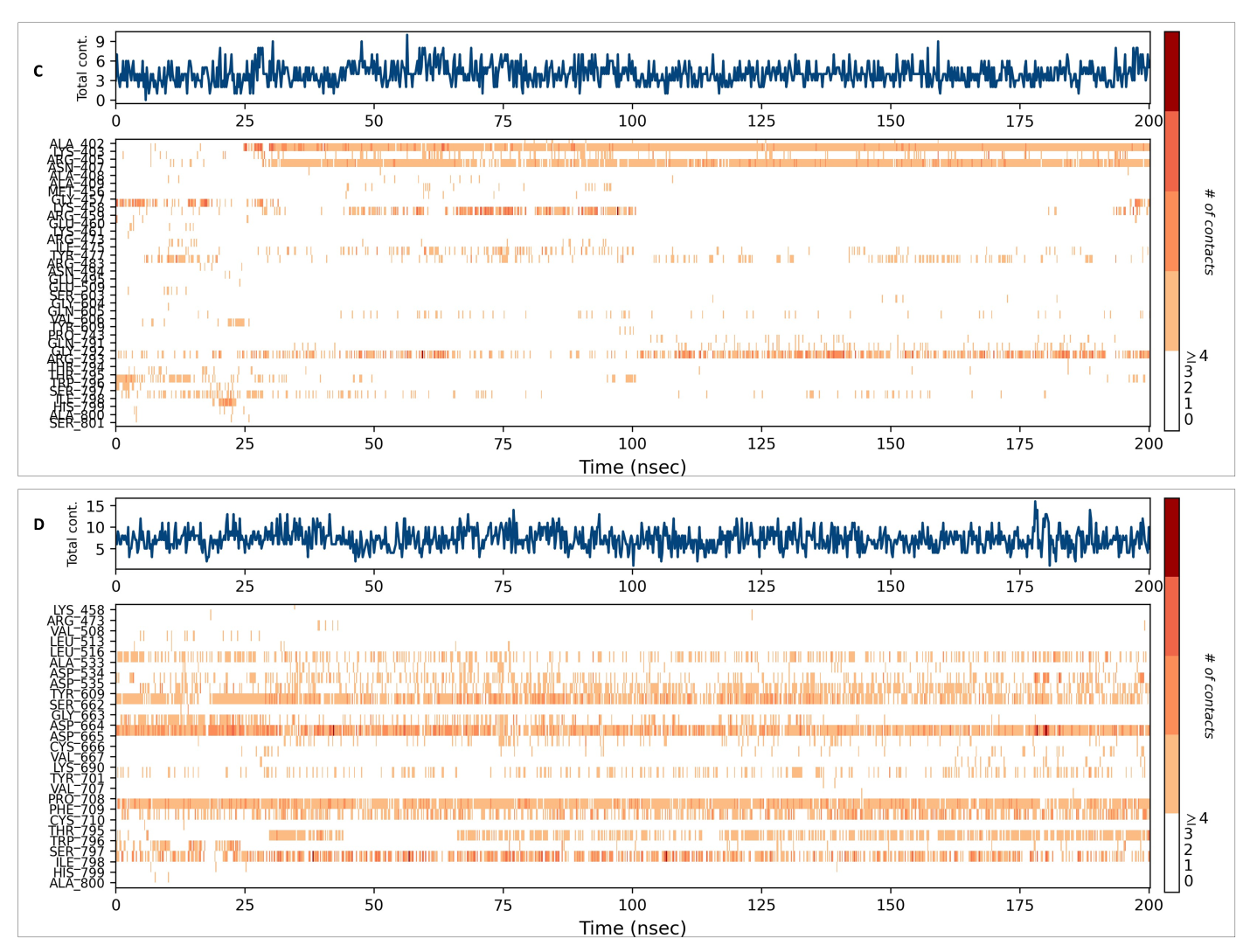


**S12 Fig. NS5 protein-ligand interaction timeline of replica 1 (C) NS5-L3 complex, & (D) NS5-L4 complex**

Supplement: S12 Fig — (DOCX) [file pone.0325613.s020.docx]

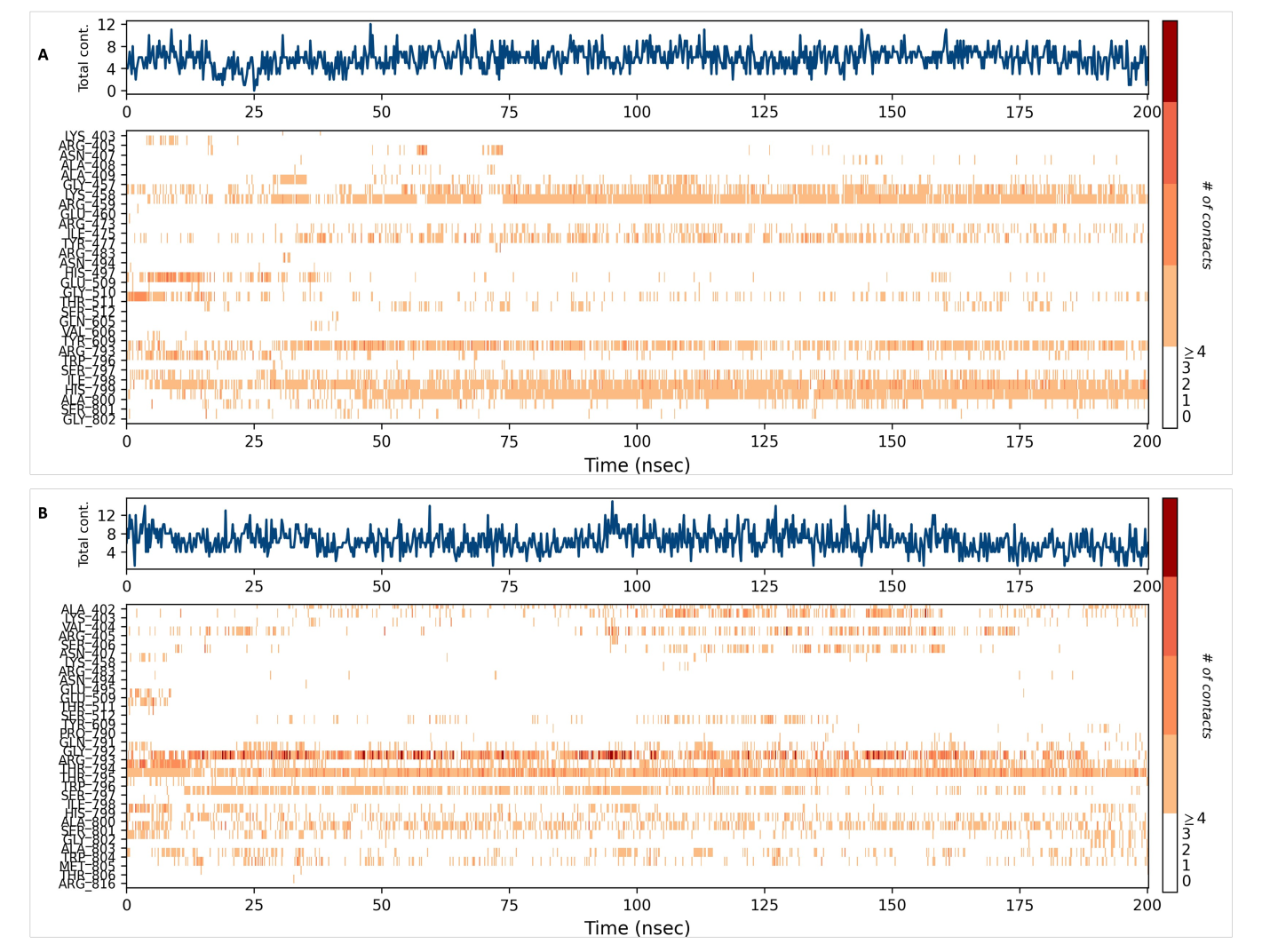


**S13 Fig. NS5 protein-ligand interaction timeline of replica 2(A) NS5-L1 complex, (B) NS5-L2 complex.**

Supplement: S13 Fig — (DOCX) [file pone.0325613.s021.docx]

**
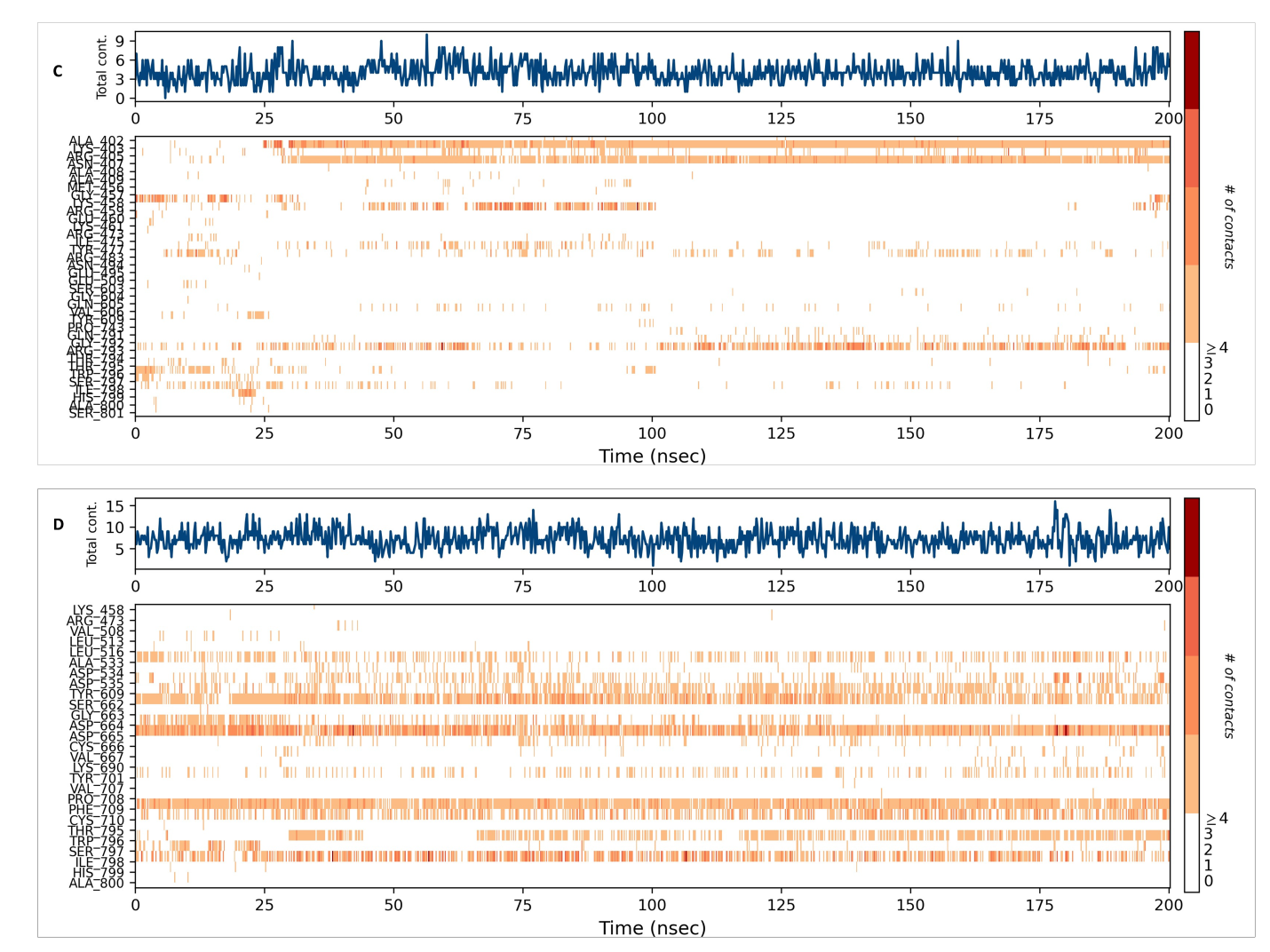
**

**S14 Fig. NS5 protein-ligand interaction timeline of replica 2 (C) NS5-L3 complex, & (D) NS5-L4 complex**

Supplement: S14 Fig — (DOCX) [file pone.0325613.s022.docx]
