# Supplementary material for: Exploration of effective pharmacological inhibitors for NS5 protein through computational approach: A strategy to combat the neglected Kyasanur forest disease virus
Source: PLoS One. 2025 Jul 10;20(7):e0325613. doi: 10.1371/journal.pone.0325613 (PMC12244486; doi:10.1371/journal.pone.0325613)
Supplement: S5 Fig — (DOCX) [file pone.0325613.s013.docx]

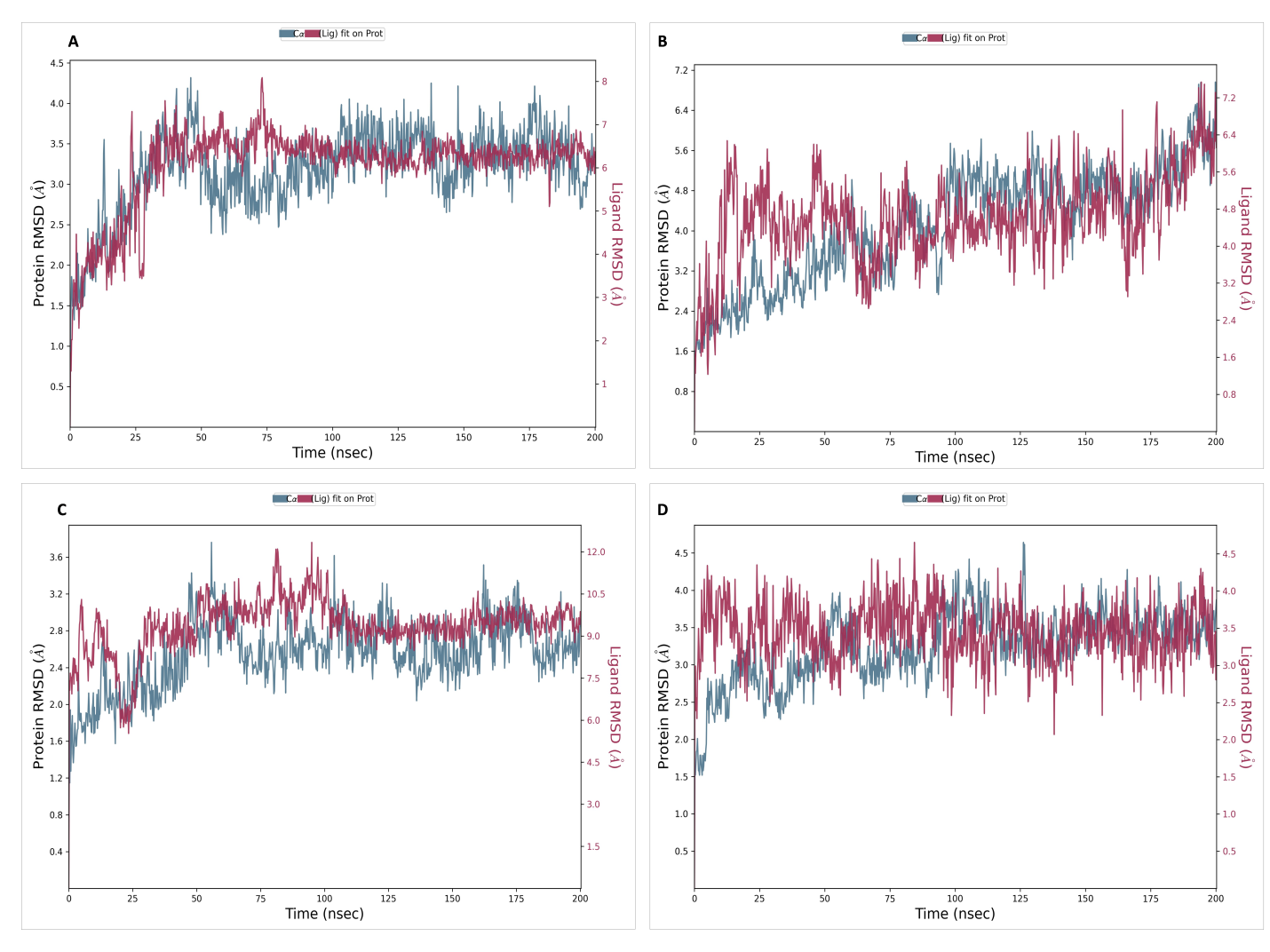


**S5 Fig. RMSD trajectory of NS5-ligand complex at 200 ns of replica 2(A) NS5-L3 complex, (B) NS5-L5 complex, (C) NS5-L6 complex, & (D) NS5-dasabuvir complex**
