# Supplementary material for: Exploration of effective pharmacological inhibitors for NS5 protein through computational approach: A strategy to combat the neglected Kyasanur forest disease virus
Source: PLoS One. 2025 Jul 10;20(7):e0325613. doi: 10.1371/journal.pone.0325613 (PMC12244486; doi:10.1371/journal.pone.0325613)
Supplement: S6 Fig — (DOCX) [file pone.0325613.s014.docx]

**
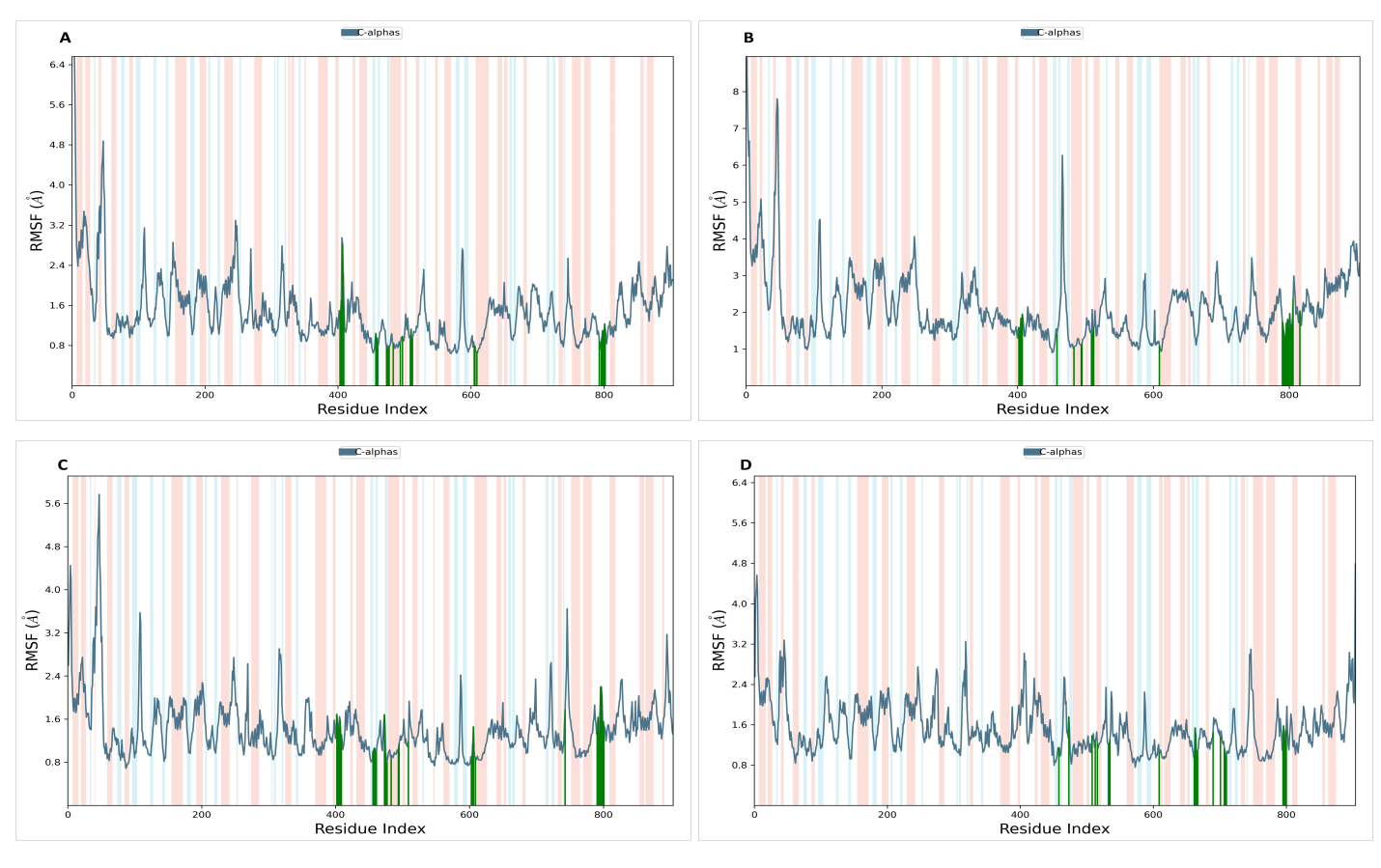
**

**S6 Fig. RMSF trajectory of NS5-ligand complex at 200 ns of replica2(A) NS5-L1 complex, (B) NS5-L2 complex, (C) NS5-L3 complex, & (D) NS5-L4 complex**
