# Supplementary material for: Exploration of effective pharmacological inhibitors for NS5 protein through computational approach: A strategy to combat the neglected Kyasanur forest disease virus
Source: PLoS One. 2025 Jul 10;20(7):e0325613. doi: 10.1371/journal.pone.0325613 (PMC12244486; doi:10.1371/journal.pone.0325613)
Supplement: S7 Fig — (DOCX) [file pone.0325613.s015.docx]

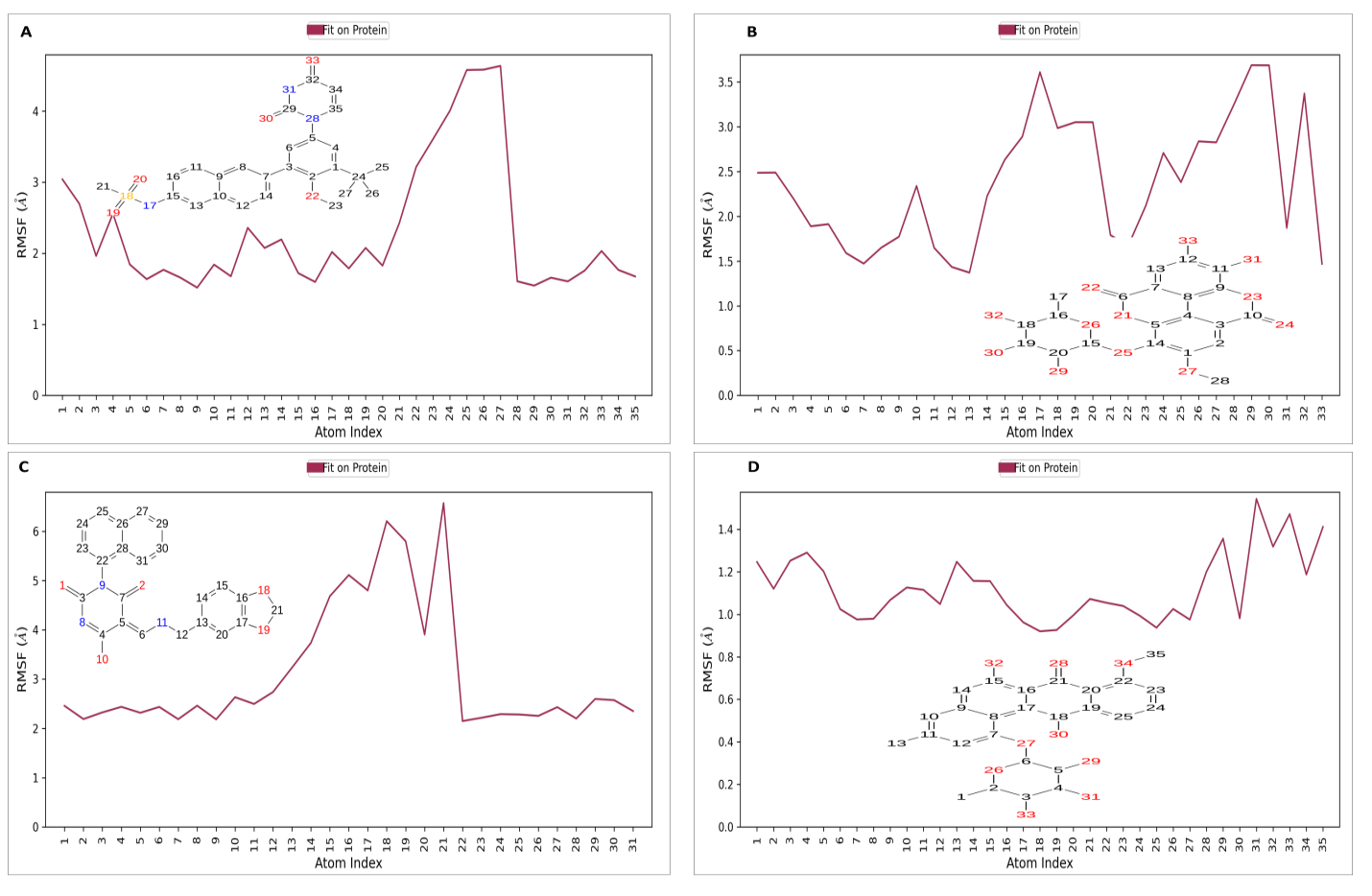


**S7 Fig. Ligand RMSF plot of NS5-ligand complex at 200 ns of replica 1(A) L1 ligand, (B) L2 ligand, (C) L3 ligand, & (D) L4 ligand**
