# Supplementary material for: Exploration of effective pharmacological inhibitors for NS5 protein through computational approach: A strategy to combat the neglected Kyasanur forest disease virus
Source: PLoS One. 2025 Jul 10;20(7):e0325613. doi: 10.1371/journal.pone.0325613 (PMC12244486; doi:10.1371/journal.pone.0325613)
Supplement: S9 Fig — (DOCX) [file pone.0325613.s017.docx]

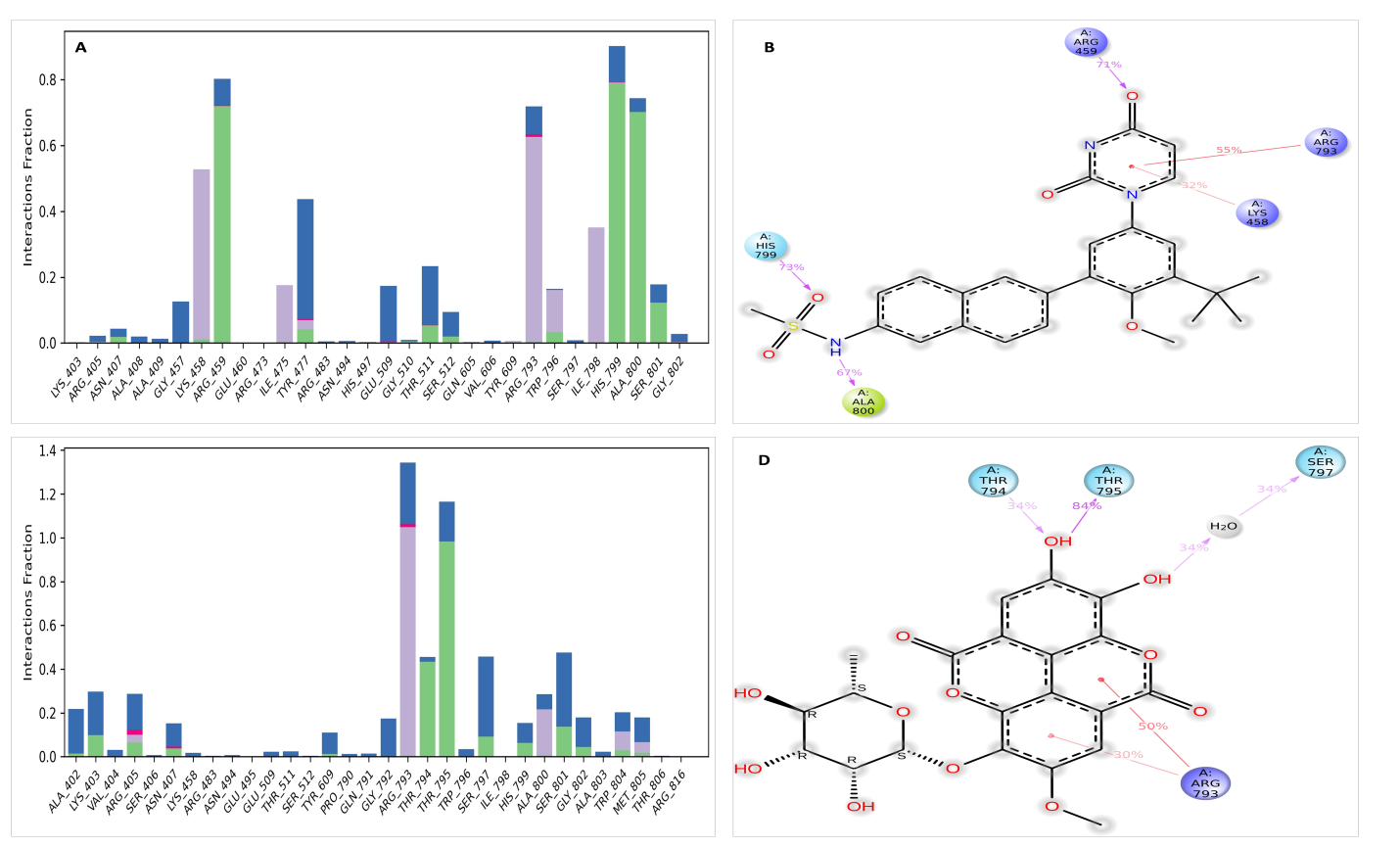
**S9 Fig. NS5-ligand interaction map of replica 2(A) NS5-L1 complex, (C) NS5-L2 complex and NS5-ligand contact for more than 30% simulation time (B) NS5-L1 complex, (D) NS5-L2 complex.**
