# Supplementary material for: Exploration of effective pharmacological inhibitors for NS5 protein through computational approach: A strategy to combat the neglected Kyasanur forest disease virus
Source: PLoS One. 2025 Jul 10;20(7):e0325613. doi: 10.1371/journal.pone.0325613 (PMC12244486; doi:10.1371/journal.pone.0325613)
Supplement: S10 Fig — (DOCX) [file pone.0325613.s018.docx]

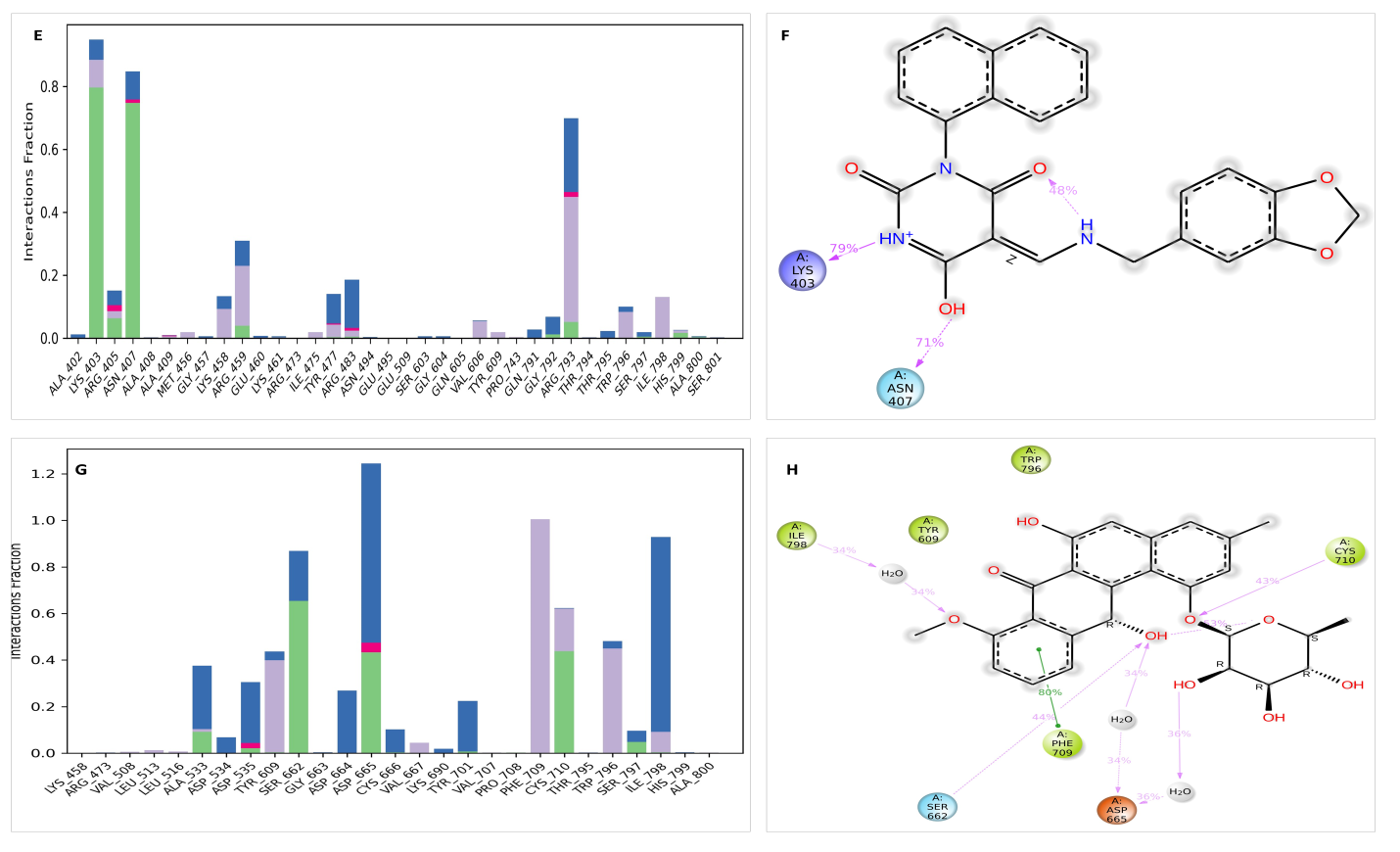


**S10 Fig. NS5-ligand interaction map of replica2 (E) NS5-L3 complex, & (G) NS5-L4 complex and NS5-ligand contact for more than 30% simulation time (F) NS5-L3 complex, & (H) NS5-L4 complex**
