# Supplementary material for: Exploration of effective pharmacological inhibitors for NS5 protein through computational approach: A strategy to combat the neglected Kyasanur forest disease virus
Source: PLoS One. 2025 Jul 10;20(7):e0325613. doi: 10.1371/journal.pone.0325613 (PMC12244486; doi:10.1371/journal.pone.0325613)
Supplement: S15 Fig — (DOCX) [file pone.0325613.s023.docx]

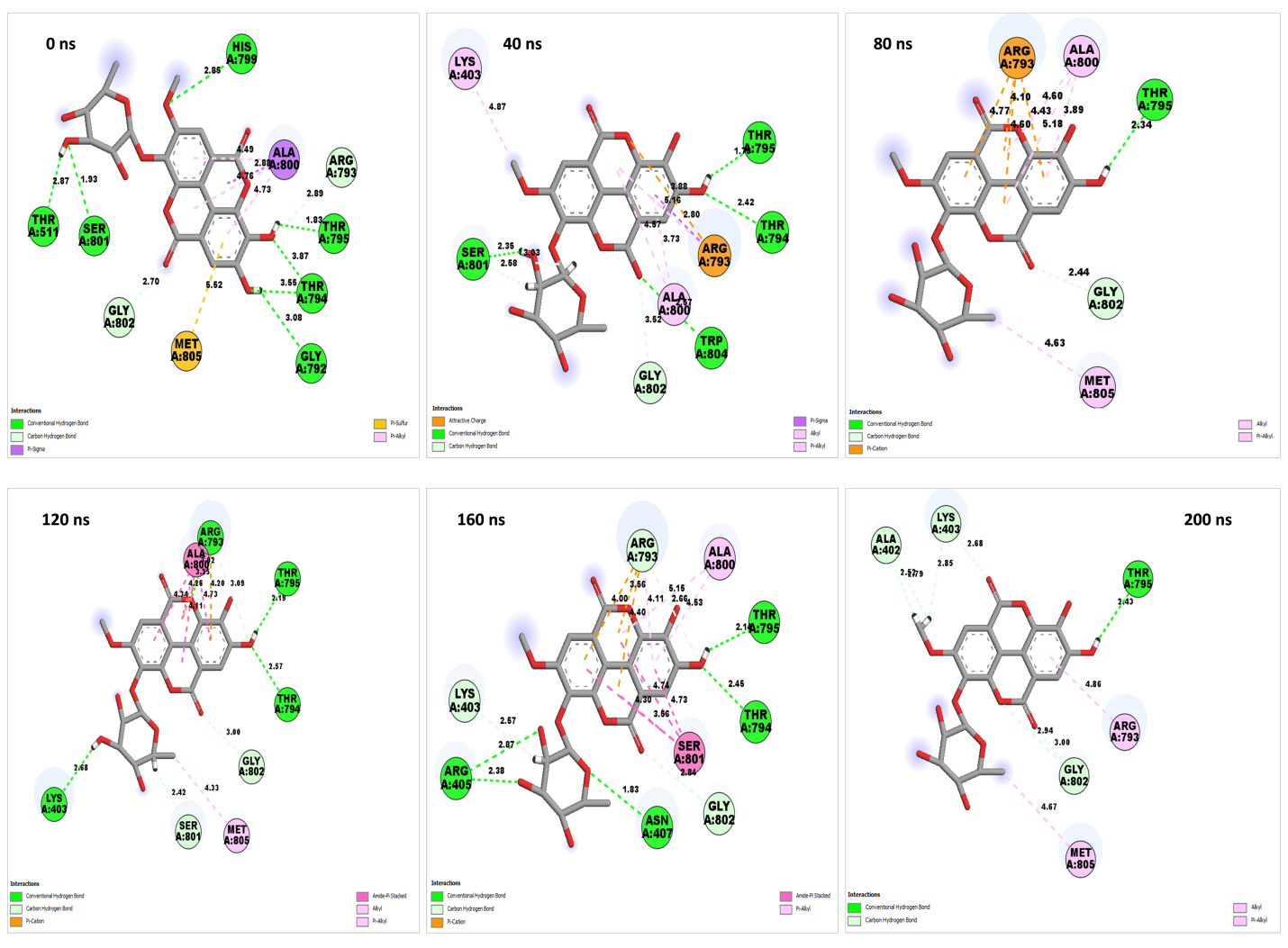


**S15 Fig. Binding poses of L2 ligand at the active site of NS5 protein at different time intervals during MD simulation.**
